# Asthma and COVID-19: a systematic review

**DOI:** 10.1186/s13223-020-00509-y

**Published:** 2021-01-06

**Authors:** Natália F. Mendes, Carlos P. Jara, Eli Mansour, Eliana P. Araújo, Licio A. Velloso

**Affiliations:** 1 School of Nursing, State University of Campinas, Campinas, Brazil; 2 Laboratory of Cell Signaling, Obesity and Comorbidities Research Center, State University of Campinas, Rua Carl Von Lineaus s/n, Instituto de Biologia, Bloco Z. Campus Universitário Zeferino Vaz, Barão Geraldo, Campinas, SP 13083-864 Brazil; 3 Clinical Immunology and Allergy, Department of Internal Medicine, State University of Campinas, Campinas, Brazil

**Keywords:** Coronavirus, SARS-CoV-2, Allergy, Respiratory insufficiency, Lung

## Abstract

**Background:**

Severe coronavirus disease-19 (COVID-19) presents with progressive dyspnea, which results from acute lung inflammatory edema leading to hypoxia. As with other infectious diseases that affect the respiratory tract, asthma has been cited as a potential risk factor for severe COVID-19. However, conflicting results have been published over the last few months and the putative association between these two diseases is still unproven.

**Methods:**

Here, we systematically reviewed all reports on COVID-19 published since its emergence in December 2019 to June 30, 2020, looking into the description of asthma as a premorbid condition, which could indicate its potential involvement in disease progression.

**Results:**

We found 372 articles describing the underlying diseases of 161,271 patients diagnosed with COVID-19. Asthma was reported as a premorbid condition in only 2623 patients accounting for 1.6% of all patients.

**Conclusions:**

As the global prevalence of asthma is 4.4%, we conclude that either asthma is not a premorbid condition that contributes to the development of COVID-19 or clinicians and researchers are not accurately describing the premorbidities in COVID-19 patients.

## Background

COVID-19 was first reported in December, 2019 in Wuhan, China, and rapidly spread across the globe [1]. It has affected more than 54 million people and has led to the death of over 1.3 million as of November 16, 2020 (www.who.org). Severely affected patients present fever, dry cough, dyspnea, and fatigue, which are commonly associated with the development of pneumonia and acute respiratory distress syndrome (ARDS) [2]. Advanced age, ischemic and congestive heart disease, hypertension, diabetes, and chronic obstructive pulmonary disease (COPD) are the most important independent predictors of death [2, 3]. As with other infectious diseases affecting the lungs, asthma has been cited as a potential risk factor for severe COVID-19 [4–8]. This association could be putatively explained on the basis of an abnormal immune response occurring in the context of the allergic condition and an abnormal respiratory function [9, 10]. However, no previous study has addressed this question looking into all studies that described the clinical features of COVID-19.

Here, we systematically reviewed all studies published on COVID-19 since its emergence in December 2019 to June 30, 2020, looking into the description of asthma as a premorbid condition and its putative association with severe progression of the disease. We show that out of 161,271 patients diagnosed with COVID-19 and having their premorbid conditions described, only 1.6% were reported as previously diagnosed with asthma.

## Methods

This is a systematic review of the diagnosis of asthma as a premorbid condition in patients with COVID-19. The report was organized according to the Preferred Reporting Items for Systematic Reviews [11]. Two authors, NFM and CPJ, independently identified cross-sectional and longitudinal studies published before June 30, 2020, that reported on the prevalence of asthma as a premorbid condition of severe COVID-19 by systematically searching PubMed-NCBI, Google Scholar, Scopus and Web of Science databases. As previously reported, PubMed-NCBI alone covers more than 90% of MEDLINE providing a widely accessible biomedical resource [12]. For database searches, language of the article was restricted to English. Search terms included the following: *COVID-19 (COVID, COVID 19)* or *nCov* or *novel coronavirus* or *Sars-Cov-2* in the title and *clinical characteristics* or *asthma* anywhere in the text. Three authors, EM, EPA, and LAV, resolved eventual discrepancies by discussion and adjudication.

We found 1069 articles that met the initial inclusion search criteria. All articles were assessed by authors and 598 were excluded (Additional file 1: Table 1) due to one or more of the following criteria: editorials; metanalyses; systematic reviews; commentaries; letters to the Editor; no description of patient’s clinical characteristics or premorbid conditions; duplicated articles and main text in a language other than English. We found 99 studies duplicated, which were also excluded accordingly, allowing us to analyze only in one of the both versions. The remaining 372 articles were included in the study. Additional file 1: Table 2 depicts the details of all articles analyzed.

Two authors, NFM and CPJ, independently extracted the following data from each article using a standardized form: study design; number of patients with COVID-19; mention of any respiratory disease; number of patients with any respiratory disease; mention of asthma; number of patients with the previous diagnosis of asthma. The entire body of the articles was presented descriptively.

## Results

**Fig. 1:**
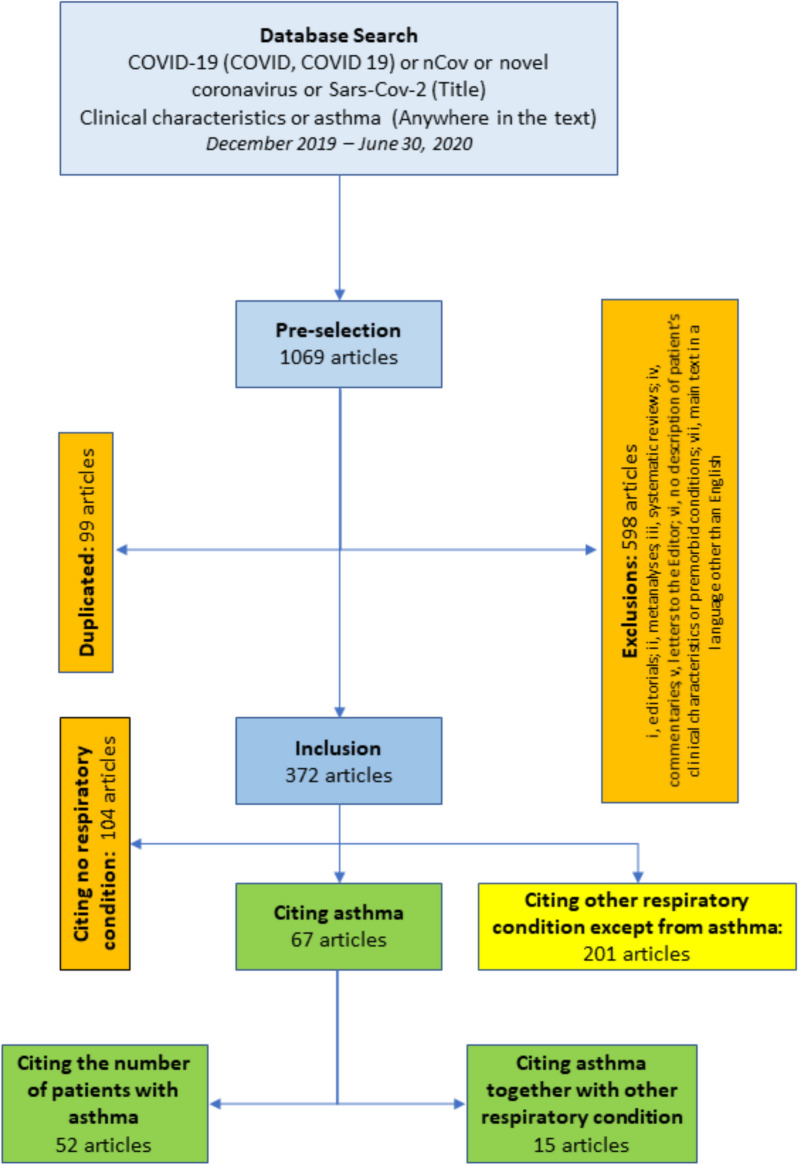
Schematic representation of search, exclusions and inclusions of articles

**Fig. 2:**
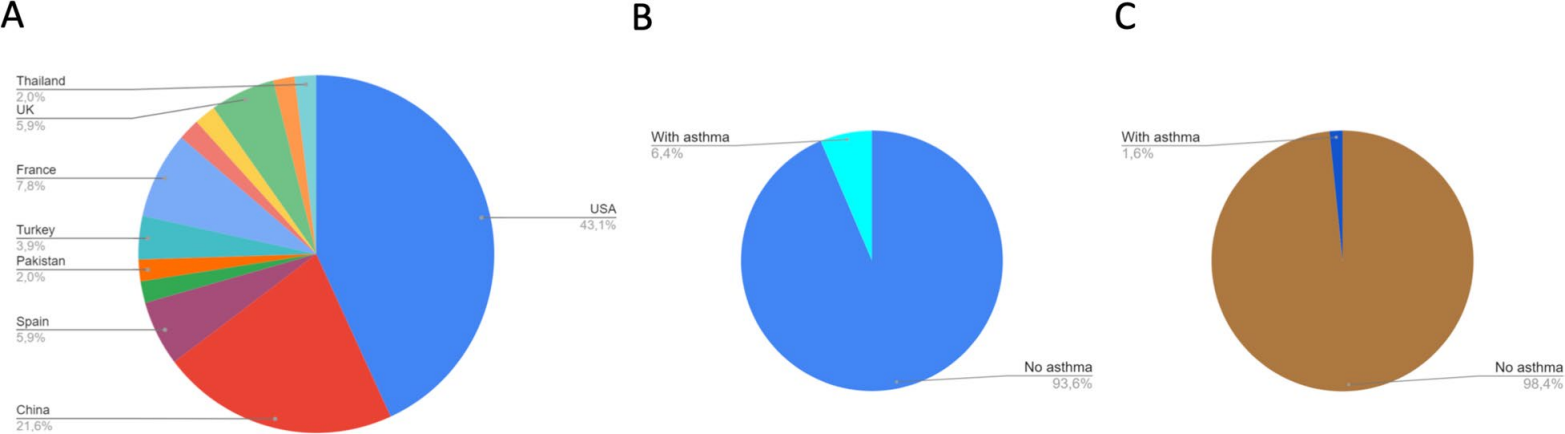
Graphic representation of the geographical origin of the studies analyzed in the systematic review (**a**) and the proportion of patients with previous diagnosis of asthma among COVID-19 patients included in studies citing asthma (**b**) and among all COVID-19 patients described up to June 30, 2020 (**c**)

Figure 1 is a schematic representation of search, inclusion and exclusion of articles. Our search criteria resulted in the identification of 1069 articles that were pre-selected for detailed analysis resulting in the exclusion of 598 articles (Additional file 1: Table 1) due to one or more of the following reasons: editorials; metanalyses; systematic reviews; commentaries; letters to the Editor; no description of patient’s clinical characteristics or premorbid conditions; and main text in a language other than English. The remaining 372 articles (Additional file 1: Table 2) described the clinical aspects of 161,271 COVID-19 patients. Two hundred and one studies mentioned the existence of other respiratory premorbidities except for asthma. Althought asthma was mentioned as a underlying disease in 67 studies, only 52 articles have described the exact number of the COVID-19 patients with asthma (Table 1). The other 15 studies presented asthma together with other respiratory diseases, making it impossible to identify the number of COVID-19 asthmatic patients. There was a total of 40,948 COVID-19 patients included in the studies mentioning asthma, of which 8439 were previously diagnosed with asthma. In most of the studies describing other respiratory illnesses, COPD was the leading diagnosis. The United States was the country with the largest number of studies describing asthma, followed by China, France, Spain and the United Kingdom (Fig. 2a).

**Table 1:** Details of the articles that mention asthma

Citation	Title	DOI	Mention to respiratory disease except from asthma	Number (%) respiratory disease except from asthma	Mention to asthma	Number (%) asthma patients	Number of COVID-19 patients
Alkundi A, et al..	Clinical characteristics and outcomes of COVID-19 hospitalized patients with diabetes in the United Kingdom: A retrospective single centre study	10.1016/j.diabres.2020.108263	Yes	COPD: 7 (7.3%)	Yes	6 (2.6%)	232
Alsofayan YM, et al.	Clinical characteristics of COVID-19 in Saudi Arabia: A national retrospective study	10.1016/j.jiph.2020.05.026	Yes	Chronic lung disease: 57 (5.2%)	Yes	54 (4.9%)	1519
Aretz M, et al.	Characteristics and Outcomes of 21 Critically Ill Patients With COVID-19 in Washington State	10.1001/jama.2020.4326	Yes	COPD: 7 (33.3%)	Yes	2 (9.1%)	21
Asghar MS, et al.	Clinical Profiles, Characteristics, and Outcomes of the First 100 Admitted COVID-19 Patients in Pakistan: A Single-Center Retrospective Study in a Tertiary Care Hospital of Karachi	10.7759/cureus.8712	Yes	COPD: 3 (3%)	Yes	2 (2%)	100
Benger M, et al.	Intracerebral haemorrhage and COVID-19: Clinical characteristics from a case series	10.1016/j.bbi.2020.06.005	No	–	Yes	1 (20%)	5
Bhatraju PK, et al.	Covid-19 in Critically Ill Patients in the Seattle Region — Case Series	10.1056/NEJMoa2004500	Yes	COPD: 1 (4%)	Yes	3 (12.5%)	24
Chao JY, et al.	Clinical Characteristics and Outcomes of Hospitalized and Critically Ill Children and Adolescents with Coronavirus Disease 2019 (COVID-19) at a Tertiary Care Medical Center in New York City	10.1016/j.jpeds.2020.05.006	No	–	Yes	11 (24.4%)	46
Cheng FY, et al.	Using Machine Learning to Predict ICU Transfer in Hospitalized COVID-19 Patients	10.3390/jcm9061668	Yes	COPD: 219 (8.42%)	Yes	219 (8.42%)	2599
Cheung ZB and Forsh DA	Early outcomes after hip fracture surgery in COVID-19 patients in New York City	10.1016/j.jor.2020.06.003	Yes	COPD: 1 (10%)	Yes	2 (20%)	10
Chhiba KD, et al.	Prevalence and characterization of asthma in hospitalized and nonhospitalized patients with COVID-19	10.1016/j.jaci.2020.06.010	Yes	COPD: 111 (7.27%)	Yes	220 (14.2%)	1542
D'Silva KM, et al.	Clinical characteristics and outcomes of patients with coronavirus disease 2019 (COVID-19) and rheumatic disease: a comparative cohort study from a US 'hot spot'	10.1136/annrheumdis-2020-217888	Yes	COPD: 9 (5.7%)	Yes	31 (19.8%)	156
Du H, et al.	Clinical characteristics of 182 pediatric COVID-19 patients with different severities and allergic status	10.1111/all.14452	No	–	Yes	1 (2.3%)	43
Duanmu Y, et al.	Characteristics of Emergency Department Patients With COVID-19 at a Single Site in Northern California: Clinical Observations and Public Health Implications	10.1111/acem.14003	Yes	COPD: 1 (10%)	Yes	10 (10%)	100
Fan J, et al.	The epidemiology of reverse transmission of COVID-19 in Gansu Province, China	10.1016/j.tmaid.2020.101741	Yes	COPD: N/A	Yes	N/A	37
Fernandéz R, et al.	COVID-19 in Solid Organ Transplant Recipients: A Single-Center Case Series from Spain	10.1111/ajt.15929	No	–	Yes	1 (5.55%)	18
Gayam V, et al.	Presenting characteristics, comorbidities, and outcomes of patients coinfected with COVID-19 and Mycoplasma pneumoniae in the USA	10.1002/jmv.26026	No	–	Yes	2 (33.3%)	6
Gold JAW, et al.	Characteristics and Clinical Outcomes of Adult Patients Hospitalized With COVID-19—Georgia, March 2020	10.15585/mmwr.mm6918e1	Yes	COPD: 16 (5.2%)	Yes	32 (10.5%)	305
Goyal P, et al.	Clinical Characteristics of Covid-19 in New York City	10.1056/NEJMc2010419	Yes	COPD: 20 (5.1%)	Yes	49 (12.5%)	393
Huang D, et al.	A novel risk score to predict diagnosis with coronavirus disease 2019 (COVID-19) in suspected patients: A retrospective, multicenter, and observational study	10.1002/jmv.26143	Yes	COPD: 9 (2.7%)	Yes	5 (1.5%)	336
Jehi L, et al.	Individualizing Risk Prediction for Positive Coronavirus Disease 2019 Testing: Results From 11,672 Patients	10.1016/j.chest.2020.05.580	Yes	COPD/emphysema: 14 (1.26%)	Yes	163 (14.7%)	1108
Kaushik S, et al.	Multisystem Inflammatory Syndrome in Children Associated with Severe Acute Respiratory Syndrome Coronavirus 2 Infection (MIS-C): A Multi-institutional Study from New York City	10.1016/j.jpeds.2020.06.045	No	–	Yes	5 (15%)	33
Knight M, et al.	Characteristics and outcomes of pregnant women admitted to hospital with confirmed SARS-CoV-2 infection in UK: national population-based cohort study	10.1136/bmj.m2107	No	–	Yes	31 (7%)	427
Korkmaz MF, et al.	The Epidemiological and Clinical Characteristics of 81 Children with COVID-19 in a Pandemic Hospital in Turkey: an Observational Cohort Study	10.3346/jkms.2020.35.e236	No	–	Yes	1 (1.23%)	81
Lechien JR, et al.. 2020	Clinical and Epidemiological Characteristics of 1,420 European Patients With Mild-To-Moderate Coronavirus Disease 2019	10.1111/joim.13089	Yes	Respiratory insufficiency: 10 (0.7%)	Yes	93 (6.5%)	1420
Li X, et al.. 2020	Risk factors for severity and mortality in adult COVID-19 inpatients in Wuhan	10.1016/j.jaci.2020.04.006	Yes	COPD: 17 (3.1%)	Yes	5 (0.9%)	548
Liabeuf S, et al.	Association between renin-angiotensin system inhibitors and COVID-19 complications	10.1093/ehjcvp/pvaa062	Yes	COPD: 26 (10%)	Yes	14 (5%)	268
Liu BM, et al.	Epidemiological characteristics of COVID-19 patients in convalescence period	10.1017/S0950268820001181	Yes	Pulmonary tuberculosis: 4 (5.9%)	Yes	1 (1.5%)	68
Liu D, et al.	The pulmonary sequalae in discharged patients with COVID-19: a short-term observational study	10.1186/s12931-020.01385-1	No	–	Yes	4 (2.8%)	149
Lokken EM, et al.	Clinical characteristics of 46 pregnant women with a severe acute respiratory syndrome coronavirus 2 infection in Washington State	10.1016/j.ajog.2020.05.031	No	–	Yes	4 (8.7%)	46
Magagnoli et al.	Outcomes of Hydroxychloroquine Usage in United States Veterans Hospitalized with COVID-19	10.1016/j.medj.2020.06.001	Yes	COPD: 175 (21.68%)	Yes	40 (4.95%)	807
Mestre-Goméz B, et al.	Incidence of pulmonary embolism in non‑critically ill COVID‑19 patients. Predicting factors for a challenging diagnosis	10.1007/s11239-020-02190-9	Yes	Chronic obstrutive lung disease: 13 (14.28%)	Yes	7 (7.69%)	91
Mikami T, et al.	Risk Factors for Mortality in Patients with COVID-19 in New York City	10.1007/s11606-020-05983-z	Yes	COPD: 176 (2.7%)	Yes	271 (4.2%)	6493
Mitra AR, et al.	Baseline characteristics and outcomes of patients with COVID-19 admitted to intensive care units in Vancouver, Canada: a case series	10.1503/cmaj.200794	Yes	COPD: 8 (6.8%)	Yes	14 (12%)	117
Oualha M, et al.	Severe and fatal forms of COVID-19 in children	10.1016/j.arcped.2020.05.010	Yes	COPD: 1 (3.7%) e Chronic lung disease: 2 (7.4%)	Yes	1 (3.7%)	27
Pare JR, et al.	Point-of-care Lung Ultrasound Is More Sensitive than Chest Radiograph for Evaluation of COVID-19	10.5811/westjem.2020.5.47743	Yes	COPD: 1 (3.7%)	Yes	4 (14.8%)	27
Peyrony O, et al.	Accuracy of Emergency Department Clinical Findings for Diagnosis of Coronavirus Disease 2019	10.1016/j.annemergmed.2020.05.022	Yes	COPD: 24 (6.2%)	Yes	22 (5.7%)	391
Phipps MM, et al.	Acute Liver Injury in COVID-19: Prevalence and Association with Clinical Outcomes in a Large US Cohort	10.1002/hep.31404	Yes	COPD: 185 (8.1%)	Yes	308 (14)	2273
Pongpirul WA, et al.	Clinical Characteristics of Patients Hospitalized with Coronavirus Disease, Thailand	10.3201/eid2607.200598	Yes	COPD: 0	Yes	0	11
Price-Haywood EG, et al.	Hospitalization and Mortality among Black Patients and White Patients with Covid-19	10.1056/NEJMsa2011686	Yes	COPD: 79 (2.25%)	Yes	147 (4%)	3481
Richardson S, et al.	Presenting Characteristics, Comorbidities, and Outcomes Among 5700 Patients Hospitalized With COVID-19 in the New York City Area	10.1001/jama.2020.6775	Yes	COPD: 287 (5.4%)	Yes	479 (9%)	5700
San-Juan R, et al.	Incidence and clinical profiles of COVID-19 pneumonia in pregnant women: A single-centre cohort study from Spain	10.1016/j.eclinm.2020.100407	No	–	Yes	4 (12.5%)	32
Satici C, et al.	Performance of pneumonia severity index and CURB-65 in predicting 30-day mortality in patients with COVID-19	10.1016/j.ijid.2020.06.038	Yes	COPD: 28 (4.1%)	Yes	43 (6.3%)	681
Sentilhes L, et al.	Coronavirus disease 2019 in pregnancy was associated with maternal morbidity and preterm birth	10.1016/j.ajog.2020.06.022	No	–	Yes	5 (9.3%)	54
Shahriarirad R, et al.	Epidemiological and clinical features of 2019 novel coronavirus diseases (COVID-19) in the South of Iran	10.1186/s12879-020-05128-x	Yes	COPD: 9 (8%)	Yes	7 (6.2%)	113
Smith SM, et al.	Impaired glucose metabolism in patients with diabetes, prediabetes and obesity is associated with severe Covid-19	10.1002/jmv.26227	Yes	COPD: 12 (6.5%)	Yes	18 (9.8%)	184
Solís and Carreňo	COVID-19 Fatality and Comorbidity Risk Factors among Diagnosed Patients in Mexico	10.1101/2020.04.21.20074591	Yes	COPD: 202 (2.7%)	Yes	270 (3.6%)	7497
Sultan I, et al.	The Role of Extracorporeal Life Support for Patients With COVID-19: Preliminary Results from a Statewide Experience	10.1111/jocs.14583	No	–	Yes	N/A	10
Wang X, et al.	Nosocomial Outbreak of 2019 Novel Coronavirus Pneumonia in Wuhan, China	10.1183/13993003.00544-2020	No	–	Yes	2 (5.7%)	35
Zhang C, et al.	Clinical and epidemiological characteristics of pediatric SARS-CoV-2 infections in China: A multicenter case series	10.1371/journal.pmed.1003130	No	–	Yes	1 (3%)	34
Zhang JJ, et al.	Clinical characteristics of 140 patients infected with SARS-CoV-2 in Wuhan, China	10.1111/all.14238	Yes	COPD: 2 (1.4%)	Yes	0	140
Zhao M, et al.	Comparison of clinical characteristics and outcomes of patients with coronavirus disease 2019 at different ages	10.18632/aging.103298	Yes	COPD: 23 (2.3%)	Yes	12 (1.2%)	1000
Zhou X, et al.	Clinical Characteristics of Coronavirus Disease 2019 (COVID-19) Patients with Hypertension on Renin-Angiotensin System Inhibitors	10.1080/10641963.2020.1764018	Yes	COPD:3 (2.7%)	Yes	1 (0.9%)	110

Thus, according to current COVID-19 clinical records, 6.4% of patients included in articles describing the clinical characteristics of COVID-19 patients and citing asthma were previously diagnosed with asthma (Fig. 2b). If all studies providing any clinical description of COVID-19 comorbidities are taken into consideration, asthma was present in only 1.6% of patients (Fig. 2c).

## Discussion

Asthma is a highly prevalent, chronic, non-communicable disease that affects up to 4.4% of the world’s population (http://www.globalasthmareport.org; https://www.who.int/news-room/q-a-detail/asthma). Its recurrent nature leads to frequent hospitalizations and high mortality, ranging from 2 to 4/100,000 [13]. Respiratory viruses can trigger asthma exacerbations, which can increase the severity of the infectious condition [14]. In the past, coronaviruses have been implicated as triggers of asthma exacerbations [15, 16]; this is also true for influenza virus [17]. However, as for the new coronavirus, SARS-CoV-2, there is still controversy regarding the putative role of asthma as a premorbid that could worsen disease progression [7, 8, 18].

Here, we evaluated all studies on COVID-19 published since its emergence up to June 30, 2020. We showed that asthma was described as a premorbid condition in only 1.6% of all patients. These numbers are far less than expected considering the prevalence of asthma in the world (http://www.globalasthmareport.org; https://www.who.int/news-room/q-a-detail/asthma) and could suggest that having asthma as a premorbid condition either represents no risk for COVID-19 or could be a protective factor against the development of the disease. However, there are some aspects that should be considered as potentially impactful for the findings herein reported. First, the prevalence of asthma varies across the globe, ranging from 21% in Australia to less than 2% in China, Kazakhstan and Vietnam [19]. Likewise, the most common risk factors for COVID-19, obesity, diabetes and hypertension, have distinct prevalences in different countries (www.who.org). Thus, the geographical origin of the studies could have influenced the results. However, as the studies included in this systematic review were mostly originated from countries presenting a wide range of prevalences for both asthma and the main comorbidities for COVID-19, we believe this factor plays a minor role in the reported findings.

Another aspect that could explain our results is that asthma treatment with inhaled corticosteroids allied to improved therapeutic and prophylactic adhesion has increased over the years, resulting in the reduction of respiratory distress episodes and allergy associated immunological imbalance [20–23]. Moreover, allergy and asthma international associations were efficient to rapidly produce and release COVID-19 guidelines that provided advice for health professionals involved in the care of asthma patients, as well as for reaching the general public [24–27]. These actions could have beneficially impacted on the control of asthma and also influenced patients to follow social isolation procedures; thus, mitigating the risk of contracting COVID-19.

It has been suggested that the particular inflammatory environment in the bronchioalveolar system of asthma patients could lead to a reduced expression of SARS-CoV-2 receptor, angiotensin converting enzyme 2 (ACE2), rendering asthma patients protected from the infection [28–30]. This could be due to the fact that interleukin-13 (IL-13), a cytokine involved in eosinophil recruitment to the bronchial epithelia [31], is capable of reducing ACE2 expression in bronchial ex-vivo human samples [28]. In line with these findings, it has been reported that progressive increase in blood eosinophil counts is related to COVID-19 recovery. Thus, if proven correct, these data could suggest that only patients with allergic asthma are protected from COVID-19, as recently suggested [32, 33]. However, currently available data provides no sufficient detail regarding asthma etiological classification and further studies would be required in order to provide advance in this issue.

The main weaknesses of this systematic review rely on the facts that we included publications covering the initial 6 months of pandemics and as new data is published on a daily basis, some changes in the frequency of asthma could appear; moreover, readers should keep in mind that some reports show that in certain pocket populations, asthma could be an important comorbidity for COVID-19 [34]. The reasons for these apparent discrepancies should be a focus of further studies.

Thus, as for the data analyzed in this systematic review, asthma does not seem to be an important premorbid condition in COVID-19 patients; or, conversely, it could be a protective factor, as previously proposed [18]. The findings herein reported could be an epidemiological truth that should be further explored in mechanistic studies or could be due to the fact that researchers are not properly investigating and describing the premorbidities in COVID-19 patients. Whatever the reasons, the medical community should be aware of the implications of missing the diagnosis of a potentially severe respiratory disease such as asthma that could worsen the prognosis of COVID-19 patients.

## Supplementary Information

**Additional file 1: Table 1**. Excluded articles. **Table 2.** Included articles.

## Data Availability

Data are available upon request.
