# Supplementary material for: Asthma and COVID-19: a systematic review
Source: Allergy Asthma Clin Immunol. 2021 Jan 6;17:5. doi: 10.1186/s13223-020-00509-y (PMC7787409; doi:10.1186/s13223-020-00509-y)
Supplement: Supplementary file 1 — Additional file 1: Table 1. Excluded articles. Table 2. Included articles. [file 13223_2020_509_MOESM1_ESM.docx]

Additional data

Asthma and COVID-19 - *A systematic review*

Natália F. Mendes^1,2^ , Carlos P. Jara^1,2^ , Eli Mansour^3^, Eliana P. Araújo^1,2^ , Licio A. Velloso^2,3*^

^1^School of Nursing, State University of Campinas, Campinas, Brazil

^2^Laboratory of Cell Signaling, Obesity and Comorbidities Research Center, State University of Campinas, Campinas, Brazil

^3^Clinical Immunology and Allergy, Department of Internal Medicine, State University of Campinas, Campinas, Brazil

**Additional Table 1. Excluded articles.**

| Citation | Title | DOI | Type of article | Reason for exclusion |
| --- | --- | --- | --- | --- |
| Andino JJ et al. | Video Visits as a Substitute for Urological Clinic Visits | 10.1016/j.urology.2020.05.080 | Original Research | Do not present patient' underlying diseases |
| Assaad S, et al. | High mortality rate in cancer patients with symptoms of COVID-19 with or without detectable SARS-COV-2 on RT-PCR | 10.1016/j.ejca.2020.05.028 | Original Article | Do not present patient' underlying diseases |
| Avula A, et al. | COVID-19 Presenting as Stroke | 10.1016/j.bbi.2020.04.077 | Original Article | Do not present patient' underlying diseases |
| Banerjee A et al. | Use of Machine Learning and Artificial Intelligence to predict SARS-CoV-2 infection from Full Blood Counts in a population | 10.1016/j.intimp.2020.106705 | Original Research | Do not present patient' underlying diseases |
| Bi X, et al. | Prediction of Severe Illness Due to COVID-19 Based on an Analysis of Initial Fibrinogen to Albumin Ratio and Platelet Count | 10.1080/09537104.2020.1760230 | Original Article | Do not present patient' underlying diseases |
| Bielecki M, et al. | Social distancing alters the clinical course of COVID-19 in young adults: A comparative cohort study | 10.1093/cid/ciaa889 | Original research | Do not present patient' underlying diseases |
| Bode B, et al. | Glycemic Characteristics and Clinical Outcomes of COVID-19 Patients Hospitalized in the United States | 10.1177/1932296820924469 | Original Research | Do not present patient' underlying diseases |
| Cao D, et al. | Clinical analysis of ten pregnant women with COVID-19 in Wuhan, China: A retrospective study | 10.1016/j.ijid.2020.04.047 | Original Article | Do not present patient' underlying diseases |
| Chan KH, et al. | Clinical characteristics and outcome in patients with combined diabetic ketoacidosis and hyperosmolar hyperglycemic state associated with COVID-19: A retrospective, hospital-based observational case series | 10.1016/j.diabres.2020.108279 | Original research | Do not present patient' underlying diseases |
| Chang CM, et al. | COVID-19: Taiwan's epidemiological characteristics and public and hospital responses | 10.7717/peerj.9360 | Original research | Do not present patient' underlying diseases |
| Chang D, et al. | Epidemiologic and Clinical Characteristics of Novel Coronavirus Infections Involving 13 Patients Outside Wuhan, China | 10.1001/jama.2020.1623 | Research Letter | Do not present patient' underlying diseases |
| Chen C, et al. | A special symptom of olfactory dysfunction in coronavirus disease 2019: report of three cases | 10.1007/s13365-020-00849-w | Case Reports | Do not present patient' underlying diseases |
| Chen D, et al. | Assessment of Hypokalemia and Clinical Characteristics in PatientsWith Coronavirus Disease 2019 in Wenzhou, China | 10.1001/jamanetworkopen.2020.11122 | Original Article | Do not present patient' underlying diseases |
| Chen L, et al. | Ocular Manifestations of a Hospitalised Patient with Confirmed 2019 Novel Coronavirus Disease | 10.1136/bjophthalmol-2020-316304 | Original Research | Do not present patient' underlying diseases |
| Chen M, et al. | A SARS-CoV-2 familial cluster infection reveals asymptomatic transmission to children | 10.1016/j.jiph.2020.05.018 | Original Article | Do not present patient' underlying diseases |
| Chen M, et al. | Clinical Characteristics of Re-hospitalized Patients With COVID-19 in China | 10.1002/jmv.26002 | Research Article | Do not present patient' underlying diseases |
| Chen P, et al. | Epidemiological and Clinical Characteristics of 136 Cases of COVID-19 in Main District of Chongqing | 10.1016/j.jfma.2020.04.019 | Original Research | Do not present patient' underlying diseases |
| Chen R, et al. | Safety and efficacy of different anesthetic regimens for parturients with COVID-19 undergoing Cesarean delivery: a case series of 17 patients | 10.1007/s12630-020-01630-7 | Original Research | Do not present patient' underlying diseases |
| Chen T, et al. | Epidemic characteristics of the COVID-19 outbreak in Tianjin, a well-developed city in China | 10.1016/j.ajic.2020.06.006 | Original Article | Do not present patient' underlying diseases |
| Chen Z, et al. | Childhood COVID-19: a multicentre retrospective study | 10.1016/j.cmi.2020.06.015 | Research note | Do not present patient' underlying diseases |
| Chu J, et al. | Clinical Characteristics of 54 Medical Staff With COVID-19: A Retrospective Study in a Single Center in Wuhan, China | 10.1002/jmv.25793 | Original Research | Do not present patient' underlying diseases |
| Cobes N, et al. | Ventilation/perfusion SPECT/CT findings in different lung lesions associated with COVID-19: a case series | 10.1007/s00259-020-04920-w | Short communication | Do not present patient' underlying diseases |
| Colizzi M, et al. | Psychosocial and Behavioral Impact of COVID-19 in Autism Spectrum Disorder: An Online Parent Survey | 10.3390/brainsci10060341 | Original Article | Do not present patient' underlying diseases |
| Dai H, et al. | High-resolution Chest CT Features and Clinical Characteristics of Patients Infected with COVID-19 in Jiangsu, China | 10.1016/j.ijid.2020.04.003 | Original Research | Do not present patient' underlying diseases |
| De Gottardi A, et al. | Clinical characteristics and management of a liver transplanted patient admitted with SARS-CoV-2 infection | 10.1016/j.clinre.2020.05.014 | Case Report | Do not present patient' underlying diseases |
| Dong Y, et al. | Epidemiology of COVID-19 Among Children in China | 10.1542/peds.2020-0702 | Original research | Do not present patient' underlying diseases |
| Dong Y, et al. | A novel simple scoring model for predicting severity of patients with SARS-CoV-2 infection | 10.1111/tbed.13651 | Original research | Do not present patient' underlying diseases |
| Du M, et al. | Multiomics Evaluation of Gastrointestinal and Other Clinical Characteristics of COVID-19 | 10.1053/j.gastro.2020.03.045 | Original research | Do not present patient' underlying diseases |
| Du W, et al. | Persistence of SARS-CoV-2 virus RNA in feces: A case series of children | 10.1016/j.jiph.2020.05.025 | Original research | Do not present patient' underlying diseases |
| Dworzańska A, et al. | A 56-year-old man with RT-PCR negative nasopharyngeal swabs with Coronavirus Disease 2019 (COVID-19) Pneumonia | 10.26444/aaem/123543 | Case Reports | Do not present patient' underlying diseases |
| Fernandez-Nieto D, et al. | Clinical and Histological Characterization of Vesicular COVID-19 Rashes: A Prospective Study in a Tertiary Care Hospital | 10.1111/ced.14277 | Concise Report | Do not present patient' underlying diseases |
| Finzi E | Treatment of SARS-CoV-2 with high dose oral zinc salts: A report on four patients | 10.1016/j.ijid.2020.06.006 | Case report | Do not present patient' underlying diseases |
| Frey MK, et al. | Impact of the coronavirus disease 2019 pandemic on the quality of life for women with ovarian cancer | 10.1016/j.ajog.2020.06.049 | Original research | Do not present patient' underlying diseases |
| Gómez-Iglesias P, et al. | An Online Observational Study of Patients With Olfactory and Gustory Alterations Secondary to SARS-CoV-2 Infection | 10.3389/fpubh.2020.00243 | Original research | Do not present patient' underlying diseases |
| Gualtieri P, et al. | Body Composition Findings by Computed Tomography in SARS-CoV-2 Patients: Increased Risk of Muscle Wasting in Obesity | 10.3390/ijms21134670 | Original research | Do not present patient' underlying diseases |
| Güemes-Villahoz N, et al. | Detecting SARS-CoV-2 RNA in conjunctival secretions: Is it a valuable diagnostic method of COVID-19? | 10.1002/jmv.26219 | Original research | Do not present patient' underlying diseases |
| Guo H, et al. | The impact of the COVID-19 epidemic on the utilization of emergency dental services. | 10.1016/j.jds.2020.02.002 | Short communication | Do not present patient' underlying diseases |
| Guo L et al. | Absence of SARS-CoV-2 in semen of a COVID-19 patient cohort | 10.1111/andr.12848 | Original research | Do not present patient' underlying diseases |
| He D, et al. | Comparing COVID-19 and the 1918-19 influenza pandemics in the United Kingdom | 10.1016/j.ijid.2020.06.075 | Comparative Study | Do not present patient' underlying diseases |
| He YF, et al. | Clinical characteristics, diagnosis, and treatment of COVID-19: A case report. | 10.12998/wjcc.v8.i11.2325 | Case Reports | Do not present patient' underlying diseases |
| Himoto Y, et al. | Diagnostic Performance of Chest CT to Differentiate COVID-19 Pneumonia in Non-High-Epidemic Area in Japan | 10.1007/s11604-020-00958-w | Original research | Do not present patient' underlying diseases |
| Hu X et al. | Clinical features and chest CT findings of 3 cases of 2019 novel coronavirus (COVID-19) pneumonia | 10.1016/j.radcr.2020.06.031 | Case Reports | Do not present patient' underlying diseases |
| Hu Z, et al. | Clinical Characteristics of 24 Asymptomatic Infections With COVID-19 Screened Among Close Contacts in Nanjing, China | 10.1007/s11427-020-1661-4 | Research Article | Do not present patient' underlying diseases |
| Hua CZ, et al. | Epidemiological features and viral shedding in children with SARS-CoV-2 infection | 10.1002/jmv.26180 | Original research | Do not present patient' underlying diseases |
| Huang H, et al. | Clinical characteristics of COVID-19 in patients with preexisting ILD: A retrospective study in a single center in Wuhan, China | 10.1002/jmv.26174 | Original Article | Do not present patient' underlying diseases |
| Huang JT, et al. | Chronological Changes of Viral Shedding in Adult Inpatients with COVID-19 in Wuhan, China | 10.1093/cid/ciaa631 | Original research | Do not present patient' underlying diseases |
| Huang K et al. | A retrospective analysis of the epidemiology, clinical manifestations, and imaging characteristics of familial cluster-onset COVID-19 | 10.21037/atm-20-3759 | Original Research | Do not present patient' underlying diseases |
| Huang L, et al. | Initial CT Imaging Characters of an Imported Family Cluster of COVID-19 | 10.1016/j.clinimag.2020.04.010 | Original Article | Do not present patient' underlying diseases |
| Ji T, et al. | Lockdown Contained the Spread of 2019 Novel Coronavirus Disease in Huangshi City, China: Early Epidemiological Findings | 10.1093/cid/ciaa390 | Original Research | Do not present patient' underlying diseases |
| Jia J, et al. | Epidemiological Characteristics on the Clustering Nature of COVID-19 in Qingdao City, 2020: A Descriptive Analysis | 10.1017/dmp.2020.59 | Original Research | Do not present patient' underlying diseases |
| Jiang Q, et al. | The Prevalence, Characteristics, and Prevention Status of Skin Injury Caused by Personal Protective Equipment Among Medical Staff in Fighting COVID-19: A Multicenter, Cross-Sectional Study | 10.1089/wound.2020.1212 | Original Article | Do not present patient' underlying diseases |
| Jiang S et al. | Liver Injury in Critically Ill and Non-critically Ill COVID-19 Patients: A Multicenter, Retrospective, Observational Study | 10.3389/fmed.2020.00347 | Original Research | Do not present patient' underlying diseases |
| Joffily L, et al. | The close relationship between sudden loss of smell and COVID-19 | 10.1016/j.bjorl.2020.05.002 | Original research | Do not present patient' underlying diseases |
| Jung SM, et al. | Epidemiological Identification of A Novel Pathogen in Real Time: Analysis of the Atypical Pneumonia Outbreak in Wuhan, China, 2019-2020 | 10.3390/jcm9030637 | Original Research | Do not present patient' underlying diseases |
| Khamis F, et al. | Epidemiology of COVID-19 Infection in Oman: Analysis of the First 1304 Cases | 10.5001/omj.2020.60 | Original Research | Do not present patient' underlying diseases |
| Kim Gu, et al. | Clinical Characteristics of Asymptomatic and Symptomatic Patients With Mild COVID-19 | 10.1016/j.cmi.2020.04.040 | Original Article | Do not present patient' underlying diseases |
| Kobayashi KI, et al. | Clinical Characteristics of Patients With Coronavirus Disease 2019 in Japan: A Single- Center Case Series | 10.1093/infdis/jiaa244 | Case Reports | Do not present patient' underlying diseases |
| Kolonko A, et al. | A single-center experience with patients immediately after transplantation | 10.1111/tid.13381 | Case Report | Do not present patient' underlying diseases |
| Kurstjens S, et al. | Rapid identification of SARS-CoV-2-infected patients at the emergency department using routine testing | 10.1515/cclm-2020-0593 | Multicenter study | Do not present patient' underlying diseases |
| Lang G, et al. | The Clinical and Radiological Manifestations in Coronavirus Disease 2019 With Negative Nucleic Acid Results | 10.1093/ofid/ofaa252 | Original research | Do not present patient' underlying diseases |
| Lapostolle F, et al. | Clinical features of 1487 COVID-19 patients with outpatient management in the Greater Paris: the COVID-call study | 10.1007/s11739-020-02379-z | Original research | Do not present patient' underlying diseases |
| Lei P, et al. | Clinical and Computed Tomographic (CT) Images Characteristics in the Patients With COVID-19 Infection: What Should Radiologists Need to Know? | 10.3233/XST-200670 | Research Article | Do not present patient' underlying diseases |
| Li B, et al. | Diagnostic Value and Key Features of Computed Tomography in Coronavirus Disease 2019 | 10.1080/22221751.2020.1750307 | Original research | Do not present patient' underlying diseases |
| Li C, et al. | Chest CT study of fifteen COVID-19 patients with positive RT-PCR retest results after discharge | 10.21037/qims-20-530 | Original research | Do not present patient' underlying diseases |
| Li et al. | Efficacy and Safety of Lopinavir/Ritonavir or Arbidol in Adult Patients with Mild/Moderate COVID-19: An Exploratory Randomized Controlled Trial | 10.1016/j.medj.2020.04.001 | Original research | Do not present patient' underlying diseases |
| Li H, et al. | The Profile of Peripheral Blood Lymphocyte Subsets and Serum Cytokines in Children With 2019 Novel Coronavirus Pneumonia | 10.1016/j.jinf.2020.04.001 | Original Article | Do not present patient' underlying diseases |
| Li J, et al. | Clinical characteristics of emergency surgery patients infected with coronavirus disease 2019 (COVID-19) pneumonia in Wuhan, China | 10.1016/j.surg.2020.05.007 | Research Article | Do not present patient' underlying diseases |
| Li R, et al. | Clinical characteristics of 225 patients with COVID-19 in a tertiary Hospital near Wuhan, China | 10.1016/j.jcv.2020.104363 | Original research | Do not present patient' underlying diseases |
| Li W, et al. | The Characteristics of Household Transmission of COVID-19 | 10.1093/cid/ciaa450 | Original Research | Do not present patient' underlying diseases |
| Li X, et al. | Epidemiological Characteristics of Confirmed COVID-19 in Guizhou Province, China | 10.1017/dmp.2020.134. | Original Article | Do not present patient' underlying diseases |
| Li X, et al. | CT Imaging Changes of Corona Virus Disease 2019 (COVID-19): A Multi-Center Study in Southwest China | 10.1186/s12967-020-02324-w | Original Research | Do not present patient' underlying diseases |
| Li Y, et al. | Comparison of hospitalized patients with pneumonia caused by COVID-19 and influenza A in children under 5 years | 10.1016/j.ijid.2020.06.026 | Original Article | Do not present patient' underlying diseases |
| Li Y, et al. | Retrospective analysis of laboratory testing in 54 patients with severe- or critical-type 2019 novel coronavirus pneumonia | 10.1038/s41374-020-0431-6 | Original research | Do not present patient' underlying diseases |
| Li Y, et al. | Characteristics of Respiratory Virus Infection During the Outbreak of 2019 Novel Coronavirus in Beijing | 10.1016/j.ijid.2020.05.008 | Original Article | Do not present patient' underlying diseases |
| Li Y, et al. | Insight Into COVID-2019 for Pediatricians | 10.1002/ppul.24734 | Case Report | Do not present patient' underlying diseases |
| Liao M, et al. | Single-cell landscape of bronchoalveolar immune cells in patients with COVID-19 | 10.1038/s41591-020-0901-9 | Brief Communication | Do not present patient' underlying diseases |
| Lin P, et al. | Adverse Skin Reactions Among Healthcare Workers During the Coronavirus Disease 2019 Outbreak: A Survey in Wuhan and Its Surrounding Regions | 10.1111/bjd.19089 | Original Research | Do not present patient' underlying diseases |
| Liu C, et al. | 18F-FDG PET/CT and Serial Chest CT Findings in a COVID-19 Patient With Dynamic Clinical Characteristics in Different Period | 10.1097/RLU.0000000000003068 | Case Reports | Do not present patient' underlying diseases |
| Liu H, et al. | Management of COVID-19 in Patients After Liver Transplantation: Beijing Working Party for Liver Transplantation | 10.1007/s12072-020-10043-z | Original Research | Do not present patient' underlying diseases |
| Liu L, et al. | Optimizing screening strategies for coronavirus disease 2019: A study from Middle China | 10.1016/j.jiph.2020.05.003 | Original research | Do not present patient' underlying diseases |
| Liu L, et al. | Association Between Age and Clinical Characteristics and Outcomes of COVID-19 | 10.1183/13993003.01112-2020 | Original Article | Do not present patient' underlying diseases |
| Liu Q, et al. | Laboratory findings and a combined multifactorial approach to predict death in critically ill patients with COVID-19: a retrospective study | 10.1017/S0950268820001442 | Observational Study | Do not present patient' underlying diseases |
| Liu W, et al. | Clinical characteristics of 19 neonates born to mothers with COVID-19 | 10.1007/s11684-020-0772-y | Original Research | Do not present patient' underlying diseases |
| Loeffelholz MJ, et al. | Multicenter Evaluation of the Cepheid Xpert Xpress SARS-CoV-2 Test | 10.1128/JCM.00926-20 | Original Article | Do not present patient' underlying diseases |
| Lombardi A, et al. | Characteristics of 1573 healthcare workers who underwent nasopharyngeal swab testing for SARS-CoV-2 in Milan, Lombardy, Italy | 10.1016/j.cmi.2020.06.013 | Original research | Do not present patient' underlying diseases |
| Lu D, et al. | Asymptomatic COVID-19 Infection in Late Pregnancy Indicated No Vertical Transmission | 10.1002/jmv.25927 | Original Article | Do not present patient' underlying diseases |
| Lu L, et al. | An evidence mapping and analysis of registered COVID-19 clinical trials in China | 10.1186/s12916-020-01612-y | Original research | Do not present patient' underlying diseases |
| Lu Y, et al. | Clinical Characteristics and Radiological Features of Children Infected With the 2019 Novel Coronavirus | 10.1016/j.crad.2020.04.010 | Original Article | Do not present patient' underlying diseases |
| Lüke et al. | Coronavirus disease 2019 induces multi-lineage, morphologic changes in peripheral blood cells | 10.1002/jha2.44 | Original research | Do not present patient' underlying diseases |
| Luo Y, et al. | Investigation of COVID-19-related symptoms based on factor analysis | 10.21037/apm-20-1113 | Original research | Do not present patient' underlying diseases |
| Ma H, et al. | A Single-Center, Retrospective Study of COVID-19 Features in Children: A Descriptive Investigation | 10.1186/s12916-020-01596-9 | Original Article | Do not present patient' underlying diseases |
| Manganotti P, et al. | Miller Fisher syndrome diagnosis and treatment in a patient with SARS-CoV-2 | 10.1007/s13365-020-00858-9 | Case Reports | Do not present patient' underlying diseases |
| Mani NS, et al. | Prevalence of COVID-19 Infection and Outcomes Among Symptomatic Healthcare Workers in Seattle, Washington | 10.1093/cid/ciaa761 | Original research | Do not present patient' underlying diseases |
| Mao LJ, et al. | A Child With Household Transmitted COVID-19 | 10.1186/s12879-020-05056-w | Case Reports | Do not present patient' underlying diseases |
| Mapelli P, et al. | Dual tracer 68Ga-DOTATOC and 18F-FDG PET/computed tomography radiomics in pancreatic neuroendocrine neoplasms: an endearing tool for preoperative risk assessment | 10.1097/MNM.0000000000001236 | Original research | Do not present patient' underlying diseases |
| Masse S, et al. | Epidemiology and Clinical Symptoms Related to Seasonal Coronavirus Identified in Patients with Acute Respiratory Infections Consulting in Primary Care over Six Influenza Seasons (2014-2020) in France | 10.3390/v12060630 | Original Article | Do not present patient' underlying diseases |
| Mehta HB, et al. | Characteristics of registered clinical trials assessing treatments for COVID-19: a cross-sectional analysis | 10.1136/bmjopen-2020-039978 | Original Article | Do not present patient' underlying diseases |
| Meo SA, et al. | Novel Coronavirus 2019-nCoV: Prevalence, Biological and Clinical Characteristics Comparison With SARS-CoV and MERS-CoV | 10.26355/eurrev_202002_20379 | Original Research | Do not present patient' underlying diseases |
| Meyer B, et al. | Validation of a commercially available SARS-CoV-2 serological immunoassay | 10.1016/j.cmi.2020.06.024 | Original Research | Do not present patient' underlying diseases |
| Miller R & Englund K | Clinical Presentation and Course of COVID-19 | 10.3949/ccjm.87a.ccc013 | Brief Communication | Do not present patient' underlying diseases |
| Mohammadi A, et al. | Clinical and radiological characteristics of pediatric patients with COVID‑19: focus on imaging findings | 10.1007/s11604-020-01003-6 | Original Article | Do not present patient' underlying diseases |
| Mohr-Sasson A, et al. | Laboratory characteristics of pregnant compared to non-pregnant women infected with SARS-CoV-2 | 10.1007/s00404-020-05655-7 | Original research | Do not present patient' underlying diseases |
| Mondejar-Lopez et al. | Impact of SARS-CoV-2 infection in patients with cystic fibrosis in Spain: Incidence and results of the national CF-COVID19-Spain survey | 10.1016/j.rmed.2020.106062 | Original research | Do not present patient' underlying diseases |
| Moro E, et al. | The international European Academy of Neurology survey on neurological symptoms in patients with COVID-19 infection | 10.1111/ene.14407 | Original research | Do not present patient' underlying diseases |
| Nasir UM, et al. | The Role of Emergency Radiology in COVID-19: From Preparedness to Diagnosis | 10.1177/0846537120916419 | Original research | Do not present patient' underlying diseases |
| Nguyen HT, et al. | Fear of COVID-19 Scale—Associations of Its Scoreswith Health Literacy and Health-Related Behaviorsamong Medical Students | 10.3390/ijerph17114164 | Original research | Do not present patient' underlying diseases |
| Nie S, et al. | Coronavirus Disease 2019-related dyspnea cases difficult to interpret using chest computed tomography | 10.1016/j.rmed.2020.105951 | Case Reports | Do not present patient' underlying diseases |
| Pan Y, et al. | Epidemiological and Clinical Characteristics of 26 Asymptomatic Severe Acute Respiratory Syndrome Coronavirus 2 Carriers | 10.1093/infdis/jiaa205 | Original Article | Do not present patient' underlying diseases |
| Pertile D, et al. | The impact of COVID-19 pandemic on surgical residency programmes in Italy: a nationwide analysis on behalf of the Italian Polyspecialistic Young Surgeons Society (SPIGC) | 10.1007/s13304-020-00811-9 | Original research | Do not present patient' underlying diseases |
| Petrescu AM, et al. | Electroencephalogram (EEG) in COVID-19: A systematic retrospective study. | 10.1016/j.neucli.2020.06.001 | Original Research | Do not present patient' underlying diseases |
| Powell-Jackson T, et al. | Infection Prevention and Control Compliance in Tanzanian Outpatient Facilities: A Cross-Sectional Study With Implications for the Control of COVID-19 | 10.1016/S2214-109X(20)30222-9 | Original Research | Do not present patient' underlying diseases |
| Pung R, et al. | Investigation of three clusters of COVID-19 in Singapore: implications for surveillance and response measures | 10.1016/S0140-6736(20)30528-6 | Original research | Do not present patient' underlying diseases |
| Qiao XM, et al. | Re-positive Cases of Nucleic Acid Tests in Discharged Patients With COVID-19: A Follow-Up Study. | 10.3389/fmed.2020.00349 | Original Research | Do not present patient' underlying diseases |
| Raciborski F, et al. | Dynamics of the coronavirus disease 2019 outbreak in Poland: an epidemiological analysis of the first 2 months of the epidemic | 10.20452/pamw.15430 | Original research | Do not present patient' underlying diseases |
| Ratnarathon AC, et al. | Potential dual dengue and SARS-CoV-2 infection in Thailand: A case study | 10.1016/j.heliyon.2020.e04175 | Case Reports | Do not present patient' underlying diseases |
| Roland LT, et al. | Smell and taste symptom-based predictive model for COVID-19 diagnosis | 10.1002/alr.22602 | Original research | Do not present patient' underlying diseases |
| Rosenberg ES, et al. | Cumulative incidence and diagnosis of SARS-CoV-2 infection in New York | 10.1016/j.annepidem.2020.06.004 | Original Research | Do not present patient' underlying diseases |
| Safavi F, et al. | B-cell Depleting Therapies May Affect Susceptibility to Acute Respiratory Illness Among Patients With Multiple Sclerosis During the Early COVID-19 Epidemic in Iran | 10.1016/j.msard.2020.102195 | Original Research | Do not present patient' underlying diseases |
| Shabto JM, et al. | Characteristics and outcomes of COVID-19 positive patients with diabetes managed as outpatients | 10.1016/j.diabres.2020.108229 | Original Research | Do not present patient' underlying diseases |
| Shams SA, et al. | Analyzing COVID-19 pandemic for unequal distribution of tests, identified cases, deaths, and fatality rates in the top 18 countries | 10.1016/j.dsx.2020.06.051 | Original Research | Do not present patient' underlying diseases |
| Sharmeen S, et al. | COVID-19 in rheumatic disease patients on immunosuppressive agents | 10.1016/j.semarthrit.2020.05.010 | Original research | Do not present patient' underlying diseases |
| Sheikh JA, et al. | Emerging Genetic Diversity Among Clinical Isolates of SARS-CoV-2: Lessons for Today | 10.1016/j.meegid.2020.104330 | Short communication | Do not present patient' underlying diseases |
| Shen N, et al. | Characteristics and diagnosis rate of 5630 subjects receiving SARS-CoV-2 nucleic acid tests from Wuhan, China | 10.1172/jci.insight.137662 | Original Article | Do not present patient' underlying diseases |
| Shen Q, et al. | Novel coronavirus infection in children outside of Wuhan, China | 10.1002/ppul.24762 | Original research | Do not present patient' underlying diseases |
| Song J, et al. | End-to-end automatic differentiation of the coronavirus disease 2019 (COVID-19) from viral pneumonia based on chest CT | 10.1007/s00259-020-04929-1 | Original research | Do not present patient' underlying diseases |
| Su L, et al. | The Different Clinical Characteristics of Corona Virus Disease Cases Between Children and Their Families in China - The Character of Children With COVID-19 | 10.1080/22221751.2020.1744483 | Original research | Do not present patient' underlying diseases |
| Sun JK, et al. | Serum calcium as a biomarker of clinical severity and prognosis in patients with coronavirus disease 2019 | 10.18632/aging.103526 | Original research | Do not present patient' underlying diseases |
| Suso-Ribera C, et al. | How Much Support Is There for the Recommendations Made to the General Population during Confinement? A Study during the First Three Days of the COVID-19 Quarantine in Spain | 10.3390/ijerph17124382 | Original research | Do not present patient' underlying diseases |
| Suwanwongse K & Shabarek N | Fatal Outcome in a Kidney-Pancreas Transplant Recipient With COVID-19 | 10.7759/cureus.8691 | Case Reports | Do not present patient' underlying diseases |
| Takahashi N, et al. | Clinical course of a critically ill patient with severe acute respiratory syndrome coronavirus 2 (SARS-CoV-2) | 10.1007/s10047-020-01183-y | Case report | Do not present patient' underlying diseases |
| Tan YP, et al. | Epidemiologic and clinical characteristics of 10 children with coronavirus disease 2019 in Changsha, China | 10.1016/j.jcv.2020.104353 | Original research | Do not present patient' underlying diseases |
| Thai PQ, et al. | Factors associated with the duration of hospitalisation among COVID-19 patients in Vietnam: A survival analysis | 10.1017/S0950268820001259 | Original Article | Do not present patient' underlying diseases |
| Tian H, et al. | Case Report: Clinical Treatment of the First Critical Patient With Coronavirus Disease (COVID-19) in Liaocheng, Shandong Province | 10.3389/fmed.2020.00249 | Case Report | Do not present patient' underlying diseases |
| Tian S, et al. | Characteristics of COVID-19 Infection in Beijing | 10.1016/j.jinf.2020.02.018 | Original Research | Do not present patient' underlying diseases |
| Toubiana J, et al. | Kawasaki-like multisystem inflammatory syndrome in children during the covid-19 pandemic in Paris, France: prospective observational study | 10.1136/bmj.m2094 | Original research | Do not present patient' underlying diseases |
| Van Houtven CH, et al. | Essential Long-Term Care Workers Commonly Hold Second Jobs and Double- Or Triple-Duty Caregiving Roles | 10.1111/jgs.16509 | Brief Report | Do not present patient' underlying diseases |
| Viale G, et al. | Benefit Ratio Adaptation of Breast Cancer Care at the Epicenter of COVID-19 Outbreak | 10.1634/theoncologist.2020-0316 | Original research | Do not present patient' underlying diseases |
| Wang B, et al. | Implications of the lack of a unified research project framework: an investigation into the registration of clinical trials of COVID-19 | 10.1080/03007995.2020.1771294 | Original research | Do not present patient' underlying diseases |
| Wang C, et al. | A Longitudinal Study on the Mental Health of General Population During the COVID-19 Epidemic in China | 10.1016/j.bbi.2020.04.028 | Original Research | Do not present patient' underlying diseases |
| Wang H, et al. | Characteristic CT Findings Distinguishing 2019 Novel Coronavirus Disease (COVID-19) From Influenza Pneumonia | 10.1007/s00330-020-06880-z | Original Article | Do not present patient' underlying diseases |
| Wang J, et al. | CT characteristics of patients infected with 2019 novel coronavirus: association with clinical type | 10.1016/j.crad.2020.04.001 | Original research | Do not present patient' underlying diseases |
| Wang L, et al. | Quadruple Therapy for Asymptomatic COVID-19 Infection Patients | 10.1080/14787210.2020.1758066 | Original Research | Do not present patient' underlying diseases |
| Wei Y, et al. | Analysis of 2019 novel coronavirus infection and clinical characteristics of outpatients: An epidemiological study from a fever clinic in Wuhan, China | 10.1002/jmv.26175 | Original research | Do not present patient' underlying diseases |
| Wood et al. | Outcomes of a Rapid Adolescent Telehealth Scale-Up During the COVID-19 Pandemic | 10.1016/j.jadohealth.2020.05.025 | Original research | Do not present patient' underlying diseases |
| Wu C, et al. | Clinical Manifestation and Laboratory Characteristics of SARS-CoV-2 Infection in Pregnant Women | 10.1007/s12250-020-00227-0 | Research Article | Do not present patient' underlying diseases |
| Wu H, et al. | Clinical and Immune Features of Hospitalized Pediatric Patients With Coronavirus Disease 2019 (COVID-19) in Wuhan, China | 10.1001/jamanetworkopen.2020.10895 | Original research | Do not present patient' underlying diseases |
| Wu J, et al. | Identification of RT-PCR-Negative Asymptomatic COVID-19 Patients via Serological Testing | 10.3389/fpubh.2020.00267 | Case Reports | Do not present patient' underlying diseases |
| Wu P et al. | Characteristics of Ocular Findings of Patients With Coronavirus Disease 2019 (COVID-19) in Hubei Province, China | 10.1001/jamaophthalmol.2020.1291 | Original research | Do not present patient' underlying diseases |
| Wu Y, et al. | Relationship between ABO blood group distribution and clinical characteristics in patients with COVID-19 | 10.1016/j.cca.2020.06.026 | Original research | Do not present patient' underlying diseases |
| Xia W, et al. | Clinical and CT Features in Pediatric Patients With COVID-19 Infection: Different Points From Adults | 10.1002/ppul.24718 | Original Research | Do not present patient' underlying diseases |
| Xiao AT, et al. | Dynamic profile of RT-PCR findings from 301 COVID-19 patients in Wuhan, China | 10.1016/j.jcv.2020.104346 | Original research | Do not present patient' underlying diseases |
| Xiao M, et al. | Multiple approaches for massively parallel sequencing of SARS-CoV-2 genomes directly from clinical samples | 10.1186/s13073-020-00751-4 | Original Research | Do not present patient' underlying diseases |
| Xie C, et al. | Comparison of Different Samples for 2019 Novel Coronavirus Detection by Nucleic Acid Amplification Tests | 10.1016/j.ijid.2020.02.050 | Original Research | Do not present patient' underlying diseases |
| Xu et al. | A Deep Learning System to Screen Novel Coronavirus Disease 2019 Pneumonia | 10.1016/j.eng.2020.04.010 | Original research | Do not present patient' underlying diseases |
| Xu Y, et al. | Clinical and Computed Tomographic Imaging Features of Novel Coronavirus Pneumonia Caused by SARS-CoV-2 | 10.1016/j.jinf.2020.02.017 | Original Research | Do not present patient' underlying diseases |
| Xu Y, et al. | Characteristics of pediatric SARS-CoV-2 infection and potential evidence for persistent fecal viral shedding | 10.1038/s41591-020-0817-4 | Original research | Do not present patient' underlying diseases |
| Yan J, et al. | Coronavirus Disease 2019 in Pregnant Women: A Report Based on 116 Cases | 10.1016/j.ajog.2020.04.014 | Original Article | Do not present patient' underlying diseases |
| Ye G, et al. | Clinical characteristics of severe acute respiratory syndrome coronavirus 2 reactivation. | 10.1016/j.jinf.2020.03.001 | Research Article | Do not present patient' underlying diseases |
| Yin S, et al. | The Implications of Preliminary Screening and Diagnosis: Clinical Characteristics of 33 Mild Patients With SARS-CoV-2 Infection in Hunan, China | 10.1016/j.jcv.2020.104397 | Original Research | Do not present patient' underlying diseases |
| Yuan B, et al. | Correlation between immune response and self-reported depression during convalescence from COVID-19 | 10.1016/j.bbi.2020.05.062 | Original research | Do not present patient' underlying diseases |
| Yue H, et al. | The epidemiology and clinical characteristics of co-infection of SARS-CoV-2 and influenza viruses in patients during COVID-19 outbreak | 10.1002/jmv.26163 | Original Article | Do not present patient' underlying diseases |
| Zhang & Huang | Clinical Features of 33 Cases in Children Infected With SARS-CoV-2 in Anhui Province, China-A Multi-Center Retrospective Cohort Study | 10.3389/fpubh.2020.00255 | Original Research | Do not present patient' underlying diseases |
| Zhang G, et al. | Analysis of clinical characteristics and laboratory findings of 95 cases of 2019 novel coronavirus pneumonia in Wuhan, China: a retrospective analysis | 10.1186/s12931-020-01338-8 | Original Research | Do not present patient' underlying diseases |
| Zhang H, et al. | Clinical Characteristics of 194 Cases of COVID-19 in Huanggang and Taian, China | 10.1007/s15010-020-01440-5 | Original Article | Do not present patient' underlying diseases |
| Zhang S, et al. | High-resolution CT Features of 17 Cases of Corona Virus Disease 2019 in Sichuan Province, China | 10.1183/13993003.00334-2020 | Original Research | Do not present patient' underlying diseases |
| Zhang T, et al. | Detectable SARS-CoV-2 Viral RNA in Feces of Three Children During Recovery Period of COVID-19 Pneumonia | 10.1002/jmv.25795 | Original Research | Do not present patient' underlying diseases |
| Zhao W, et al. | Characteristics of Children With Reactivation of SARS-CoV-2 Infection After Hospital Discharge | 10.1177/0009922820928057 | Original research | Do not present patient' underlying diseases |
| Zheng G, et al. | Clinical characteristics of acute respiratory syndrome with SARS-CoV-2 infection in children in SouthChina | 10.1002/ppul.24921 | Original research | Do not present patient' underlying diseases |
| Zheng T, et al. | Clinical characteristics and outcomes of COVID-19 patients with gastrointestinal symptoms admitted to Jianghan Fangcang Shelter Hospital in Wuhan, China | 10.1002/jmv.26146 | Original Article | Do not present patient' underlying diseases |
| Zheng Y, et al. | The hemocyte counts as a potential biomarker for predicting disease progression in COVID-19: a retrospective study | 10.1515/cclm-2020-0377 | Original Article | Do not present patient' underlying diseases |
| Zhong ZF, et al. | Epidemiological and clinical characteristics of COVID-19 patients in Hengyang, Hunan Province, China | 10.12998/wjcc.v8.i12.2554 | Original Research | Do not present patient' underlying diseases |
| Zhou B, et al. | The Duration of Viral Shedding of Discharged Patients With Severe COVID-19 | 10.1093/cid/ciaa451 | Original Research | Do not present patient' underlying diseases |
| Zhou C, et al. | Predictive factors of severe coronavirus disease 2019 in previously healthy young adults: a single-center, retrospective study | 10.1186/s12931-020-01412-1 | Original research | Do not present patient' underlying diseases |
| Zhou H et al. | Coronavirus disease 2019 (COVID-19): chest CT characteristics benefit to early disease recognition and patient classification-a single center experience | 10.21037/atm-20-2119a | Original research | Do not present patient' underlying diseases |
| Zhou Y, et al. | Cohort study of chest CT and clinical changes in 29 patients with coronavirus disease 2019 (COVID-19) | 10.1007/s00330-020-07007-0 | Original research | Do not present patient' underlying diseases |
| Zhou Y, et al. | Risk Factors Associated With Disease Progression in a Cohort of Patients Infected With the 2019 Novel Coronavirus | 10.21037/apm.2020.03.26 | Original Research | Do not present patient' underlying diseases |
| Zhu L, et al. | Clinical characteristics of a case series of children with coronavirus disease 2019 | 10.1002/ppul.24767 | Original research | Do not present patient' underlying diseases |
| Zhu L, et al. | Successful Recovery of COVID-19 Pneumonia in a Renal Transplant Recipient With Long-Term Immunosuppression | 10.1111/ajt.15869 | Original Research | Do not present patient' underlying diseases |
| Zhuang SF, et al. | Low-grade fever during COVID-19 convalescence: A report of 3 cases | 10.12998/wjcc.v8.i12.2655 | Case Reports | Do not present patient' underlying diseases |
| Zu ZY, et al. | Coronavirus Disease 2019 (COVID-19): A Perspective From China | 10.1148/radiol.2020200490 | Original Research | Do not present patient' underlying diseases |
| Chen X, et al. | Dynamic Chest CT Evaluation in Three Cases of 2019 Novel Coronavirus Pneumonia. | 10.34172/aim.2020.11 | Original research | Full text not available |
| Bai K, et al. | Clinical Analysis of 25 Novel Coronavirus Infections in Children | 10.1097/INF.0000000000002740 | Original research | Full text not available |
| Bryant JE, et al. | Serology for SARS-CoV-2: Apprehensions, opportunities, and the path forward | 10.1126/sciimmunol.abc6347 | Original research | Full text not available |
| Du W, et al. | Clinical Characteristics of COVID-19 in Children Compared With Adults in Shandong Province, China | 10.1007/s15010-020-01427-2 | Original research | Full text not available |
| Han C, et al. | Digestive Symptoms in COVID-19 Patients With Mild Disease Severity: Clinical Presentation, Stool Viral RNA Testing, and Outcomes | 10.14309/ajg.0000000000000664 | Original research | Full text not available |
| Lu Y, et al. | Symptomatic Infection Is Associated With Prolonged Duration of Viral Shedding in Mild Coronavirus Disease 2019: A Retrospective Study of 110 Children in Wuhan | 10.1097/INF.0000000000002729 | Original research | Full text not available |
| Mi B, et al. | Characteristics and Early Prognosis of COVID-19 Infection in Fracture Patients | 10.2106/JBJS.20.00390 | Original research | Full text not available |
| Mishra V, et al. | COVID-19-Hospitalized Patients in Karnataka: Survival and Stay Characteristics | 10.4103/ijph.IJPH_486_20 | Original research | Full text not available |
| Peng L, et al. | Improved Early Recognition of Coronavirus Disease-2019 (COVID-19): Single-Center Data From a Shanghai Screening Hospital | 10.34172/aim.2020.10 | Original research | Full text not available |
| Qian G, et al. | Epidemiologic and Clinical Characteristics of 91 Hospitalized Patients With COVID-19 in Zhejiang, China: A Retrospective, Multi-Centre Case Series | 10.1093/qjmed/hcaa089 | Original research | Full text not available |
| Qiu L, et al. | A Typical Case of Critically Ill Infant of Coronavirus Disease 2019 With Persistent Reduction of T Lymphocytes | 10.1097/INF.0000000000002720 | Original research | Full text not available |
| Yang P, et al. | Clinical Characteristics and Risk Assessment of Newborns Born to Mothers With COVID-19 | 10.1016/j.jcv.2020.104356 | Original research | Full text not available |
| Zheng Y, et al. | Epidemiological Characteristics and Clinical Features of 32 Critical and 67 Noncritical Cases of COVID-19 in Chengdu | 10.1016/j.jcv.2020.104366 | Original research | Full text not available |
| - | Expert consensus for bronchoscopy during the epidemic of 2019 novel coronavirus infection (Trial version) | 10.3760/cma.j.issn.1001-0939.2020.03.012 | Original research | Full text available only in Chinese |
| - | An update on the epidemiological characteristics of novel coronavirus pneumonia (COVID-19) | 10.3760/cma.j.issn.0254-6450.2020.02.002 | Original research | Full text available only in Chinese |
| Bai SL, et al. | Analysis of the first cluster of cases in a family of novel coronavirus pneumonia in Gansu Province | 10.3760/cma.j.issn.0253-9624.2020.0005 | Original research | Full text available only in Chinese |
| Cao L, et al. | A preliminary investigation on the serological and epidemiological characteristics of severe acute respiratory syndrome in children | PMID: 15631713 | Original research | Full text available only in Chinese |
| Chen L, et al. | Analysis of clinical features of 29 patients with 2019 novel coronavirus pneumonia | 10.3760/cma.j.issn.1001-0939.2020.0005 | Original research | Full text available only in Chinese |
| Chen S, et al. | Pregnant women with new coronavirus infection: a clinical characteristics and placental pathological analysis of three cases | 10.3760/cma.j.cn112151-20200225-00138 | Original research | Full text available only in Chinese |
| Chen XB, et al. | Retrospective Analysis of 61 Cases of Children Died of Viral Pneumonia | 10.12116/j.issn.1004-5619.2020.02.002 | Original research | Full text available only in Chinese |
| Cucchiari D, et al. | Pneumococcal superinfection in COVID-19 patients: A series of 5 cases | 10.1016/j.medcli.2020.05.022 | Case Reports | Full text available only in Spanish |
| Dong XC, et al. | Epidemiological Characteristics of Confirmed COVID-19 Cases in Tianjin | 10.3760/cma.j.cn112338-20200221-00146 | Original research | Full text available only in Chinese |
| Feng K, et al. | Analysis of CT features of 15 Children with 2019 novel coronavirus infection | 10.3760/cma.j.issn.0578-1310.2020.0007 | Original research | Full text available only in Chinese |
| Fuentes B, et al. | Impact of the COVID-19 pandemic on the organisation of stroke care. | 10.1016/j.nrl.2020.05.007 | Original research | Full text available only in Spanish |
| Gao ZC | Efficient management of novel coronavirus pneumonia by efficient prevention and control in scientific manner | 10.3760/cma.issn.1001-0939.2020.03.002 | Original research | Full text available only in Chinese |
| Gong et al. | Experimental study and reflection on peacetime and wartime reconstruction of large general hospitals in public health emergencies | 10.11817/j.issn.1672-7347.2020.200401 | Original research | Full text available only in Chinese |
| Gou FX, et al. | Epidemiological Characteristics of COVID-19 in Gansu Province | 10.3760/cma.j.cn112338-20200229-00216 | Original research | Full text available only in Chinese |
| Gu M, et al. | Analysis of property and efficacy of traditional Chinese medicine in staging revention and treatment of coronavirus disease 2019 | 10.19540/j.cnki.cjcmm.20200225.501 | Original research | Full text available only in Chinese |
| Guan Q, et al. | Epidemiological investigation of a family clustering of COVID-19 | 10.3760/cma.j.cn112338-20200223-00152 | Original research | Full text available only in Chinese |
| He XW et al. | Impact of complicated myocardial injury on the clinical outcome of severe or critically ill COVID-19 patients | 10.3760/cma.j.cn112148-20200228-00137 | Original research | Full text available only in Chinese |
| Hu ZB & Ci C | Screening and management of asymptomatic infection of coronavirus disease 2019 (COVID-19) | 10.3760/cma.j.cn112150-20200229-00220 | Original research | Full text available only in Chinese |
| Jiang et al. | Psychological status of the staff in a general hospital during the outbreak of coronavirus disease 2019 and its influential factors | 10.11817/j.issn.1672-7347.2020.200190 | Original research | Full text available only in Chinese |
| Lara Álvarez MÁ, et al. | COVID-19 mortality in cancer patients in a Madrid hospital during the first 3 weeks of the epidemic | 10.1016/j.medcli.2020.05.005 | Original research | Full text available only in Spanish |
| Li CX, et al. | Clinical Study and CT Findings of a Familial Cluster of Pneumonia with Coronavirus Disease 2019 (COVID-19) | 10.12182/20200360107 | Original research | Full text available only in Chinese |
| Li et al. | Clinical characteristics, diagnosis and treatment strategies | 10.11817/j.issn.1672-7347.2020.200264 | Original research | Full text available only in Chinese |
| Li XQ, et al. | Comparison of Epidemic Characteristics Between SARS in 2003 and COVID-19 in 2020 in Guangzhou | 10.3760/cma.j.cn112338-20200228-00209 | Original research | Full text available only in Chinese |
| Li YY, et al. | Comparison of the clinical characteristics between RNA positive and negative patients clinically diagnosed with 2019 novel coronavirus pneumonia | 10.3760/cma.j.cn112147-20200214-00095 | Original research | Full text available only in Chinese |
| Liu BL, et al. | Health management of breast cancer patients outside the hospital during the outbreak of 2019 novel coronavirus disease | 10.3760/cma.j.cn112152-20200221-00110 | Original research | Full text available only in Chinese |
| Liu C, et al. | Preliminary study of the relationship between novel coronavirus pneumonia and liver function damage: a multicenter study | 10.3760/cma.j.issn.1007-3418.2020.02.003 | Original research | Full text available only in Chinese |
| Liu et al. | Relation between blood glucose and the prognosis of severe coronavirus disease 2019 | 10.11817/j.issn.1672-7347.2020.200182 | Original research | Full text available only in Chinese |
| Liu M, et al. | Clinical characteristics of 30 medical workers infected with new coronavirus pneumonia | 10.3760/cma.j.issn.1001-0939.2020.03.014 | Original research | Full text available only in Chinese |
| Liu RR, et al. | CT imaging analysis of 33 cases with the 2019 novel coronavirus infection | 10.3760/cma.j.cn112137-20200203-00182 | Original research | Full text available only in Chinese |
| López-Bravo A, et al. | Impact of the COVID-19 pandemic on headache management in Spain: an analysis of the current situation and future perspectives | 10.1016/j.nrl.2020.05.006 | Original research | Full text available only in Spanish |
| Ma FH, et al. | Surgical treatment strategy for digestive system malignancies during the outbreak of novel coronavirus pneumonia | 10.3760/cma.j.cn112152-20200223-00117 | Original research | Full text available only in Chinese |
| Mao DM, et al. | Guide to the Forensic Pathology Practice on Death Cases Related to Corona Virus Disease 2019 (COVID-19) | 10.12116/j.issn.1004-5619.2020.01.003 | Original research | Full text available only in Chinese |
| Mei H & Hu Y | Characteristics, causes, diagnosis and treatment of coagulation dysfunction in patients with COVID-19 | 10.3760/cma.j.issn.0253-2727.2020.0002 | Original research | Full text available only in Chinese |
| Peng YD, et al. | Clinical characteristics and outcomes of 112 cardiovascular disease patients infected by 2019-nCoV | 10.3760/cma.j.cn112148-20200220-00105 | Original research | Full text available only in Chinese |
| Qian ZP, et al. | Analysis of baseline liver biochemical parameters in 324 cases with novel coronavirus pneumonia in Shanghai area | 10.3760/cma.j.cn501113-20200229-00076 | Original research | Full text available only in Chinese |
| Ren YH, et al. | When COVID-19 encounters interstitial lung disease: challenges and management | 10.3760/cma.j.cn112147-20200315-00339 | Original research | Full text available only in Chinese |
| Rodríguez A, et al. | Severe infection due to the SARS-CoV-2 coronavirus: Experience of a tertiary hospital with COVID-19 patients during the 2020 pandemic | 10.1016/j.medin.2020.05.018 | Original research | Full text available only in Spanish |
| Shi Y, et al. | Progress and challenge of vaccine development against 2019 novel coronavirus (2019-nCoV) | 10.3760/cma.j.cn112150-20200317-00366 | Original research | Full text available only in Chinese |
| Sun C, et al. | Clinical analysis of 150 cases of 2019 novel coronavirus infection in Nanyang City, Henan Province | 10.3760/cma.j.cn112147-20200224-00168 | Original research | Full text available only in Chinese |
| Wang et al. | Clinical characteristics and the risk factors for severe events of elderly coronavirus disease 2019 patients | 10.11817/j.issn.1672-7347.2020.200292 | Original research | Full text available only in Chinese |
| Wang J, et al. | Dynamic changes of chest CT imaging in patients with corona virus disease-19 (COVID-19) | PMID: 32096366 | Original research | Full text available only in Chinese |
| Wang XF, et al. | Clinical and epidemiological characteristics of 34 children with 2019 novel coronavirus infection in Shenzhen | 10.3760/cma.j.issn.0578-1310.2020.0008 | Original research - Retracted | Full text available only in Chinese |
| Wen et al. | Clinical characteristics of coronavirus disease 2019 patients complicated with liver injury | 10.11817/j.issn.1672-7347.2020.200225 | Original research | Full text available only in Chinese |
| Wu J, et al. | Novel coronavirus pneumonia (COVID-19) CT distribution and sign features | 10.3760/cma.j.cn112147-20200217-00106 | Original research | Full text available only in Chinese |
| Wu WS, et al. | Investigation and Analysis on Characteristics of a Cluster of COVID-19 Associated With Exposure in a Department Store in Tianjin | 10.3760/cma.j.cn112338-20200221-00139 | Original research | Full text available only in Chinese |
| Yang HY, et al. | The preliminary analysis on the characteristics of the cluster for the Corona Virus Disease | 10.3760/cma.j.cn112338-20200223-00153 | Original research | Full text available only in Chinese |
| Yao N, et al. | Clinical characteristics and influencing factors of patients with novel coronavirus pneumonia combined with liver injury in Shaanxi region | 10.3760/cma.j.cn501113-20200226-00070 | Original research | Full text available only in Chinese |
| Yao XH, et al. | A pathological report of three COVID-19 cases by minimally invasive autopsies | 10.3760/cma.j.cn112151-20200312-00193 | Original research | Full text available only in Chinese |
| Zhang H, et al. | Lowering the intraocular pressure by a combination of timolol with adrenergic agents | 10.1016/j.eururo.2020.03.030 | Original research | Full text available only in German |
| Zhang J, et al. | Management strategies for patients with gynecological malignancies during the outbreak of COVID19 | 10.3760/cma.j.cn112141-20200302-00168 | Original research | Full text available only in Chinese |
| Zhang MQ, et al. | Clinical features of 2019 novel coronavirus pneumonia in the early stage from a fever clinic in Beijing | 10.3760/cma.j.issn.1001-0939.2020.03.015 | Original research | Full text available only in Chinese |
| Zhang T, et al. | Comparison of clinical and pathological features between severe acute respiratory syndrome and coronavirus disease 2019 | 10.3760/cma.j.cn112147-20200311-00312 | Original research | Full text available only in Chinese |
| Zhang Y, et al. | Clinical and Coagulation Characteristics of 7 Patients With Critical COVID-2019 Pneumonia and Acro-Ischemia | 10.3760/cma.j.issn.0253-2727.2020.0006 | Original research | Full text available only in Chinese |
| Zhao L, et al. | The treatment proposal for the patients with breast diseases in the central epidemic area of 2019 coronavirus disease | 10.3760/cma.j.cn112139-20200221-00116 | Original research | Full text available only in Chinese |
| Zhong Q, et al. | CT imaging features of patients with different clinical types of coronavirus disease 2019 (COVID-19) | PMID: 32207591 | Original research | Full text available only in Chinese |
| Zhonghua et al. | The epidemiological characteristics of an outbreak of 2019 novel coronavirus diseases (COVID-19) in China | 10.3760/cma.j.issn.0254-6450.2020.02.003 | Original research | Full text available only in Chinese |
| Zhou et al. | Clinical characteristics of 16 patients with fecal severe acute respiratory syndrome coronavirus 2 nucleic acid-positive | 10.11817/j.issn.1672-7347.2020.200230 | Original research | Full text available only in Chinese |
| Zhou L, et al. | Early detection and disease assessment of patients with novel coronavirus pneumonia | 10.3760/cma.j.issn.1001-0939.2020.03.003 | Original research | Full text available only in Chinese |
| Zhou L, et al. | Cause analysis and treatment strategies of "recurrence" with novel coronavirus pneumonia (covid-19) patients after discharge from hospital | 10.3760/cma.j.cn112147-20200229-00219 | Original research | Full text available only in Chinese |
| Zhu ZW, et al. | Comparison of heart failure and 2019 novel coronavirus pneumonia in chest CT features and clinical characteristics | 10.3760/cma.j.cn112148-20200218-00093 | Original research | Full text available only in Chinese |
| - | Early Epidemiological and Clinical Characteristics of 28 Cases of Coronavirus Disease in South Korea | 10.24171/j.phrp.2020.11.1.03 | CDC Report |  |
| Aaby P, et al. | The non-specific and sex-differential effects of vaccines | 10.1038/s41577-020-0338-x | Review |  |
| Aardema F. | COVID-19, obsessive-compulsive disorder and invisible life forms that threaten the self | 10.1016/j.jocrd.2020.100558 | Short communication |  |
| Adhikari SP, et al. | Epidemiology, causes, clinical manifestation and diagnosis, prevention and control of coronavirus disease (COVID-19) during the early outbreak period: a scoping review. | 10.1186/s40249-020-00646-x | Review |  |
| Alanagreh L, et al. | The Human Coronavirus Disease COVID-19: Its Origin, Characteristics, and Insights into Potential Drugs and Its Mechanisms. | 10.3390/pathogens9050331 | Review |  |
| Aledo-Serrano Á, et al. | Genetic epilepsies and COVID-19 pandemic: Lessons from the caregiver perspective | 10.1111/epi.16537 | Letter |  |
| Allam M, et al. | COVID-19 Diagnostics, Tools, and Prevention | 10.3390/diagnostics10060409 | Review |  |
| Alramthan & Aldaraji | A Case of COVID-19 Presenting in Clinical Picture Resembling Chilblains Disease. First Report From the Middle East | 10.1111/ced.14243 | Correspondence |  |
| Altuntas Aydin O, et al. | HIV/SARS‐CoV‐2 co‐infected patients in Istanbul, Turkey | 10.1002/jmv.25955 | Letter to the editor |  |
| Aly MH, et al. | Indicators of Critical Illness and Predictors of Mortality in COVID-19 Patients | 10.2147/IDR.S261159 | Review |  |
| Ambrosi P, et al. | Epidemiological and clinical characteristics of heart transplant recipients during the 2019 coronavirus outbreak in Wuhan, China | 10.1016/j.healun.2020.04.002 | Comment |  |
| Andrenelli E, et al. | International Multiprofessional Steering Committee of Cochrane Rehabilitation REH-COVER action. Systematic rapid living review on rehabilitation needs due to COVID-19 | 10.23736/S1973-9087.20.06435-7 | Review |  |
| Antwi-Amoabeng D, et al. | Clinical outcomes in COVID-19 patients treated with tocilizumab: An individual patient data systematic review | 10.1002/jmv.26038 | Review |  |
| Balachandar V, et al. | Follow-up studies in COVID-19 recovered patients - is it mandatory? | 10.1016/j.scitotenv.2020.139021 | Review |  |
| Baldotto C, et al. | Lung Cancer and the COVID-19 pandemic: Recommendations from the Brazilian Thoracic Oncology Group | 10.6061/clinics/2020/e2060 | Review |  |
| Bao J, et al. | Comparative analysis of laboratory indexes of severe and non-severe patients infected with COVID-19 | 10.1016/j.cca.2020.06.009 | Review |  |
| Barzegar M, et al. | Characteristics of COVID-19 disease in multiple sclerosis patients | 10.1016/j.msard.2020.102276 | Correspondence |  |
| Bhanushali P, et al. | COVID-19: Changing Trends and Its Impact on Future of Dentistry | 10.1155/2020/8817424 | Review |  |
| Blasco ML, et al. | Co-detection of Respiratory Pathogens in Patients Hospitalized With Coronavirus Viral disease-2019 Pneumonia | 10.1002/jmv.25922 | Letter to the editor |  |
| Bolay H, et al. | COVID‐19 is a Real Headache! | 10.1111/head.13856 | Views and Perspectives |  |
| Bulut Ö & GÜrsel İ | Mesenchymal stem cell derived extracellular vesicles: promising immunomodulators against autoimmune, autoinflammatory disorders and SARS-CoV-2 infection | 10.3906/biy-2002-79 | Review |  |
| Cai Q & Chen J | Reply to "Clinical Characteristics of COVID-19 Patients With Abnormal Liver Tests" | 10.1016/j.jhep.2020.04.042 | Letter to the editor |  |
| Cao Q, et al. | SARS-CoV-2 infection in children: Transmission dynamics and clinical characteristics | 10.1016/j.jfma.2020.02.009 | Comment |  |
| Carneiro A, et al. | Impact of the COVID-19 Pandemic on the Urologist's Clinical Practice in Brazil: A Management Guideline Proposal for Low- And Middle-Income Countries During the Crisis Period | 10.1590/S1677-5538.IBJU.2020.04.03 | Letter to the editor |  |
| Cevik M, et al. | COVID-19 pandemic - A focused review for clinicians. | 10.1016/j.cmi.2020.04.023 | Review |  |
| Chakraborty S & Basu A | The COVID-19 pandemic: catching up with the cataclysm | 10.12688/f1000research.24963.1 | Review |  |
| Chambers CD, et al. | Evaluation of SARS-CoV-2 in Breastmilk from 18 Infected Women | 10.1101/2020.06.12.20127944 | Preprint |  |
| Chang TH, et al. | Clinical characteristics and diagnostic challenges of pediatric COVID-19: A systematic review and meta-analysis. | 10.1016/j.jfma.2020.04.007 | Systematic Review |  |
| Chen ATC, et al. | Clinical Characteristics of Covid-19 in China | 10.1056/NEJMc2005203#sa2 | Comment |  |
| Chen F, et al. | Clinical characteristics and risk factors for mortality among inpatients with COVID-19 in Wuhan, China | 10.1002/ctm2.40 | Letter to the Editor |  |
| Chen H, et al. | Clinical and imaging features of COVID-19 | 10.1016/j.jrid.2020.04.003 | Review |  |
| Chen J, et al. | COVID-19 infection: the China and Italy perspectives | 10.1038/s41419-020-2603-0 | Review |  |
| Chen L, et al. | Clinical Characteristics of Pregnant Women with Covid-19 in Wuhan, China | 10.1056/NEJMc2009226 | Correspondence |  |
| Chen P & Zhou B | Clinical Characteristics of COVID-19 in Patients With Liver Injury | 10.1016/j.cgh.2020.04.043 | Letter to the editor |  |
| Chen P, et al. | Clinical characteristics of COVID-19 patients with abnormal liver tests | 10.1016/j.jhep.2020.04.028 | Letter to the editor |  |
| Chen S, et al. | Fangcang shelter hospitals: a novel concept for responding to public health emergencies. | 10.1016/S0140-6736(20)30744-3 | Review |  |
| Cheng F, et al. | COVID-19 treatment: Combining anti-inflammatory and antiviral therapeutics using a network-based approach | 10.3949/ccjm.87a.ccc037 | Review |  |
| Cheng Q, et al. | Infectivity of human coronavirus in the brain | 10.1016/j.ebiom.2020.102799 | Review |  |
| Corona G, et al. | SARS-CoV-2 infection, male fertility and sperm cryopreservation: a position statement of the Italian Society of Andrology and Sexual Medicine (SIAMS) | 10.1007/s40618-020-01290-w | Review |  |
| Conroy ML, et al. | The COVID-19 AAGP Online Trainee Curriculum: Development and Method of Initial Evaluation | 10.1016/j.jagp.2020.06.003 | Brief Report |  |
| Damle B, et al. | Clinical Pharmacology Perspectives on the Antiviral Activity of Azithromycin and Use in COVID-19 | 10.1002/cpt.1857 | Review |  |
| D'Anna et al. | Characteristics and clinical course of Covid-19 patients admitted with acute stroke | 10.1007/s00415-020-10012-4 | Letter to the editor |  |
| Davanzo R, et al. | Breastfeeding and Coronavirus Disease-2019. Ad interim indications of the Italian Society of Neonatology endorsed by the Union of European Neonatal & Perinatal Societies | 10.1111/mcn.13010 | Review |  |
| de Souza TH, et al. | Clinical manifestations of children with COVID-19: A systematic review | 10.1002/ppul.24885 | Review |  |
| Deeks et al. | Antibody tests for identification of current and past infection with SARS-CoV-2 | 10.1002/14651858.CD013652 | Review |  |
| Dehghanbanadaki et al. | Bibliometric Analysis of Global Scientific Research on SARS CoV-2 (COVID-19) | 10.34171/mjiri.34.51 | Review |  |
| Deng SQ & Peng HJ | Characteristics of and Public Health Responses to the Coronavirus Disease 2019 Outbreak in China. | 10.3390/jcm9020575 | Review |  |
| Dickinson & Gronseth | Application of Universal Design for Learning (UDL) Principles to Surgical Education During the COVID-19 Pandemic | 10.1016/j.jsurg.2020.06.005 | Review |  |
| Docampo-Simón A, et al. | Are chilblain-like acral skin lesions really indicative of COVID-19? A prospective study and literature review | 10.1111/jdv.16665 | Review |  |
| D'Silva KM, et al. | Response to: 'COVID-19 in patients with rheumatological diseases treated with Anti-TNF' by Brito et al and 'Clinical characteristics and outcomes of patients with COVID-19 and rheumatic disease in China 'hot spot' versus in US 'hot spot': similarities and differences' by Zhao et al. | 10.1136/annrheumdis-2020-218196 | Correspondence |  |
| Du YX & Chen XP | Favipiravir: pharmacokinetics and concerns about clinical trials for 2019-nCoV infection | 10.1002/cpt.1844 | Review |  |
| Duan YN, et al. | CT features of novel coronavirus pneumonia (COVID-19) in children | 10.1007/s00330-020-06860-3 | Review |  |
| Ebrahim SH, et al. | All Hands on Deck: A synchronized whole-of-world approach for COVID-19 mitigation | 10.1016/j.ijid.2020.06.049 | Perspective |  |
| Elberry MH, et al. | Occult SARS-CoV-2 infection; a possible hypothesis for viral relapse | 10.1016/j.mehy.2020.109980 | Letter to the editor |  |
| Emami A, et al. | Prevalence of Underlying Diseases in Hospitalized Patients With COVID-19: A Systematic Review and Meta-Analysis | PMID: 32232218 | Review |  |
| Estébanez A, et al. | Cutaneous manifestations in COVID-19: a new contribution | 10.1111/jdv.16474 | Review |  |
| Farsalinos K, et al. | Systematic review of the prevalence of current smoking among hospitalized COVID-19 patients in China: could nicotine be a therapeutic option? | 10.1007/s11739-020-02355-7 | Systematic review |  |
| Feng G, et al. | COVID-19 and Liver Dysfunction: Current Insights and Emergent Therapeutic Strategies | 10.14218/JCTH.2020.00018 | Review |  |
| Fidahic M, et al. | Research methodology and characteristics of journal articles with original data, preprint articles and registered clinical trial protocols about COVID-19 | 10.1186/s12874-020-01047-2 | Review |  |
| Fiorillo L, et al. | COVID-19 Surface Persistence: A Recent Data Summary and Its Importance for Medical and Dental Settings. | 10.3390/ijerph17093132 | Review |  |
| Fu L, et al. | Clinical characteristics of coronavirus disease 2019 (COVID-19) in China: A systematic review and meta-analysis | 10.1016/j.jinf.2020.03.041 | Review |  |
| Galbadage T, et al. | Systematic Review and Meta-Analysis of Sex-Specific COVID-19 Clinical Outcomes | 10.3389/fmed.2020.00348 | Systematic Review |  |
| García IG, et al. | A randomized multicenter clinical trial to evaluate the efficacy of melatonin in the prophylaxis of SARS-CoV-2 infection in high-risk contacts (MeCOVID Trial): A structured summary of a study protocol for a randomised controlled trial | 10.1186/s13063-020-04436-6 | Letter |  |
| Garg S, et al. | Hospitalization Rates and Characteristics of Patients Hospitalized With Laboratory-Confirmed Coronavirus Disease 2019 - COVID-NET, 14 States, March 1-30, 2020 | 10.15585/mmwr.mm6915e3 | CDC Report |  |
| Ge H, et al. | The epidemiology and clinical information about COVID-19 | 10.1007/s10096-020-03874-z | Review |  |
| Gebhard C, et al. | Impact of sex and gender on COVID-19 outcomes in Europe | 10.1186/s13293-020-00304-9 | Review |  |
| Giuliani J. & Bonetti A | Cancer prevales on COVID-19: To maintain high quality standard concerning diagnosis and oncological care even during a pandemic | 10.1002/jmv.26190 | Letter to the editor |  |
| Gopinathannair R, et al. | COVID-19 and cardiac arrhythmias: a global perspective on arrhythmia characteristics and management strategies | 10.1007/s10840-020-00789-9 | Multimedia Report |  |
| Goyal P, et al. | Clinical Characteristics of Covid-19 in New York City | 10.1056/NEJMc2010419 | Letter to the editor |  |
| Griffiths G, et al. | AGILE-ACCORD: A Randomized, Multicentre, Seamless, Adaptive Phase I/II Platform Study to Determine the Optimal Dose, Safety and Efficacy of Multiple Candidate Agents for the Treatment of COVID-19: A structured summary of a study protocol for a randomised platform trial | 10.1186/s13063-020-04473-1 | Letter |  |
| Guan WJ & Zhong NS | Clinical Characteristics of Covid-19 in China. Reply | 10.1056/NEJMc2005203 | Comment |  |
| Guo G, et al. | New Insights of Emerging SARS-CoV-2: Epidemiology, Etiology, Clinical Features, Clinical Treatment, and Prevention | 10.3389/fcell.2020.00410 | Review |  |
| Guo YR, et al. | The origin, transmission and clinical therapies on coronavirus disease 2019 (COVID-19) outbreak - an update on the status | 10.1186/s40779-020-00240-0 | Review |  |
| Han Q, et al. | Coronavirus 2019-nCoV: A brief perspective from the front line. | 10.1016/j.jinf.2020.02.010 | Review |  |
| Haydar A, et al. | Palliative Care Utilization Among Patients With COVID-19 in an Underserved Population: A Single-Center Retrospective Study | 10.1016/j.jpainsymman.2020.05.022 | Comment |  |
| He Y, et al. | Chinese Society of Anesthesiology Expert Consensus on Anesthetic Management of Cardiac Surgical Patients With Suspected or Confirmed Coronavirus Disease 2019 | 10.1053/j.jvca.2020.03.026 | Comment |  |
| Henry BM & Vikse J | Clinical Characteristics of Covid-19 in China | 10.1056/NEJMc2005203 | Comment |  |
| Hong H, et al. | Clinical Characteristics of Novel Coronavirus Disease 2019 (COVID-19) in Newborns, Infants and Children | 10.1016/j.pedneo.2020.03.001 | Comment |  |
| Hu Y, et al. | Prevalence and severity of corona virus disease 2019 (COVID-19): A systematic review and meta-analysis | 10.1016/j.jcv.2020.104371 | Review |  |
| Huang B, et al. | Characteristics of the Coronavirus Disease 2019 and related Therapeutic Options | 10.1016/j.omtm.2020.06.013 | Review |  |
| Huang F, et al. | A review of therapeutic agents and Chinese herbal medicines against SARS-COV-2 (COVID-19) | 10.1016/j.phrs.2020.104929 | Review |  |
| Huang I, et al. | Lymphopenia in severe coronavirus disease-2019 (COVID-19): systematic review and meta-analysis | 10.1186/s40560-020-00453-4 | Review |  |
| Huang J et al. | Characteristics of COVID-19 Clinical Trials in China Based on the Registration Data on ChiCTR and ClinicalTrials.gov | 10.2147/DDDT.S254354 | Review |  |
| Huang X, et al. | Epidemiology and Clinical Characteristics of COVID-19 | 10.34172/aim.2020.09 | Review |  |
| Huang Z, et al. | Occupational exposure to SARS-CoV-2 in burns treatment during the COVID-19 epidemic: Specific diagnosis and treatment protocol. | 10.1016/j.biopha.2020.110176 | Review |  |
| Hussain A, et al. | COVID-19 and Diabetes: Knowledge in Progress | 10.1016/j.diabres.2020.108142 | Review |  |
| Iba T, et al. | The unique characteristics of COVID-19 coagulopathy | 10.1186/s13054-020-03077-0 | Review |  |
| Iordanou S, et al. | Severe SARS-CoV-2 pneumonia in a 58-year-old patient with HIV: a clinical case report from the Republic of Cyprus | 10.1002/jmv.26053 | Letter to the editor |  |
| Ippolito M, et al. | Medical masks and Respirators for the Protection of Healthcare Workers from SARS-CoV-2 and other viruses. | 10.1016/j.pulmoe.2020.04.009 | Review |  |
| Jacobs JJL | Neutralizing antibodies mediate virus-immune pathology of COVID-19 | 10.1016/j.mehy.2020.109884 | Hypothesis |  |
| Jesenak M, et al. | COVID-19, Chronic Inflammatory Respiratory Diseases and Eosinophils - Observationsfrom Reported Clinical Case Series | 10.1111/all.14353 | Letter to the editor |  |
| Jiang C, et al. | Comparative Review of Respiratory Diseases Caused by Coronaviruses and Influenza A Viruses During Epidemic Season | 10.1016/j.micinf.2020.05.005 | Review |  |
| Jiang F, et al. | Review of the Clinical Characteristics of Coronavirus Disease 2019 (COVID-19) | 10.1007/s11606-020-05762-w | Review |  |
| Jin YH, et al. | A rapid advice guideline for the diagnosis and treatment of 2019 novel coronavirus (2019-nCoV) infected pneumonia (standard version) | 10.1186/s40779-020-0233-6 | Practice Guideline |  |
| Jungreis I, et al. | Sarbecovirus comparative genomics elucidates gene content of SARS-CoV-2 and functional impact of COVID-19 pandemic mutations | 10.1101/2020.06.02.130955 | Preprint |  |
| Kang Y & Xu S | Comprehensive overview of COVID-19 based on current evidence. | 10.1111/dth.13525 | Review |  |
| Kasraeian M, et al. | COVID-19 pneumonia and pregnancy; a systematic review and meta-analysis | 10.1080/14767058.2020.1763952 | Review |  |
| Khalil K, et al. | Clinical characteristics and 28-day mortality of medical patients admitted with COVID-19 to a central London teaching hospital | 10.1016/j.jinf.2020.06.027 | Comment |  |
| Khalili M, et al. | Epidemiological characteristics of COVID-19: a systematic review and meta-analysis | 10.1017/S0950268820001430 | Review |  |
| Klein S, et al. | Sex, age, and hospitalization drive antibody responses in a COVID-19 convalescent plasma donor population | 10.1101/2020.06.26.20139063 | Preprint |  |
| Koh J, et al. | Epidemiological and Clinical Characteristics of Cases During the Early Phase of COVID-19 Pandemic: A Systematic Review and Meta-Analysis | 10.3389/fmed.2020.00295 | Review |  |
| Kokou-Kpolou CK, et al. | Prolonged grief related to COVID-19 deaths: Do we have to fear a steep rise in traumatic and disenfranchised griefs? | 10.1037/tra0000798 | Comment |  |
| Kopel J, et al. | Clinical Insights into the Gastrointestinal Manifestations of COVID-19 | 10.1007/s10620-020-06362-8 | Review |  |
| Kowalewski M, et al. | COVID-19 and ECMO: the interplay between coagulation and inflammation-a narrative review. | 10.1186/s13054-020-02925-3 | Review |  |
| Kronbichler A, et al. | Asymptomatic patients as a source of COVID-19 infections: A systematic review and meta-analysis | 10.1016/j.ijid.2020.06.052 | Review |  |
| Lakhani HV, et al. | Systematic Review of Clinical Insights into Novel Coronavirus (CoVID-19) Pandemic: Persisting Challenges in U.S. Rural Population | 10.3390/ijerph17124279 | Review |  |
| Lam HY et al. | The epidemiology of COVID-19 cases and the successful containment strategy in Hong Kong-January to May 2020 | 10.1016/j.ijid.2020.06.057 | Review |  |
| Lee K, et al. | Immunopathogenesis of COVID-19 and early immunomodulators | 10.3345/cep.2020.00759 | Review |  |
| Lei S, et al. | Author's Reply - Clinical Characteristics and Outcomes of Patients Undergoing Surgeries During the Incubation Period of COVID-19 Infection | 10.1016/j.eclinm.2020.100363 | Letter |  |
| Lennon JC | Neurologic and Immunologic Complications of COVID-19: Potential Long-Term Risk Factors for Alzheimer's Disease | 10.3233/ADR-200190 | Short communication |  |
| Leung C | Clinical characteristics of COVID-19 in children: Are they similar to those of SARS? | 10.1002/ppul.24855 | Review |  |
| Li & Fan | Characteristics and Mechanism of Liver Injury in 2019 Coronavirus Disease | 10.14218/JCTH.2020.00019 | Review |  |
| Li C, et al. | Recent progress on the diagnosis of 2019 Novel Coronavirus | 10.1111/tbed.13620 | Review |  |
| Li H, et al. | Coronavirus disease 2019 (COVID-19): current status and future perspectives | 10.1016/j.ijantimicag.2020.105951 | Review |  |
| Li J, et al. | Meta-analysis investigating the relationship between clinical features, outcomes, and severity of severe acute respiratory syndrome coronavirus 2 (SARS-CoV-2) pneumonia | 10.1016/j.ajic.2020.06.008 | Review |  |
| Li J, et al. | Association Between ABO Blood Groups and Risk of SARS-CoV-2 Pneumonia | 10.1111/bjh.16797 | Letter to the editor |  |
| Li JY, et al. | The epidemic of 2019-novel-coronavirus (2019-nCoV) pneumonia and insights for emerging infectious diseases in the future. | 10.1016/j.micinf.2020.02.002 | Review |  |
| Li L, et al. | COVID-19 Patients' Clinical Characteristics, Discharge Rate, and Fatality Rate of Meta-Analysis | 10.1002/jmv.25757 | Review |  |
| Li LY, et al. | Digestive system involvement of novel coronavirus infection: prevention and control infection from a gastroenterology perspective | 10.1111/1751-2980.12862 | Review |  |
| Li MD, et al. | Automated assessment of COVID-19 pulmonary disease severity on chest radiographs using convolutional Siamese neural networks | 10.1101/2020.05.20.20108159 | Preprint |  |
| Li T, et al. | Clinical Observation and Management of COVID-19 Patients | 10.1080/22221751.2020.1741327 | Comment |  |
| Li W, et al. | The characteristics of two patients coinfected with SARS-CoV-2 and HIV in Wuhan, China | 10.1002/jmv.26155 | Letter to the Editor |  |
| Li X & Ma X | Acute respiratory failure in COVID-19: is it "typical" ARDS? | 10.1186/s13054-020-02911-9 | Review |  |
| Li YC, et al. | The neuroinvasive potential of SARS-CoV2 may play a role in the respiratory failure of COVID-19 patients. | 10.1002/jmv.25728 | Review |  |
| Lin HY | The severe COVID-19: A sepsis induced by viral infection? And its immunomodulatory therapy | 10.1016/j.cjtee.2020.06.002 | Review |  |
| Lingeswaran M, et al. | Inflammation, Immunity and Immunogenetics in COVID-19: A Narrative Review | 10.1007/s12291-020-00897-3 | Review |  |
| Lippi G, et al. | COVID-19: unravelling the clinical progression of nature's virtually perfect biological weapon | 10.21037/atm-20-3989 | Review |  |
| Lippi G, et al. | Clinical and Demographic Characteristics of Patients Dying From COVID-19 in Italy Versus China | 10.1002/jmv.25860 | Letter to the editor |  |
| Liu F, et al. | Clinically significant portal hypertension in cirrhosis patients with COVID-19: Clinical characteristics and outcomes | 10.1016/j.jinf.2020.06.029 | Comment |  |
| Liu K, et al. | Clinical features of COVID-19 in elderly patients: A comparison with young and middle-aged patients | 10.1016/j.jinf.2020.03.005 | Review |  |
| Liu N, et al. | COVID-19 Pandemic: Experiences in China and Implications for Its Prevention and Treatment Worldwide | 10.2174/1568009620666200414151419 | Review |  |
| Liya G, et al. | Studies on viral pneumonia related to novel coronavirus SARS- CoV-2, SARS-CoV, and MERS-CoV: a literature review | 10.1111/apm.13047 | Review |  |
| Long-Quan L, et al. | Response to Char's comment: Comment on Li et al.: COVID-19 patients' clinical characteristics, discharge rate, and fatality rate of meta-analysis | 10.1002/jmv.25924 | Comment |  |
| Lotfi M, et al. | SARS-CoV-2: A comprehensive review from pathogenicity of the virus to clinical consequences | 10.1002/jmv.26123 | Review |  |
| Lovato A, et al. | Clinical characteristics associated with persistent olfactory and taste alterations in COVID-19: A preliminary report on 121 patients | 10.1016/j.amjoto.2020.102548 | Comment |  |
| Lovato A, et al. | Sore throat in COVID‐19: Comment on “Clinical characteristics of hospitalized patients with SARS‐CoV‐2 infection: A single arm meta‐analysis” | 10.1002/jmv.25815 | Letter to the editor |  |
| Ma J, et al. | Clinical Characteristics and Prognosis in Cancer Patients With COVID-19: A Single Center's Retrospective Study | 10.1016/j.jinf.2020.04.006 | Letter to the editor |  |
| Ma X, et al. | The clinical characteristics of pediatric inpatients with SARS-CoV-2 infection: A meta-analysis and systematic review | 10.1002/jmv.26208 | Review |  |
| Ma XL, et al. | Management strategies of neonatal jaundice during the coronavirus disease 2019 outbreak | 10.1007/s12519-020-00347-3 | Review |  |
| MacIntyre CR, et al. | Human coronavirus data from four clinical trials of masks and respirators | 10.1016/j.ijid.2020.05.092 | Review |  |
| Maguire BJ & Guérin PJ | A Living Systematic Review Protocol for COVID-19 Clinical Trial Registrations | 10.12688/wellcomeopenres.15821.1 | Review |  |
| Maida FD, et al. | Letter to the Editor: "Clinical characteristics and outcomes of patients undergoing surgeries during the incubation period of COVID-19 infection". | 10.1016/j.eclinm.2020.100362 | Letter to the editor |  |
| Mallineni SK, et al. | Coronavirus Disease (COVID-19): Characteristics in Children and Considerations for Dentists Providing Their Care | 10.1111/ipd.12653 | Ediorial |  |
| Mantovani A, et al. | Coronavirus disease 2019 (COVID-19) in children and/or adolescents: a meta-analysis | 10.1038/s41390-020-1015-2 | Review |  |
| Mason RJ | Thoughts on the alveolar phase of COVID-19 | 10.1152/ajplung.00126.2020 | Review |  |
| Matar R, et al. | Clinical Presentation and Outcomes of Pregnant Women with COVID-19: A Systematic Review and Meta-Analysis | 10.1093/cid/ciaa828 | Review |  |
| Mathew D, et al. | Deep immune profiling of COVID-19 patients reveals patient heterogeneity and distinct immunotypes with implications for therapeutic interventions | 10.1101/2020.05.20.106401 | Preprint |  |
| McCreary EK & Pogue JM | Coronavirus Disease 2019 Treatment: A Review of Early and Emerging Options | 10.1093/ofid/ofaa105 | Review |  |
| Meena et al. | Clinical Features and Outcome of SARS-CoV-2 Infection in Children: A Systematic Review and Meta-analysis | 10.1007/s13312-020-1961-0 | Review |  |
| Meng X, et al. | COVID-19 and anosmia: A review based on up-to-date knowledge | 10.1016/j.amjoto.2020.102581 | Review |  |
| Merkler AE, et al. | Ischemic Stroke in Patients with Covid-19 versus Patients with Influenza | 10.1101/2020.05.18.20105494 | Preprint |  |
| Merkus PJFM, et al. | The value of chest CT as a COVID-19 screening tool in children | 10.1183/13993003.01241-2020. | Letter to the Editor |  |
| Mi B, et al. | Surgery in the COVID‐19 pandemic: clinical characteristics and outcomes | 10.1002/bjs.11733 | Correspondence |  |
| Mo X, et al. | Abnormal pulmonary function in COVID-19 patients at time of hospital discharge | 10.1183/13993003.01217-2020 | Letter to the Editor |  |
| Morabito M, et al. | Heat warning and public and workers' health at the time of COVID-19 pandemic | 10.1016/j.scitotenv.2020.140347 | Comment |  |
| Moujaess E, et al. | Cancer patients and research during COVID-19 pandemic: A systematic review of current evidence | 10.1016/j.critrevonc.2020.102972 | Review |  |
| Nelson PP, et al. | Current and Future Point-of-Care Tests for Emerging and New Respiratory Viruses and Future Perspectives. | 10.3389/fcimb.2020.00181 | Mini Review |  |
| No authors listed | Corrigendum to Glycemic Characteristics and Clinical Outcomes of COVID-19 Patients Hospitalized in the United States | 10.1177/1932296820932678 | Retracted |  |
| Olaguibel JM, et al. | Upper and Lower Airways Functional Examination in Asthma and Respiratory Allergic Deseases. Considerations in the SARS-CoV-2 Post-Pandemic Situation | 10.18176/jiaci.0625 | Review |  |
| Ozaras R, et al. | Influenza and COVID-19 coinfection: Report of six cases and review of the literature | 10.1002/jmv.26125 | Review |  |
| Pan X, et al. | Potential drugs for the treatment of the novel coronavirus pneumonia (COVID-19) in China | 10.1016/j.virusres.2020.198057 | Review |  |
| Parasa S, et al. | Prevalence of Gastrointestinal Symptoms and Fecal Viral Shedding in Patients With Coronavirus Disease 2019: A Systematic Review and Meta-analysis | 10.1001/jamanetworkopen.2020.11335 | Review |  |
| Park M, et al. | A Systematic Review of COVID-19 Epidemiology Based on Current Evidence | 10.3390/jcm9040967 | Review |  |
| Pedersen SF & Ho YC | SARS-CoV-2: a storm is raging | 10.1172/JCI137647 | Comment |  |
| Pence BD | Severe COVID-19 and aging: are monocytes the key? | 10.1007/s11357-020-00213-0 | Review |  |
| Phan T | Novel Coronavirus: From Discovery to Clinical Diagnostics | 10.1016/j.meegid.2020.104211 | Opinion |  |
| Pongpirul WA, et al. | Clinical Characteristics of Patients Hospitalized With Coronavirus Disease, Thailand | 10.3201/eid2607.200598 | Letter to the editor |  |
| Pormohammad A, et al. | Comparison of confirmed COVID-19 with SARS and MERS cases - Clinical characteristics, laboratory findings, radiographic signs and outcomes: A systematic review and meta-analysis | 10.1002/rmv.2112 | Review |  |
| Posada-Vergara et al. | COVID-19 and HIV | 10.25100/cm.v51i2.4327 | Review |  |
| Prayuenyong P, et al. | Clinical Implications of Chloroquine and Hydroxychloroquine Ototoxicity for COVID-19 Treatment: A Mini-Review | 10.3389/fpubh.2020.00252 | Review |  |
| Qian JY, et al. | Acute Kidney Injury in the 2019 Novel Coronavirus Disease | 10.1159/000509086 | Review |  |
| Qu JM, et al. | Guidance for the Management of Adult Patients With Coronavirus Disease 2019 | 10.1097/CM9.0000000000000899 | Clinical Guideline |  |
| Raba AA, et al. | Novel coronavirus infection (COVID-19) in children younger than one year: A systematic review of symptoms, management and outcomes | 10.1111/apa.15422 | Review |  |
| Razonable RR, et al. | A Collaborative Multidisciplinary Approach to the Management of Coronavirus Disease 2019 in the Hospital Setting | 10.1016/j.mayocp.2020.05.010 | Review |  |
| Ren YR, et al. | Comprehensive Updated Review on SARS-CoV-2 and COVID-19 | 10.1002/jcph.1673 | Review |  |
| Rhodes NJ, et al. | Multicenter point-prevalence evaluation of the utilization and safety of drug therapies for COVID-19 | 10.1101/2020.06.03.20121558 | Preprint |  |
| Ribeiro VST, et al. | Arboviral diseases and COVID-19 in Brazil: Concerns regarding climatic, sanitation, and endemic scenario | 10.1002/jmv.26079 | Letter to the Editor |  |
| Richards W | Being a dentist in the pandemic | 10.1038/s41432-020-0095-5 | Comment |  |
| Robbins-Juarez SY, et al. | Outcomes for Patients With COVID-19 and Acute Kidney Injury: A Systematic Review and Meta-Analysis | 10.1016/j.ekir.2020.06.013 | Review |  |
| Rolf JD | Clinical Characteristics of Covid-19 in China | 10.1056/NEJMc2005203 | Comment |  |
| Saghazadeh & Rezaei | Towards treatment planning of COVID-19: Rationale and hypothesis for the use of multiple immunosuppressive agents: Anti-antibodies, immunoglobulins, and corticosteroids. | 10.1016/j.intimp.2020.106560 | Review |  |
| Salehi S, et al. | Coronavirus Disease 2019 (COVID-19): A Systematic Review of Imaging Findings in 919 Patients. | 10.2214/AJR.20.23034 | Review |  |
| Santaella-Tenorio J | SARS-CoV-2 diagnostic testing alternatives for Latin America | 10.25100/cm.v51i2.4272 | Review |  |
| Sarapultsev A, et al. | Immunological environment shifts during pregnancy may affect the risk of developing severe complications in COVID-19 patients | 10.1111/aji.13285 | Letter to the Editor |  |
| Schultheiß C, et al. | Next-Generation Sequencing of T and B Cell Receptor Repertoires from COVID-19 Patients Showed Signatures Associated with Severity of Disease | 10.1016/j.immuni.2020.06.024 | Comment |  |
| Semeraro F, et al. | New Early Warning Score: off-label approach for Covid-19 outbreak patient deterioration in the community | 10.1016/j.resuscitation.2020.04.018 | Letter to the Editor |  |
| Shashikumar SP, et al. | Development and Prospective Validation of a Transparent Deep Learning Algorithm for Predicting Need for Mechanical Ventilation | 10.1101/2020.05.30.20118109 | Preprint |  |
| She J, et al. | COVID-19 Epidemic: Disease Characteristics in Children | 10.1002/jmv.25807 | Review |  |
| Shereen MA, et al. | COVID-19 Infection: Origin, Transmission, and Characteristics of Human Coronaviruses | 10.1016/j.jare.2020.03.005 | Review |  |
| Shi W, et al. | Clinical characteristics of COVID‐19 patients combined with allergy | 10.1111/all.14434 | Letter to the Editor |  |
| Simpson CR, et al. | Pandemic Evaluation and Enhanced Surveillance of COVID-19 (EAVE II): protocol for an observational study using linked Scottish national data | 10.1136/bmjopen-2020-039097 | Protocol |  |
| Somers EC, et al. | Tocilizumab for treatment of mechanically ventilated patients with COVID-19 | 10.1101/2020.05.29.20117358 | Preprint |  |
| Song P & Karako T | COVID-19: Real-time dissemination of scientific information to fight a public health emergency of international concern | 10.5582/bst.2020.01056 | Editorial |  |
| Stojkovic-Filipovic J & Bosic M | Treatment of COVID 19-Repurposing drugs commonly used in dermatology | 10.1111/dth.13829 | Review |  |
| Stower H | Clinical and Epidemiological Characteristics of Children With COVID-19 | 10.1007/s11427-020-1643-8 | Comment |  |
| Su & Lai | Comparison of clinical characteristics of coronavirus disease (COVID-19) and severe acute respiratory syndrome (SARS) as experienced in Taiwan | 10.1016/j.tmaid.2020.101625 | Letter to the editor |  |
| Suleyman G, et al. | Clinical Characteristics and Morbidity Associated With Coronavirus Disease 2019 in a Series of Patients in Metropolitan Detroit | 10.1001/jamanetworkopen.2020.12270 | Review |  |
| Sultan LR, et al. | A Review of Early Experience in Lung Ultrasound in the Diagnosis and Management of COVID-19 | 10.1016/j.ultrasmedbio.2020.05.012 | Review |  |
| Sun C, et al. | Role of the Eye in Transmitting Human Coronavirus: What We Know and What We Do Not Know. | 10.3389/fpubh.2020.00155 | Review |  |
| Sun P, et al. | Clinical characteristics of hospitalized patients with SARS-CoV-2 infection: A single arm meta-analysis | 10.1002/jmv.25735 | Review |  |
| Sun P, et al. | Response to: Sore throat in COVID-19: comment on "Clinical characteristics of hospitalized patients with SARS-CoV-2 infection: A single arm meta-analysis" | 10.1002/jmv.25818 | Letter to the editor |  |
| Sun T & Guan J | Novel coronavirus and central nervous system. | 10.1111/ene.14227 | Letter to the editor |  |
| Taghizadeh-Hesary F & Akbari H | The Powerful Immune System Against Powerful COVID-19: A Hypothesis | 10.1016/j.mehy.2020.109762 | Hypothesis |  |
| Tang D, et al. | The hallmarks of COVID-19 disease | 10.1371/journal.ppat.1008536 | Review |  |
| Tang LY &, Wang J | Anesthesia and COVID-19: What We Should Know and What We Should Do | 10.1177/1089253220921590 | Review |  |
| Temesgen Z, et al. | First Clinical Use of Lenzilumab to Neutralize GM-CSF in Patients with Severe COVID-19 Pneumonia | 10.1101/2020.06.08.20125369 | Preprint |  |
| Tolia VM, et al. | Preliminary Results of Initial Testing for Coronavirus (COVID-19) in the Emergency Department | 10.5811/westjem.2020.3.47348 | Review |  |
| Toombs JM, et al. | COVID-19 in three people living with HIV in the United Kingdom | 10.1002/jmv.26178 | Letter to the editor |  |
| Toraih EA, et al. | Association of cardiac biomarkers and comorbidities with increased mortality, severity, and cardiac injury in COVID-19 patients: A meta-regression and decision tree analysis | 10.1002/jmv.26166 | Review |  |
| Tsivgoulis G, et al. | Neurological manifestations and implications of COVID-19 pandemic | 10.1177/1756286420932036 | Review |  |
| Tu Y, et al. | A Review of SARS-CoV-2 and the Ongoing Clinical Trials. | 10.3390/ijms21072657 | Review |  |
| Udugama B, et al. | Diagnosing COVID-19: The Disease and Tools for Detection | 10.1021/acsnano.0c02624 | Review |  |
| Vardavas CI & Nikitara K | COVID-19 and smoking | 10.18332/tid/119324 | Systematic review |  |
| Viswanathan R, et al. | Support Groups and Individual Mental Health Care via Video Conferencing for Frontline Clinicians During the COVID-19 Pandemic | 10.1016/j.psym.2020.06.014 | Perspective |  |
| Wang A, et al. | Timely blood glucose management for the outbreak of 2019 novel coronavirus disease (COVID-19) is urgently needed. | 10.1016/j.diabres.2020.108118 | Comment |  |
| Wang B, et al. | A tertiary center experience of multiple myeloma patients with COVID-19: lessons learned and the path forward | 10.1101/2020.06.04.20122846 | Preprint |  |
| Wang L, et al. | Review of the 2019 novel coronavirus (SARS-CoV-2) based on current evidence | 10.1016/j.ijantimicag.2020.105948 | Review |  |
| Wang X, et al. | Comorbid Chronic Diseases and Acute Organ Injuries Are Strongly Correlated with Disease Severity and Mortality among COVID-19 Patients: A Systemic Review and Meta-Analysis. | 10.34133/2020/2402961 | Systematic Review |  |
| Wei M, et al. | Novel Coronavirus Infection in Hospitalized Infants Under 1 Year of Age in China | 10.1001/jama.2020.2131 | Letter to the editor |  |
| Wen J, et al. | Potential therapeutic effect of Qingwen Baidu Decoction against Corona Virus Disease 2019: a mini review | 10.1186/s13020-020-00332-y | Review |  |
| Weng CH, et al. | Characteristics and Clinical Course and Outcome of COVID‐19 in Hispanic/Latino Patients in a Community Setting: A Retrospective Cohort Study | 10.1002/jmv.26196 | Letter to the editor |  |
| Weng CH, et al. | Differences in clinical characteristics of COVID-19 in Hispanic/Latino population | 10.1111/tbed.13664 | Letter to the Editor |  |
| Wilder-Smith A, et al. | Can We Contain the COVID-19 Outbreak With the Same Measures as for SARS? | 10.1016/S1473-3099(20)30129-8 | Review |  |
| Wong MC, et al. | Detection of SARS-CoV-2 RNA in fecal specimens of patients with confirmed COVID-19: A meta-analysis | 10.1016/j.jinf.2020.06.012 | Review |  |
| Wu F, et al. | SARS-CoV-2 titers in wastewater foreshadow dynamics and clinical presentation of new COVID-19 cases | 10.1101/2020.06.15.20117747 | Preprint |  |
| Wu Q, et al. | Recovery from COVID-19 in two patients with coexisted HIV infection. | 10.1002/jmv.26006 | Letter to the editor |  |
| Wu Q, et al. | Co-infection and Other Clinical Characteristics of COVID-19 in Children. | 10.1542/peds.2020-0961 | Review |  |
| Xing Y, et al. | Prolonged viral shedding in feces of pediatric patients with coronavirus disease 2019 | 10.1016/j.jmii.2020.03.021 | Review |  |
| Xiong XL, et al. | Comparative study of the clinical characteristics and epidemiological trend of 244 COVID-19 infected children with or without GI symptoms | 10.1136/gutjnl-2020-321486 | Letter to the Editor |  |
| Xu Y, et al. | Significance of Serology Testing to Assist Timely Diagnosis of SARS-CoV-2 Infections: Implication From a Family Cluster | 10.1080/22221751.2020.1752610 | Letter to the editor |  |
| Yadaw AS, et al. | Clinical predictors of COVID-19 mortality | 10.1101/2020.05.19.20103036 | Preprint |  |
| Yang C, et al. | Coronavirus disease 2019: reassembly attack of coronavirus | 10.1080/09603123.2020.1747602 | Review |  |
| Yang H & Lu S | COVID-19 and Tuberculosis | 10.2478/jtim-2020-0010 | Review |  |
| Yang J, et al. | Characteristics and Challenges of Psychological First Aid in China During the COVID-19 Outbreak | 10.1016/j.bbi.2020.04.075 | Letter to the editor |  |
| Yang J, et al. | Prevalence of comorbidities in the novel Wuhan coronavirus (COVID-19) infection: a systematic review and meta-analysis | 10.1016/j.ijid.2020.03.017 | Meta-analysis |  |
| Yang M, et al. | Characteristics of registered studies for Coronavirus disease 2019 (COVID-19): A systematic review | 10.1016/j.imr.2020.100426 | Review |  |
| Yang W, et al. | The role of imaging in 2019 novel coronavirus pneumonia (COVID-19) | 10.1007/s00330-020-06827-4 | Review |  |
| Yang Z, et al. | Coronavirus disease 2019 (COVID-19) and pregnancy: a systematic review | 10.1080/14767058.2020.1759541 | Systematic Review |  |
| Yao Y, et al. | Epidemiological Characteristics of 2019-ncoV Infections in Shaanxi, China by February 8, 2020 | 10.1183/13993003.00310-2020 | Letter to the editor |  |
| Younes N, et al. | Challenges in Laboratory Diagnosis of the Novel Coronavirus SARS-CoV-2 | 10.3390/v12060582 | Review |  |
| Yu et al. | Recent Understandings Toward Coronavirus Disease 2019 (COVID-19): From Bench to Bedside | 10.3389/fcell.2020.00476 | Review |  |
| Yu J, et al. | SARS-CoV-2 Transmission in Patients With Cancer at a Tertiary Care Hospital in Wuhan, China | 10.1001/jamaoncol.2020.0980 | Letter to the editor |  |
| Zavascki & Falci | Clinical Characteristics of Covid-19 in China | 10.1056/NEJMc2005203 | Comment |  |
| Zhang D, et al. | Viral aetiology and clinical characteristics of acute respiratory tract infections in Shenzhen during epidemic of coronavirus disease 2019 | 10.1080/23744235.2020.1769855 | Comment |  |
| Zhang et al. | Solid Organ Transplantation During the COVID-19 Pandemic | 10.3389/fimmu.2020.01392 | Review |  |
| Zhang LP, et al. | Focus on the 2019 novel coronavirus (SARS-CoV-2) | 10.2217/fmb-2020-0063 | Review |  |
| Zhang W, et al. | The use of anti-inflammatory drugs in the treatment of people with severe coronavirus disease 2019 (COVID-19): The Perspectives of clinical immunologists from China | 10.1016/j.clim.2020.108393 | Review |  |
| Zhao C, et al. | Otolaryngology during COVID-19: Preventive care and precautionary measures | 10.1016/j.amjoto.2020.102508 | Review |  |
| Zhao J, et al. | Clinical characteristics and outcomes of patients with COVID-19 and rheumatic disease in China 'hot spot' versus in US 'hot spot': similarities and differences | 10.1136/annrheumdis-2020-218183 | Correspondence |  |
| Zhao Q, et al. | COVID-19 and cutaneous manifestations: a systematic review | 10.1111/jdv.16778 | Review |  |
| Zhao YF, et al. | Prediction of the Number of Patients Infected with COVID-19 Based on Rolling Grey Verhulst Models | 10.3390/ijerph17124582 | Review |  |
| Zhao Z, et al. | Recommendations of individualized medical treatment and common adverse events management for lung cancer patients during the outbreak of COVID-19 epidemic | 10.1111/1759-7714.13424 | Clinical Guideline |  |
| Zhen-Dong Y, et al. | Clinical and transmission dynamics characteristics of 406 children with coronavirus disease 2019 in China: A review | 10.1016/j.jvs.2020.04.483 | Review |  |
| Zheng J, et al. | SARS-CoV-2: an Emerging Coronavirus that Causes a Global Threat. | 10.7150/ijbs.45053 | Review |  |
| Zheng Y, et al. | Immunoregulation with mTOR inhibitors to prevent COVID-19 severity: A novel intervention strategy beyond vaccines and specific antiviral medicines | 10.1002/jmv.26009 | Review |  |
| Zhou B, et al. | The Clinical Characteristics of Myocardial Injury in Severe and Very Severe Patients With 2019 Novel Coronavirus Disease | 10.1016/j.jinf.2020.03.021 | Letter to the editor |  |
| Zhou H, et al. | Potential therapeutic targets and promising drugs for combating SARS-CoV-2 | 10.1111/bph.15092 | Review |  |
| Zhou M, et al. | Serological characteristics of COVID-19 patients | 10.1080/23744235.2020.1784997 | Letter to the editor |  |
| Zhou M, et al. | Coronavirus Disease 2019 (COVID-19): A Clinical Update | 10.1007/s11684-020-0767-8 | Review |  |
| Zhou M, et al. | From SARS to COVID-19: What we have learned about children infected with COVID-19. | 10.1016/j.ijid.2020.04.090 | Review |  |
| Zhou Y, et al. | Obesity and diabetes as high-risk factors for severe coronavirus disease 2019 (Covid-19) | 10.1002/dmrr.3377 | Review |  |
| Zhou Y, et al. | Comment on Li et al.: COVID-19 patients' clinical characteristics, discharge rate, and fatality rate of meta-analysis. | 10.1002/jmv.25912 | Comment |  |
| Zhu J, et al. | Clinical Characteristics of 3,062 COVID-19 Patients: A Meta-Analysis | 10.1002/jmv.25884 | Meta-analysis |  |
| Bauer DC, et al. | Supporting pandemic response using genomics and bioinformatics: A case study on the emergent SARS-CoV-2 outbreak | 10.1111/tbed.13588 | Rapid communication | Did not mentioned "clinical characteristics" anywhere in the full text |
| Bullard J, et al. | Predicting infectious SARS-CoV-2 from diagnostic samples | 10.1093/cid/ciaa638 | Original research | Did not mentioned "clinical characteristics" anywhere in the full text |
| Cagnacci A, et al. | Age-related difference in the rate of coronavirus disease 2019 mortality in women versus men | 10.1016/j.ajog.2020.05.039 | Original research | Did not mentioned "clinical characteristics" anywhere in the full text |
| Caly L, et al. | Isolation and rapid sharing of the 2019 novel coronavirus (SARS-CoV-2) from the first patient diagnosed with COVID-19 in Australia | 10.5694/mja2.50569 | Original research | Did not mentioned "clinical characteristics" anywhere in the full text |
| Camiolo M, et al. | Expression of SARS-CoV-2 receptor ACE2 and coincident host response signature varies by asthma inflammatory phenotype | 10.1016/j.jaci.2020.05.051 | Original research | Did not mentioned "clinical characteristics" anywhere in the full text |
| Cheng VCC, et al. | Escalating Infection Control Response to the Rapidly Evolving Epidemiology of the Coronavirus Disease 2019 (COVID-19) Due to SARS-CoV-2 in Hong Kong | 10.1017/ice.2020.58 | Original research | Did not mentioned "clinical characteristics" anywhere in the full text |
| d'Alessandro M, et al. | Serum KL-6 concentrations as a novel biomarker of severe COVID-19 | 10.1002/jmv.26087 | Original research | Did not mentioned "clinical characteristics" anywhere in the full text |
| Davies NG, et al. | Effects of non-pharmaceutical interventions on COVID-19 cases, deaths, and demand for hospital services in the UK: a modelling study | 10.1016/S2468-2667(20)30133-X | Original research | Did not mentioned "clinical characteristics" anywhere in the full text |
| El Hachem M, et al. | A clinical, histopathological and laboratory study of 19 consecutive Italian paediatric patients with chilblain-like lesions: lights and shadows on the relationship with COVID-19 infection | 10.1111/jdv.16682 | Original research | Did not mentioned "clinical characteristics" anywhere in the full text |
| Fan N, et al. | Imaging characteristics of initial chest computed tomography and clinical manifestations of patients with COVID-19 pneumonia | 10.1007/s11604-020-00973-x | Original research | Did not mentioned "clinical characteristics" anywhere in the full text |
| Ferrazzi E, et al. | Vaginal delivery in SARS-CoV-2-infected pregnant women in Northern Italy: a retrospective analysis | 10.1111/1471-0528.16278 | Original research | Did not mentioned "clinical characteristics" anywhere in the full text |
| Holt A, et al. | New-onset atrial fibrillation: incidence, characteristics, and related events following a national COVID-19 lockdown of 5.6 million people | 10.1093/eurheartj/ehaa494 | Original research | Did not mentioned "clinical characteristics" anywhere in the full text |
| Hu S, et al. | Computed tomography manifestations in super early stage 2019 novel coronavirus pneumonia | 10.1177/0284185120924806 | Original research | Did not mentioned "clinical characteristics" anywhere in the full text |
| Lauer SA, et al. | The Incubation Period of Coronavirus Disease 2019 (COVID-19) From Publicly Reported Confirmed Cases: Estimation and Application | 10.7326/M20-0504 | Original research | Did not mentioned "clinical characteristics" anywhere in the full text |
| Lieberman JA, et al. | Comparison of Commercially Available and Laboratory Developed Assays for in vitro Detection of SARS-CoV-2 in Clinical Laboratories | 10.1128/JCM.00821-20 | Original research | Did not mentioned "clinical characteristics" anywhere in the full text |
| Liu X, et al. | Prediction of the severity of Corona Virus Disease 2019 and its adverse clinical outcomes | 10.7883/yoken.JJID.2020.194 | Original research | Did not mentioned "clinical characteristics" anywhere in the full text |
| Liu X, et al. | Single-cell Transcriptome Analysis of the Novel Coronavirus (SARS-CoV-2) Associated Gene ACE2 Expression in Normal and Non-Obstructive Azoospermia (NOA) Human Male Testes | 10.1007/s11427-020-1705-0 | Original research | Did not mentioned "clinical characteristics" anywhere in the full text |
| Long CJ, et al. | Imaging features of the initial chest thin-section CT scans from 110 patients after admission with suspected or confirmed diagnosis of COVID-19 | 10.1186/s12880-020-00464-5 | Original research | Did not mentioned "clinical characteristics" anywhere in the full text |
| Long DR, et al. | Occurrence and Timing of Subsequent SARS-CoV-2 RT-PCR Positivity Among Initially Negative Patients | 10.1093/cid/ciaa722 | Original research | Did not mentioned "clinical characteristics" anywhere in the full text |
| Mei F, et al. | First Detection of SARS-CoV-2 by Real-Time Reverse Transcriptase-Polymerase Chain Reaction Assay in Pleural Fluid | 10.1016/j.chest.2020.05.583 | Case Reports | Did not mentioned "clinical characteristics" anywhere in the full text |
| Moccia L, et al. | Affective Temperament, Attachment Style, and the Psychological Impact of the COVID-19 Outbreak: An Early Report on the Italian General Population | 10.1016/j.bbi.2020.04.048 | Original research | Did not mentioned "clinical characteristics" anywhere in the full text |
| Paraskevis D, et al. | Full-genome evolutionary analysis of the novel corona virus (2019-nCoV) rejects the hypothesis of emergence as a result of a recent recombinationevent | 10.1016/j.meegid.2020.104212 | Short communication | Did not mentioned "clinical characteristics" anywhere in the full text |
| Prokop M, et al. | CO-RADS - A Categorical CT Assessment Scheme for Patients With Suspected COVID-19: Definition and Evaluation | 10.1148/radiol.2020201473 | Original research | Did not mentioned "clinical characteristics" anywhere in the full text |
| Roca-Ginés, J et al. | Assessment of Acute Acral Lesions in a Case Series of Children and Adolescents During the COVID-19 Pandemic | 10.1001/jamadermatol.2020.2340 | Original research | Did not mentioned "clinical characteristics" anywhere in the full text |
| Qiu C, et al. | Olfactory and Gustatory Dysfunction as an Early Identifier of COVID-19 in Adults and Children: An International Multicenter Study | 10.1177/0194599820934376 | Original research | Did not mentioned "clinical characteristics" anywhere in the full text |
| Tran BX, et al. | Coverage of Health Information by Different Sources in Communities: Implication for COVID-19 Epidemic Response | 10.3390/ijerph17103577 | Original research | Did not mentioned "clinical characteristics" anywhere in the full text |
| van Dam LF, et al. | Clinical and computed tomography characteristics of COVID-19 associated acute pulmonary embolism: A different phenotype of thrombotic disease? | 10.1016/j.thromres.2020.06.010 | Original research | Did not mentioned "clinical characteristics" anywhere in the full text |
| Vanni G, et al. | Breast Cancer and COVID-19: The Effect of Fear on Patients' Decision-making Process | 10.21873/invivo.11957 | Original research | Did not mentioned "clinical characteristics" anywhere in the full text |
| Wang H, et al. | The psychological distress and coping styles in the early stages of the 2019 coronavirus disease (COVID-19) epidemic in the general mainland Chinese population: A web-based survey. | 10.1371/journal.pone.0233410 | Original research | Did not mentioned "clinical characteristics" anywhere in the full text |
| Zhang FY, et al. | CT Imaging of the COVID-19 | 10.1016/j.jfma.2020.04.006 | Original research | Did not mentioned "clinical characteristics" anywhere in the full text |
| Zhang L, et al. | Early characteristics of the COVID-19 outbreak predict the subsequent epidemic scope | 10.1016/j.ijid.2020.05.122 | Original research | Did not mentioned "clinical characteristics" anywhere in the full text |
| Zifheng J, et al. | Consistency Analysis of COVID-19 Nucleic Acid Tests and the Changes of Lung CT | 10.1016/j.jcv.2020.104359 | Original research | Did not mentioned "clinical characteristics" anywhere in the full text |
| Abouelkhair MA. | Targeting adenosinergic pathway and adenosine A 2A receptor signaling for the treatment of COVID-19: A hypothesis | 10.1016/j.mehy.2020.110012 | Original research | The study did not analyzed COVID-19 patients |
| Ahamad MM | A machine learning model to identify early stage symptoms of SARS-Cov-2 infected patients | 10.1016/j.eswa.2020.113661 | Original research | The study did not analyzed COVID-19 patients |
| Barratt R, et al. | Characteristics of personal protective equipment training programs in Australia and New Zealand hospitals: A survey | 10.1016/j.idh.2020.05.005 | Original research | The study did not analyzed COVID-19 patients |
| Bianchi M, et al. | Sars-CoV-2 Envelope and Membrane Proteins: Structural Differences Linked to Virus Characteristics? | 10.1155/2020/4389089 | Original research | The study did not analyzed COVID-19 patients |
| Bromage DI, et al. | The impact of COVID-19 on heart failure hospitalization and management: report from a Heart Failure Unit in London during the peak of the pandemic | 10.1002/ejhf.1925 | Original research | The study did not analyzed COVID-19 patients |
| Carter E, et al. | The first six weeks - setting up a UK urgent dental care centre during the COVID-19 pandemic | 10.1038/s41415-020-1708-2 | Original Article | The study did not analyzed COVID-19 patients |
| Chu H, et al. | Comparative Replication and Immune Activation Profiles of SARS-CoV-2 and SARS-CoV in Human Lungs: An Ex Vivo Study With Implications for the Pathogenesis of COVID-19 | 10.1093/cid/ciaa410 | Original research | The study did not analyzed COVID-19 patients |
| Ciaccio M & Agnello L | Biochemical biomarkers alterations in Coronavirus Disease 2019 (COVID-19) | 10.1515/dx-2020-0057 | Original research | The study did not analyzed COVID-19 patients |
| Compton S, et al. | Medical students' preference for returning to the clinical setting during the COVID-19 pandemic | 10.1111/medu.14268 | Original Article | The study did not analyzed COVID-19 patients |
| Gan X, et al. | Willingness of Chinese nurses to practice in Hubei combating the coronavirus disease 2019 epidemic: A cross-sectional study | 10.1111/jan.14434 | Original research | The study did not analyzed COVID-19 patients |
| Gao X, et al. | Characterization, pathogenicity and protective efficacy of a cell culture-derived porcine deltacoronavirus | 10.1016/j.virusres.2020.197955 | Original research | The study did not analyzed COVID-19 patients |
| Gonzalez-Reiche AS, et al. | Introductions and early spread of SARS-CoV-2 in the New York City area | 10.1126/science.abc1917 | Original research | The study did not analyzed COVID-19 patients |
| Hamza MS, et al. | Cross-Sectional Study on Awareness and Knowledge of COVID-19 Among Senior pharmacy Students | 10.1007/s10900-020-00859-z | Original Article | The study did not analyzed COVID-19 patients |
| Hernández-García I & Giménez-Júlvez T | Characteristics of YouTube Videos in Spanish on How to Prevent COVID-19 | 10.3390/ijerph17134671 | Original research | The study did not analyzed COVID-19 patients |
| Homolak J, et al. | Widely available lysosome targeting agents should be considered as potential therapy for COVID-19 | 10.1016/j.ijantimicag.2020.106044 | Original Article | The study did not analyzed COVID-19 patients |
| Hou C, et al. | The Effectiveness of the Quarantine of Wuhan City Against the Corona Virus Disease 2019 (COVID-19): Well-Mixed SEIR Model Analysis | 10.1002/jmv.25827 | Original research | The study did not analyzed COVID-19 patients |
| Huang JF, et al. | Telestroke in the Time of COVID-19: The Mayo Clinic Experience | 10.1016/j.mayocp.2020.06.007 | Original research | The study did not analyzed COVID-19 patients |
| Huang Y, et al. | Treatment strategies of hospitalized patients with coronavirus disease-19 | 10.18632/aging.103370 | Original research | The study did not analyzed COVID-19 patients |
| Iglói Z, et al. | Comparison of commercial realtime reverse transcription PCR assays for the detection of SARS-CoV-2 | 10.1016/j.jcv.2020.104510 | Short communication | The study did not analyzed COVID-19 patients |
| Jiang Q, et al. | The Prevalence, Characteristics, and Related Factors of Pressure Injury in Medical Staff Wearing Personal Protective Equipment Against COVID-19 in China: A Multicentre Cross-Sectional Survey | 10.1111/iwj.13391 | Original research | The study did not analyzed COVID-19 patients |
| Jones NK, et al. | Effective control of SARS-CoV-2 transmission between healthcare workers during a period of diminished community prevalence of COVID-19 | 10.7554/eLife.59391 | Original research | The study did not analyzed COVID-19 patients |
| Khader Y, et al. | Dentists' Awareness, Perception, and Attitude Regarding COVID-19 and Infection Control: Cross-Sectional Study Among Jordanian Dentists | 10.2196/18798 | Original research | The study did not analyzed COVID-19 patients |
| Kim E, et al. | Microneedle Array Delivered Recombinant Coronavirus Vaccines: Immunogenicity and Rapid Translational Development | 10.1016/j.ebiom.2020.102743 | Original Research | The study did not analyzed COVID-19 patients |
| Kocyigit BF, et al. | YouTube as a source of information on COVID-19 and rheumatic disease link | 10.1007/s10067-020-05176-3 | Original research | The study did not analyzed COVID-19 patients |
| Kotian, et al. | Knowledge and understanding among medical imaging professionals in India during the rapid rise of the covid-19 pandemic | 10.1007/s12553-020-00437-2 | Original research | The study did not analyzed COVID-19 patients |
| Koziatek CA, et al. | Assessing the Impact of a Rapidly Scaled Virtual Urgent Care in New York City During the COVID-19 Pandemic | 10.1016/j.jemermed.2020.06.041 | Original research | The study did not analyzed COVID-19 patients |
| Lara B, et al. | Neuropsychiatric symptoms and quality of life in Spanish patients with Alzheimer's disease during the COVID-19 lockdown | 10.1111/ene.14339 | Short communication | The study did not analyzed COVID-19 patients |
| Li M, et al. | The SARS-CoV-2 Receptor ACE2 Expression of Maternal-Fetal Interface and Fetal Organs by Single-Cell Transcriptome Study | 10.1371/journal.pone.0230295 | Original research | The study did not analyzed COVID-19 patients |
| Li S, et al. | Internet Use, Risk Awareness, and Demographic Characteristics Associated With Engagement in Preventive Behaviors and Testing: Cross-Sectional Survey on COVID-19 in the United States | 10.2196/19782 | Original research | The study did not analyzed COVID-19 patients |
| Linton NM, et al. | Incubation Period and Other Epidemiological Characteristics of 2019 Novel Coronavirus Infections with Right Truncation: A Statistical Analysis of Publicly Available Case Data. | 10.3390/jcm9020538 | Original research | The study did not analyzed COVID-19 patients |
| Liu CY, et al. | The prevalence and influencing factors in anxiety in medical workers fighting COVID-19 in China: a cross-sectional survey | 10.1017/S0950268820001107 | Original research | The study did not analyzed COVID-19 patients |
| Loh TP, et al. | Laboratory practices to mitigate biohazard risks during the COVID-19 outbreak: an IFCC global survey | 10.1515/cclm-2020-0711 | Original Article | The study did not analyzed COVID-19 patients |
| Mackey T, et al. | Machine Learning to Detect Self-Reporting of Symptoms, Testing Access, and Recovery Associated With COVID-19 on Twitter: Retrospective Big Data Infoveillance Study | 10.2196/19509 | Original research | The study did not analyzed COVID-19 patients |
| Madanelo M, et al. | The impact of the coronavirus disease 2019 pandemic on the utilisation of emergency urological services | 10.1111/bju.15109 | Original research | The study did not analyzed COVID-19 patients |
| Massie AB, et al. | Identifying scenarios of benefit or harm from kidney transplantation during the COVID-19 pandemic: A stochastic simulation and machine learning study | 10.1111/ajt.16117 | Original research | The study did not analyzed COVID-19 patients |
| Mattioli AV, et al. | Quarantine during COVID-19 outbreak: Changes in diet and physical activity increase the risk of cardiovascular disease. | 10.1016/j.numecd.2020.05.020 | Viewpoint | The study did not analyzed COVID-19 patients |
| Monteagudo A, et al. | Continuous Intravenous Anakinra Infusion to Calm the Cytokine Storm in Macrophage Activation Syndrome | 10.1002/acr2.11135 | Original Research | The study did not analyzed COVID-19 patients |
| Nasrallah AA, et al. | A large number of COVID-19 interventional clinical trials were registered soon after the pandemic onset: a descriptive analysis | 10.1016/j.jclinepi.2020.06.005 | Original Article | The study did not analyzed COVID-19 patients |
| Ng SW, et al. | Cellular Metabolic Profiling of CrFK Cells Infected with Feline Infectious Peritonitis Virus Using Phenotype Microarrays | 10.3390/pathogens9050412 | Original research | The study did not analyzed COVID-19 patients |
| Nguyen HN, et al. | People With Suspected COVID-19 Symptoms Were More Likely Depressed and Had Lower Health-Related Quality of Life: The Potential Benefit of Health Literacy | 10.3390/jcm9040965 | Original research | The study did not analyzed COVID-19 patients |
| Nochaiwong S, et al. | Mental health circumstances among health care workers and general public under the pandemic situation of COVID-19 (HOME-COVID-19) | 10.1097/MD.0000000000020751 | Original research | The study did not analyzed COVID-19 patients |
| Ong DSY, et al. | Comparison of diagnostic accuracies of rapid serological tests and ELISA to molecular diagnostics in patients with suspected coronavirus disease 2019 presenting to the hospital | 10.1016/j.cmi.2020.05.028 | Original Article | The study did not analyzed COVID-19 patients |
| Petrarca L, et al. | Human bocavirus in children hospitalized for acute respiratory tract infection in Rome | 10.1007/s12519-019-00324-5 | Original research | The study did not analyzed COVID-19 patients |
| Szepietowski JC, et al. | Face Mask-induced Itch: A Self-questionnaire Study of 2,315 Responders During the COVID-19 Pandemic | 10.2340/00015555-3536 | Original research | The study did not analyzed COVID-19 patients |
| Tini G, et al. | Semantic and Geographical Analysis of COVID-19 Trials Reveals a Fragmented Clinical Research Landscape Likely to Impair Informativeness | 10.3389/fmed.2020.00367 | Original research | The study did not analyzed COVID-19 patients |
| Xiong Y, et al. | Transcriptomic Characteristics of Bronchoalveolar Lavage Fluid and Peripheral Blood Mononuclear Cells in COVID-19 Patients | 10.1080/22221751.2020.1747363 | Original Research | The study did not analyzed COVID-19 patients |
| Yip CC, et al. | Evaluation of the commercially available LightMix® Modular E-gene kit using clinical and proficiency testing specimens for SARS-CoV-2 detection | 10.1016/j.jcv.2020.104476 | Original research | The study did not analyzed COVID-19 patients |
| Zhang L, et al | How scientific research reacts to international public health emergencies: a global analysis of response patterns | 10.1007/s11192-020-03531-4 | Original research | The study did not analyzed COVID-19 patients |
| Zhao & Chen | Modeling the Epidemic Dynamics and Control of COVID-19 Outbreak in China | 10.1007/s40484-020-0199-0 | Original research | The study did not analyzed COVID-19 patients |
| Zhong B, et al. | Knowledge, Attitudes, and Practices Towards COVID-19 Among Chinese Residents During the Rapid Rise Period of the COVID-19 Outbreak: A Quick Online Cross-Sectional Survey | 10.7150/ijbs.45221 | Original research | The study did not analyzed COVID-19 patients |

**Additional Table 2. Included articles.**

| Citation | Title | DOI | Mention of any other respiratory disease | Number and % of COVID-19 patients with underlying respiratory disease | Mention of Asthma | | Number (%) COVID-19 patients with asthma | | Number of COVID-19 patients | |
| --- | --- | --- | --- | --- | --- | --- | --- | --- | --- | --- |
| Aggarwal S, et al. | Clinical features, laboratory characteristics, and outcomes of patients hospitalized with coronavirus disease 2019 (COVID-19): Early report from the United States | 10.1515/dx-2020-0046 | Yes | COPD: 2 (13%) | No | - | | 16 | |  |
| Ai JW, et al. | Characteristics of COVID-19 Patients With Gastrointestinal Symptoms: An Analysis of Seven Patients in China | 10.3389/fmed.2020.00308 | No | - | No | - | | 7 | |  |
| Akdur A, et al. | Coronavirus Disease (COVID-19) in Kidney and Liver Transplant Patients: A Single-Center Experience | 10.6002/ect.2020.0193 | No | - | No | - | | 583 | |  |
| Alberici F, et al. | A single center observational study of the clinical characteristics and short-term outcome of 20 kidney transplant patients admitted for SARS-CoV2 pneumonia | 10.1016/j.kint.2020.04.002 | No | - | No | - | | 20 | |  |
| Alkundi A, et al. | Clinical characteristics and outcomes of COVID-19 hospitalized patients with diabetes in the United Kingdom: A retrospective single centre study | 10.1016/j.diabres.2020.108263 | Yes | COPD: 7 (7.3%) | Yes | 6 (2.6%) | | 232 | |  |
| Alsofayan YM, et al. | Clinical characteristics of COVID-19 in Saudi Arabia: A national retrospective study | 10.1016/j.jiph.2020.05.026 | Yes | Chronic lung disease: 57 (5.2%) | Yes | 54 (4.9%) | | 1519 | |  |
| Aretz M, et al. | Characteristics and Outcomes of 21 Critically Ill Patients With COVID-19 in Washington State | 10.1001/jama.2020.4326 | Yes | COPD: 7 (33.3%) | Yes | 2 (9.1%) | | 21 | |  |
| Arslan H, et al. | Incidence and Immunologic Analysis of Coronavirus Disease (COVID-19) in Hemodialysis Patients:A Single-Center Experience | 10.6002/ect.2020.0194 | Yes | COPD: 1 (14%) | No | - | | 7 | |  |
| Asghar MS, et al. | Clinical Profiles, Characteristics, and Outcomes of the First 100 Admitted COVID-19 Patients in Pakistan: A Single-Center Retrospective Study in a Tertiary Care Hospital of Karachi | 10.7759/cureus.8712 | Yes | COPD: 3 (3%) | Yes | 2 (2%) | | 100 | |  |
| Bar S, et al. | The association of lung ultrasound images with COVID-19 infection in an emergency room cohort | 10.1111/anae.15175 | No | - | No | - | | 31 | |  |
| Barman HA, et al. | Prognostic significance of cardiac injury in COVID-19 patients with and without coronary artery disease | 10.1097/MCA.0000000000000914 | Yes | COPD: 73 (12%) | No | - | | 607 | |  |
| Becchetti C, et al. | COVID-19 in an international European liver transplant recipient cohort | 10.1136/gutjnl-2020-321923 | Yes | COPD: 7 (12%) | No | - | | 57 | |  |
| Bellosta R, et al. | Acute Limb Ischemia in Patients With COVID-19 Pneumonia | 10.1016/j.jvs.2020.04.483 | Yes | COPD: 2 (10%) | No | - | | 20 | |  |
| Benger M, et al. | Intracerebral haemorrhage and COVID-19: Clinical characteristics from a case series | 10.1016/j.bbi.2020.06.005 | No | - | Yes | 1 (20%) | | 5 | |  |
| Benussi A, et al. | Clinical characteristics and outcomes of inpatients with neurologic disease and COVID-19 in Brescia, Lombardy, Italy | 10.1212/WNL.0000000000009848 | No | - | No | - | | 173 | |  |
| Bezzio C, et al. | Outcomes of COVID-19 in 79 Patients With IBD in Italy: An IG-IBD Study | 10.1136/gutjnl-2020-321411 | Yes | COPD: 5 (6%) | No | - | | 79 | |  |
| Bhatla A, et al. | COVID-19 and cardiac arrhythmias | 10.1016/j.hrthm.2020.06.016 | Yes | COPD: 63 (9%) | No | - | | 700 | |  |
| Bhatraju PK, et al | Covid-19 in Critically Ill Patients in the Seattle Region — Case Series | 10.1056/NEJMoa2004500 | Yes | COPD: 1 (4%) | Yes | 3 (12.5%) | | 24 | |  |
| Bobin Mi, et al. | Characteristics and Early Prognosis of COVID-19 Infection in Fracture Patients | 10.2106/JBJS.20.00390 | Yes | COPD: 1 (10 %) | No | - | | 10 | |  |
| Biagi A, et al. | Clinical and epidemiological characteristics of 320 deceased patients with COVID-19 in an Italian Province: A retrospective observational study | 10.1002/jmv.26147 | Yes | COPD: 56 (17.5%) | No | - | | 320 | |  |
| Brill SE, et al. | COVID-19: a retrospective cohort study with focus on the over-80s and hospital-onset disease | 10.1186/s12916-020-01665-z | Yes | Respiratory condition: 85 (19%) | No | - | | 450 | |  |
| Cai Q, et al. | Characteristics of Liver Tests in COVID-19 Patients | 10.1016/j.jhep.2020.04.006 | No | - | No | - | | 417 | |  |
| Cai Q, et al. | COVID-19 in a Designated Infectious Diseases Hospital Outside Hubei Province, China | 10.1111/all.14309 | No | - | No | - | | 298 | |  |
| Cai Q, et al. | Obesity and COVID-19 Severity in a Designated Hospital in Shenzhen, China | 10.2337/dc20-0576 | Yes | COPD: 32 (8.35%) | No | - | | 383 | |  |
| Cai Y, et al. | Coronavirus Disease 2019 in the Perioperative Period of Lung Resection: A Brief Report From a Single Thoracic Surgery Department in Wuhan, People's Republic of China | 10.1016/j.jtho.2020.04.003 | Yes | COPD: 2 (28.5%) | No | - | | 7 | |  |
| Caly L, et al. | Isolation and Rapid Sharing of the 2019 Novel Coronavirus (SARS-CoV-2) From the First Patient Diagnosed With COVID-19 in Australia | 10.5694/mja2.50569 | No | - | No | - | | 1 | |  |
| Cao J, et al. | Clinical Features and Short-term Outcomes of 102 Patients With Corona Virus Disease 2019 in Wuhan, China | 10.1093/cid/ciaa243 | Yes | Respiratory Diseases: 9 (10.8%) | No | - | | 102 | |  |
| Cao Z, et al. | Clinical characteristics of Coronavirus Disease 2019 patients in Beijing, China | 10.1371/journal.pone.0234764 | Yes | COPD: 5 6.3%) | No | - | | 80 | |  |
| Capra R, et al. | Impact of Low Dose Tocilizumab on Mortality Rate in Patients With COVID-19 Related Pneumonia | 10.1016/j.ejim.2020.05.009 | No | - | No | - | | 85 | |  |
| Cariou B, et al. | Phenotypic characteristics and prognosis of inpatients with COVID-19 and diabetes: the CORONADO study | 10.1007/s00125-020-05180-x | Yes | COPD: 133 (10.4%) | No | - | | 1278 | |  |
| Cen Y, et al. | Risk factors for disease progression in patients with mild to moderate coronavirus disease 2019-a multi-centre observational study | 10.1016/j.cmi.2020.05.041 | Yes | COPD: 46 (4.6%) | No | - | | 1007 | |  |
| Challener DW, et al. | Screening for COVID-19: Patient factors predicting positive PCR test | 10.1017/ice.2020.249 | Yes | COPD: 6 (12.5%) | Yes | 6 (12.5%)* same patients with other chronic pulmonary diseases | | 48 | |  |
| Chao JY, et al. | Clinical Characteristics and Outcomes of Hospitalized and Critically Ill Children and Adolescents with Coronavirus Disease 2019 (COVID-19) at a Tertiary Care Medical Center in New York City | 10.1016/j.jpeds.2020.05.006 | No | - | Yes | 11 (24.4%) | | 46 | |  |
| Chen G, et al. | Clinical and Immunological Features of Severe and Moderate Coronavirus Disease 2019 | 10.1172/JCI137244 | No | - | No | - | | 41 | |  |
| Chen H, et al. | Clinical Characteristics and Intrauterine Vertical Transmission Potential of COVID-19 Infection in Nine Pregnant Women: A Retrospective Review of Medical Records | 10.1016/S0140-6736(20)30360-3 | No | - | No | - | | 9 | |  |
| Chen J, et al. | Clinical progression of patients with COVID-19 in Shanghai, China | 10.1016/j.jinf.2020.03.004 | Yes | Respiratory Diseases: 5 (2%) | No | - | | 249 | |  |
| Chen N, et al. | Epidemiological and Clinical Characteristics of 99 Cases of 2019 Novel Coronavirus Pneumonia in Wuhan, China: A Descriptive Study | 10.1016/S0140-6736(20)30211-7 | Yes | Respiratory Diseases: 1 (1%) | No | - | | 99 | |  |
| Chen P, et al. | Clinical and Demographic Characteristics of Cluster Cases and Sporadic Cases of Coronavirus Disease 2019 (COVID-19) in 141 Patients in the Main District of Chongqing, China, Between January and February 2020 | 10.12659/MSM.923985 | Yes | COPD: 17 (12%) | No | - | | 141 | |  |
| Chen Q, et al. | Clinical Characteristics of 145 Patients With Corona Virus Disease 2019 (COVID-19) in Taizhou, Zhejiang, China | 10.1007/s15010-020-01432-5 | Yes | COPD: 6 (4,13%) | No | - | | 145 | |  |
| Chen R, et al. | Safety and efficacy of different anesthetic regimens for parturients with COVID-19 undergoing Cesarean delivery: a case series of 17 patients | 10.1007/s12630-020-01630-7 | No | - | No | - | | 17 | |  |
| Chen T, et al. | Clinical characteristics and outcomes of older patients with coronavirus disease 2019 (COVID-19) in Wuhan, China (2019): a single-centered, retrospective study | 10.1093/gerona/glaa089 | Yes | COPD: 8 (3.9%) | No | - | | 203 | |  |
| Chen T, et al. | Clinical Characteristics of 113 Deceased Patients With Coronavirus Disease 2019: Retrospective Study | 10.1136/bmj.m1091 | Yes | Chronic lung diseases: 18 (7%) | No | - | | 274 | |  |
| Chen TY, et al. | COVID-19 pneumonia in kidney transplant recipients: Focus on immunosuppression management | 10.1111/tid.13378 | No | - | No | - | | 30 | |  |
| Chen X, et al. | Associations of clinical characteristics and treatment regimens with the duration of viral RNA shedding in patients with COVID-19 | 10.1016/j.ijid.2020.06.091 | Yes | Respiratory diseases: 9 (3.4%) | No | - | | 267 | |  |
| Chen X, et al. | Detectable Serum SARS-CoV-2 Viral Load (RNAaemia) Is Closely Correlated With Drastically Elevated Interleukin 6 (IL-6) Level in Critically Ill COVID-19 Patients | 10.1093/cid/ciaa449 | Yes | Pulmonary diseases: 2 (4.2%) | No | - | | 48 | |  |
| Chen Y, et al. | Clinical Characteristics and Outcomes of Type 2 Diabetes Patients Infected with COVID-19: A Retrospective Study | 10.1016/j.eng.2020.05.017 | Yes | COPD: 2 (1.0%) | No | - | | 208 | |  |
| Chen Y, et al. | Epidemiological analysis of the early 38 fatalities in Hubei, China, of the coronavirus disease 2019 | 10.7189/jogh-10-011004 | Yes | COPD: 1 (2.6%) | No | - | | 38 | |  |
| Chen Y, et al. | The Presence of SARS-CoV-2 RNA in Feces of COVID-19 Patients | 10.1002/jmv.25825 | Yes | COPD: 2 (4.76%) | No | - | | 42 | |  |
| Chen Y, et al. | Clinical Characteristics and Outcomes of Patients With Diabetes and COVID-19 in Association With Glucose-Lowering Medication. | 10.2337/dc20-0660 | Yes | Chronic lung disease: 22 (2.4%) | No | - | | 904 | |  |
| Chen Y, et al. | Impact of Fundamental Diseases on Patients With COVID-19 | 10.1017/dmp.2020.139 | Yes | Chronic bronchitis: 7 (21.2%) | No | - | | 50 | |  |
| Cheng FY, et al. | Using Machine Learning to Predict ICU Transfer in Hospitalized COVID-19 Patients | 10.3390/jcm9061668 | Yes | COPD: 219 (8.42%) | Yes | 219 (8.42%) | | 2599 | |  |
| Cheung ZB & Forsh DA | Early outcomes after hip fracture surgery in COVID-19 patients in New York City | 10.1016/j.jor.2020.06.003 | Yes | COPD: 1 (10%) | Yes | 2 (20%) | | 10 | |  |
| Chhiba KD, et al. | Prevalence and characterization of asthma in hospitalized and nonhospitalized patients with COVID-19 | 10.1016/j.jaci.2020.06.010 | Yes | COPD: 111 (7,27%) | Yes | 220 (14.2%) | | 1542 | |  |
| Choi MH, et al. | Clinical Characteristics and Disease Progression in Early-Stage COVID-19 Patients in South Korea | 10.3390/jcm9061959 | Yes | Chronic lung disease 17 (5.8%) | No | - | | 293 | |  |
| Chung SM, et al. | The Risk of Diabetes on Clinical Outcomes in Patients with Coronavirus Disease 2019: A Retrospective Cohort Study | 10.4093/dmj.2020.0105 | Yes | Chronic lung disease: 4 (3.6%) | No | - | | 110 | |  |
| Clark TW, et al. | Diagnostic accuracy of the FebriDx host response point- of-care test in patients hospitalised with suspected COVID-19 | 10.1016/j.jinf.2020.06.051 | Yes | Respiratory disease: 37 (31%) | No | - | | 118 | |  |
| Colombi D, et al. | Well-aerated Lung on Admitting Chest CT to Predict Adverse Outcome in COVID-19 Pneumonia | 10.1148/radiol.2020201433 | Yes | Pulmonary diseseases: 40 (17%) | No | - | | 236 | |  |
| Corbett RW, et al. | Epidemiology of COVID-19 in an Urban Dialysis Center | 10.1681/ASN.2020040534 | No | - | No | - | | 300 | |  |
| COVID-19 Investigation Team | Clinical and virologic characteristics of the first 12 patients with coronavirus disease 2019 (COVID-19) in the United States | 10.1038/s41591-020-0877-5 | Yes | COPD: 1 (8.3%) | No | - | | 12 | |  |
| Covino M, et al. | Clinical characteristics and prognostic factors in COVID-19 patients aged ≥80 years | 10.1111/ggi.13960 | Yes | COPD: 7 (10.1%) | No | - | | 69 | |  |
| De Luca G, et al. | GM-CSF blockade with mavrilimumab in severe COVID-19 pneumonia and systemic hyperinflammation: a single-centre, prospective cohort study | 10.1016/S2665-9913(20)30170-3 | No | - | No | - | | 39 | |  |
| Deiana G, et al. | Deaths in SARS-Cov-2 Positive Patients in Italy: The Influence of Underlying Health Conditions on Lethality | 10.3390/ijerph17124450 | Yes | Chronic lung disease: 22 (22.7%) | No | - | | 97 | |  |
| Deng L, et al. | Arbidol Combined With LPV/r Versus LPV/r Alone Against Corona Virus Disease 2019: A Retrospective Cohort Study | 10.1016/j.jinf.2020.03.002 | Yes | COPD: 1 (5.9 %) | No | - | | 33 | |  |
| Deng W, et al. | Positive results for patients with COVID-19 discharged form hospital in Chongqing, China | 10.1186/s12879-020-05151-y | Yes | COPD: 8 (13.1%) | No | - | | 61 | |  |
| Deng Y, et al. | Clinical characteristics of fatal and recovered cases of coronavirus disease 2019 in Wuhan, China: a retrospective study | 10.1097/CM9.0000000000000824 | Yes | Lung disease: 25 (11.1%) | No | - | | 225 | |  |
| Ding Q, et al. | The Clinical Characteristics of Pneumonia Patients Coinfected With 2019 Novel Coronavirus and Influenza Virus in Wuhan, China | 10.1002/jmv.25781 | No | - | No | - | | 5 | |  |
| Dong X, et al. | Eleven Faces of Coronavirus Disease 2019 | 10.1111/all.14289 | Yes | COPD: 1 (9%) | No | - | | 11 | |  |
| D'Silva KM, et al. | Clinical characteristics and outcomes of patients with coronavirus disease 2019 (COVID-19) and rheumatic disease: a comparative cohort study from a US 'hot spot' | 10.1136/annrheumdis-2020-217888 | Yes | COPD: 9 (5.7%) | Yes | 31 (19.8%) | | 156 | |  |
| Du H, et al. | Clinical characteristics of 182 pediatric COVID-19 patients with different severities and allergic status | 10.1111/all.14452 | No | - | Yes | 1 (2.3%) | | 43 | |  |
| Du X, et al. | Clinical features of hemodialysis patients with COVID-19: a single-center retrospective study on 32 patients | 10.1007/s10157-020-01904-w | No | - | No | - | | 32 | |  |
| Duan X, et al. | A retrospective study of the initial 25 COVID-19 patients in Luoyang, China | 10.1007/s11604-020-00988-4 | Yes | COPD: 1 (4%) | No | - | | 25 | |  |
| Duanmu Y, et al. | Characteristics of Emergency Department Patients With COVID-19 at a Single Site in Northern California: Clinical Observations and Public Health Implications | 10.1111/acem.14003 | Yes | COPD: 1 (10%) | yes | 10 (10%) | | 100 | |  |
| Dube GK, et al. | COVID-19 in pancreas transplant recipients | 10.1111/tid.13359 | No | - | No | - | | 4 | |  |
| Fan J, et al. | The epidemiology of reverse transmission of COVID-19 in Gansu Province, China. | 10.1016/j.tmaid.2020.101741 | Yes | COPD: N/A | Yes | N/A | | 37 | |  |
| Fan N, et al. | Imaging characteristics of initial chest computed tomography and clinical manifestations of patients with COVID-19 pneumonia | 10.1007/s11604-020-00973-x | Yes | Chronic bronchitis N/A | No | - | | 150 | |  |
| Feng Y, et al. | COVID-19 with Different Severities: A Multicenter Study of Clinical Features | 10.1002/oby.22832 | Yes | COPD: 22 (4.6%) | No | - | | 476 | |  |
| Ferguson J, et al. | Characteristics and Outcomes of Coronavirus Disease Patients under Nonsurge Conditions, Northern California, USA, March-April 2020 | 10.3201/eid2608.201776 | Yes | COPD: 10 (13.9%) | Yes | 10 (13.9%)* same patients with COPD | | 72 | |  |
| Fernandéz R, et al. | COVID-19 in Solid Organ Transplant Recipients: A Single-Center Case Series From Spain | 10.1111/ajt.15929 | No | - | Yes | 1 (5.55%) | | 18 | |  |
| Fontana F, et al. | SARS-CoV-2 infection in dialysis patients in northern Italy: a single-centre experience | 10.1093/ckj/sfaa084 | No | - | No | - | | 15 | |  |
| Galloway JB, et al. | A clinical risk score to identify patients with COVID-19 at high risk of critical care admission or death: An observational cohort study | 10.1016/j.jinf.2020.05.064 | Yes | Chronic lung disease: 234 (20.2%) | No | - | | 1157 | |  |
| Gan J, et al. | Leucocyte Subsets Effectively Predict the Clinical Outcome of Patients With COVID-19 Pneumonia: A Retrospective Case-Control Study | 10.3389/fpubh.2020.00299 | Yes | COPD: 6(6%) | No | - | | 95 | |  |
| Gao M, et al. | A Study on Infectivity of Asymptomatic SARS-CoV-2 Carriers | 10.1016/j.rmed.2020.106026 | No | - | No | - | | 35 | |  |
| Gao X, et al. | Improving the early diagnosis of suspected patients with COVID-19: a retrospective study of 106 patients | 10.3855/jidc.12992 | Yes | COPD: 4 (3.8%) | No | - | | 106 | |  |
| Gao Y, et al. | Diagnostic Utility of Clinical Laboratory Data Determinations for Patients With the Severe COVID-19 | 10.1002/jmv.25770 | Yes | COPD: 8 (18.6%) | No | - | | 43 | |  |
| Garassino MC, et al. | COVID-19 in patients with thoracic malignancies (TERAVOLT): first results of an international, registry-based, cohort study | 10.1016/S1470-2045(20)30314-4 | Yes | COPD: 51 (26%) | No | - | | 198 | |  |
| Garfinkle R, et al. | Development and Validation of a Clinical Risk Score for IntensiveCare Resource Utilization After Colon Cancer Surgery: a PracticalGuide to the Selection of Patients During COVID-19 | 10.1007/s11605-020-04665-9 | No | - | No | - | | 54893 | |  |
| Gayam V, et al. | Presenting characteristics, comorbidities, and outcomes of patients coinfected with COVID-19 and Mycoplasma pneumoniae in the USA | 10.1002/jmv.26026 | No | - | Yes | 2 (33.3%) | | 6 | |  |
| Giacomelli A, et al. | 30-day mortality in patients hospitalized with COVID-19 during the first wave of the Italian epidemic: A prospective cohort study | 10.1016/j.phrs.2020.104931 | No | - | No | - | | 233 | |  |
| Gianfrancesco M, et al. | Characteristics associated with hospitalisation for COVID-19 in people with rheumatic disease: data from the COVID-19 Global Rheumatology Alliance physician-reported registry | 10.1136/annrheumdis-2020-217871 | Yes | Chronic lung diseases 127 (21%)*** | Yes | 127 (21%)*** | | 600 | |  |
| Gidari A, et al. | Predictive value of National Early Warning Score 2 (NEWS2) for intensive care unit admission in patients with SARS-CoV-2 infection | 10.1080/23744235.2020.1784457 | Yes | Chronic lung disease 5 (7%) | No | - | | 68 | |  |
| Gold JAW, et al. | Characteristics and Clinical Outcomes of Adult Patients Hospitalized With COVID-19 - Georgia, March 2020 | 10.15585/mmwr.mm6918e1 | Yes | COPD: 16 (5.2%) | Yes | 32 (10.5%) | | 305 | |  |
| Goyal P, et al. | Clinical Characteristics of Covid-19 in New York City | 10.1056/NEJMc2010419 | Yes | COPD: 20 (5.1%) | Yes | 49 (12.5%) | | 393 | |  |
| Grasselli G, et al. | Baseline Characteristics and Outcomes of 1591 Patients Infected With SARS-CoV-2 Admitted to ICUs of the Lombardy Region, Italy | 10.1001/jama.2020.5394 | Yes | COPD: 42 (4%) | No | - | | 1591 | |  |
| Guan WJ, et al. | Clinical Characteristics of Coronavirus Disease 2019 in China | 10.1056/NEJMoa2002032 | Yes | COPD: 12 (1.1%) | No | - | | 1099 | |  |
| Haimovich A, et al. | Patient factors associated with SARS-CoV-2 in an admitted emergency department population | 10.1002/emp2.12145 | Yes | Pulmonary circulation disorders 62 (7.9%) | No | - | | 786 | |  |
| Han C, et al. | Digestive Symptoms in COVID-19 Patients With Mild Disease Severity: Clinical Presentation, Stool Viral RNA Testing, and Outcomes | 10.14309/ajg.0000000000000664 | Yes | Chronic lung disease: 8 (3.9%) | No | - | | 206 | |  |
| Han J, et al. | Analysis of factors affecting the prognosis of COVID-19 patients and viral shedding duration | 10.1017/S0950268820001399 | No | - | No | - | | 185 | |  |
| Han Y, et al. | A comparative‐descriptive analysis of clinical characteristics in 2019‐coronavirus‐infected children and adults | 10.1002/jmv.25835 | No | - | No | - | | 32 | |  |
| He S, et al. | Relationship between chest CT manifestations and immune response in COVID-19 patients | 10.1016/j.ijid.2020.06.059 | Yes | COPD: 2 (2%) | No | - | | 93 | |  |
| Ho HE, et al. | Clinical outcomes and immunologic characteristics of Covid-19 in people with HIV | 10.1093/infdis/jiaa380 | Yes | Lung disease, asthma, or COPD 25 (26.9%) | Yes | 25 (26.9%)* same patients with other lung diseases | | 93 | |  |
| Hong KS, et al. | Clinical Features and Outcomes of 98 Patients Hospitalized With SARS-CoV-2 Infection in Daegu, South Korea: A Brief Descriptive Study | 10.3349/ymj.2020.61.5.431 | Yes | COPD: 5 (3.8%) | No | - | | 98 | |  |
| Hu H, et al. | Comparing Rapid Scoring Systems in Mortality Prediction of Critically Ill Patients With Novel Coronavirus Disease | 10.1111/acem.13992 | Yes | Chronic pulmonary disease: 12 (11.4%) | No | - | | 105 | |  |
| Hu Z, et al. | Clinical Characteristics of 24 Asymptomatic Infections With COVID-19 Screened Among Close Contacts in Nanjing, China | 10.1007/s11427-020-1661-4 | No | - | No | - | | 24 | |  |
| Huang C, et al. | Clinical features of patients infected with 2019 novel coronavirus in Wuhan, China | 10.1016/S0140-6736(20)30183-5 | Yes | COPD: 1 (2%) | No | - | | 41 | |  |
| Huang C, et al. | Mining the Characteristics of COVID-19 Patients in China: Analysis of Social Media Posts | 10.2196/19087 | Yes | COPD: 10 (2.06%) | No | - | | 485 | |  |
| Huang D, et al. | A novel risk score to predict diagnosis with coronavirus disease 2019 (COVID-19) in suspected patients: A retrospective, multicenter, and observational study | 10.1002/jmv.26143 | Yes | COPD: 9 (2.7%) | Yes | 5 (1.5%) | | 336 | |  |
| Huang L, et al. | Rapid asymptomatic transmission of COVID-19 during the incubation period demonstrating strong infectivity in a cluster of youngsters aged 16-23 years outside Wuhan and characteristics of young patients with COVID-19: A prospective contact-tracing study | 10.1016/j.jinf.2020.03.006 | No | - | No | - | | 8 | |  |
| Huang M, et al. | Clinical Characteristics and Predictors of Disease Progression in Severe Patients with COVID-19 Infection in Jiangsu Province, China: A Descriptive Study | 10.1016/j.amjms.2020.05.038 | Yes | COPD: 2 (3.3%) | No | - | | 60 | |  |
| Huang Q, et al. | Clinical characteristics and drug therapies in patients with the common-type coronavirus disease 2019 in Hunan, China | 10.1007/s11096-020-01031-2 | Yes | Chronic bronchitis: 2 (3.7%) | No | - | | 54 | |  |
| Huang R, et al. | Clinical Findings of Patients With Coronavirus Disease 2019 in Jiangsu Province, China: A Retrospective, Multi-Center Study | 10.1371/journal.pntd.0008280 | Yes | Chronic lung disease: 7 (3.5%) | No | - | | 202 | |  |
| Huang Y, et al. | Clinical characteristics of 17 patients with COVID-19 and systemic autoimmune diseases: a retrospective study | 10.1136/annrheumdis-2020-217425 | Yes | Respiratory disease: 6 (4%) | No | - | | 17 | |  |
| Huang Y, et al. | Clinical Characteristics of Laboratory Confirmed Positive Cases of SARS-CoV-2 Infection in Wuhan, China: A Retrospective Single Center Analysis | 10.1016/j.tmaid.2020.101606 | Yes | COPD: 3 (8.82%) | No | - | | 34 | |  |
| Hur K, et al. | Factors Associated With Intubation and Prolonged Intubation in Hospitalized Patients With COVID-19 | 10.1177/0194599820929640 | Yes | Pulmonary disease: 78 (16%) | No | - | | 486 | |  |
| Ihle-Hansen H, et al. | COVID-19: Symptoms, Course of Illness and Use of Clinical Scoring Systems for the First 42 Patients Admitted to a Norwegian Local Hospital | 10.4045/tidsskr.20.0301 | Yes | COPD: 3 (7%) | No | - | | 42 | |  |
| Ikitimur H, et al. | Determining host factors contributing to disease severity in a family cluster of 29 hospitalized SARS-CoV-2 patients: Could genetic factors be relevant in the clinical course of COVID-19? | 10.1002/jmv.26106 | Yes | Chronic respiratory disease, COPD, and asthma bronchiale 19 (23.45%) | Yes | 19 (23.45%)* same patients with other respiratory illness | | 81 | |  |
| Inciardi RM, et al. | Characteristics and Outcomes of Patients Hospitalized for COVID-19 and Cardiac Disease in Northern Italy | 10.1093/eurheartj/ehaa388 | Yes | COPD: 9 (9%) | No | - | | 99 | |  |
| Jang JG, et al. | Prognostic Accuracy of the SIRS, qSOFA, and NEWS for Early Detection of Clinical Deterioration in SARS-CoV-2 Infected Patients | 10.3346/jkms.2020.35.e234 | Yes | Chronic lung disease 4 (3.6%) | No | - | | 110 | |  |
| Javanian M, et al. | Clinical and Laboratory Findings From Patients With COVID-19 Pneumonia in Babol North of Iran: A Retrospective Cohort Study | 10.2478/rjim-2020-0013 | Yes | COPD: 12 (12%) | No | - | | 100 | |  |
| Jehi L, et al. | Individualizing Risk Prediction for Positive Coronavirus Disease 2019 Testing: Results From 11,672 Patients | 10.1016/j.chest.2020.05.580 | Yes | COPD/emphysema: 14 (1.26%) | Yes | 163 (14.7%) | | 1108 | |  |
| Ji M, et al. | Characteristics of Disease Progress in Patients With Coronavirus Disease 2019 in Wuhan, China | 10.1017/S0950268820000977 | Yes | COPD: 2 (2%) | No | - | | 101 | |  |
| Jie B, et al. | Clinical and Dynamic Computed Tomography Features of 24 Patients With Coronavirus Disease 2019 | 10.1177/0846537120918834 | Yes | Pulmonary tuberculosis 1 (4.16%) | No | - | | 24 | |  |
| Jin A, et al. | Clinical characteristics of patients diagnosed with COVID-19 in Beijing | 10.1016/j.bsheal.2020.05.003 | Yes | COPD: 3 (7%) | No | - | | 45 | |  |
| Jin X, et al. | Epidemiological, clinical and virological characteristics of 74 cases of coronavirus-infected disease 2019 (COVID-19) with gastrointestinal symptoms | 10.1136/gutjnl-2020-320926 | Yes | COPD: 1 (0.15%) | No | - | | 651 | |  |
| Kant A, et al. | The relationship between diagnostic value of chest computed tomography imaging and symptom duration in COVID infection | 10.4103/atm.ATM_165_20 | No | - | No | - | | 105 | |  |
| Kanthimathinathan HK, et al. | COVID-19: A UK Children's Hospital Experience | 10.1542/hpeds.2020-000208 | Yes | Respiratory disease: 6 (13%) | No | - | | 45 | |  |
| Kaushik S, et al. | Multisystem Inflammatory Syndrome in Children Associated with Severe Acute Respiratory Syndrome Coronavirus 2 Infection (MIS-C): A Multi-institutional Study from New York City | 10.1016/j.jpeds.2020.06.045 | No | - | Yes | 5 (15%) | | 33 | |  |
| Ketcham SW, et al. | Coronavirus Disease-2019 in Heart Transplant Recipients in Southeastern Michigan: A Case Series | 10.1016/j.cardfail.2020.05.008 | Yes | Obstructive sleep apnea: 6 (46%) | No | - | | 13 | |  |
| Khamis F, et al. | Clinical characteristics and outcomes of the first 63 adult patients hospitalized with COVID-19: An experience from Oman | 10.1016/j.jiph.2020.06.002 | No | - | No | - | | 63 | |  |
| Khider L, et al. | Curative anticoagulation prevents endothelial lesion in COVID-19 patients | 10.1111/jth.14968 | No | - | No | - | | 66 | |  |
| Killerby et al. | Characteristics Associated with Hospitalization Among Patients with COVID-19 | 10.15585/mmwr.mm6925e1 | Yes | Chronic respiratory disease: 101 (19%) | No | - | | 531 | |  |
| Knight M, et al. | Characteristics and outcomes of pregnant women admitted to hospital with confirmed SARS-CoV-2 infection in UK: national population based cohort study | 10.1136/bmj.m2107 | No | - | Yes | 31 (7%) | | 427 | |  |
| Koleilat I, et al. | Clinical characteristics of acute lower extremity deep venous thrombosis diagnosed by duplex in patients hospitalized for coronavirus disease 2019 | 10.1016/j.jvsv.2020.06.012 | Yes | COPD: 13 (11.1%) | No | - | | 117 | |  |
| Kong W, et al. | Comparison of clinical and epidemiological characteristics of asymptomatic and symptomatic SARS-CoV-2 infection: A multi-center study in Sichuan Province, China | 10.1016/j.tmaid.2020.101754 | Yes | Pulmonary tuberculosis: 3 (3%) | No | - | | 100 | |  |
| Kong Y, et al. | VEGF-D: a novel biomarker for detection of COVID-19 progression | 10.1186/s13054-020-03079-y | Yes | Chronic Pulmonary disease: 6 (25%) | No | - | | 24 | |  |
| Korkmaz MF, et al. | The Epidemiological and Clinical Characteristics of 81 Children with COVID-19 in a Pandemic Hospital in Turkey: an Observational Cohort Study | 10.3346/jkms.2020.35.e236 | No | - | Yes | 1 (1,23%) | | 81 | |  |
| Lagadinou M, et al. | Prognosis of COVID-19: Changes in laboratory parameters | 10.1186/s12931-020-01428-7 | Yes | Acute respiratory distress syndrome: 35 (10.00%) | No | - | | 349 | |  |
| Lam RPK, et al. | Clinical, laboratory, and radiological features indicative of novel coronavirus disease (COVID-19) in emergency departments - a multicentre case-control study in Hong Kong. | 10.1002/emp2.12183 | Yes | COPD: 1 (2.5%) | No | - | | 37 | |  |
| Lechien JR, et al. | Clinical and Epidemiological Characteristics of 1,420 European Patients With Mild-To-Moderate Coronavirus Disease 2019 | 10.1111/joim.13089 | Yes | Respiratory insufficiency 10 (0.7%) | Yes | 93 (6.5%) | | 1420 | |  |
| Lee J, et al. | Early Intervention of Palliative Care in the Emergency Department During the COVID-19 Pandemic | 10.1001/jamainternmed.2020.2713 | Yes | Chronic lung condition 20 (18,2%) | No | - | | 110 | |  |
| Lee JY, et al. | Risk Factors for Mortality and Respiratory Support in Elderly Patients Hospitalized with COVID-19 in Korea | 10.3346/jkms.2020.35.e223 | Yes | Chronic lung disease: 8 (8,2%) | No | - | | 98 | |  |
| Lee LY, et al. | COVID-19 mortality in patients with cancer on chemotherapy or other anticancer treatments: a prospective cohort study | 10.1016/S0140-6736(20)31173-9 | Yes | COPD: 61 (8%) | No | - | | 800 | |  |
| Lei S, et al. | Clinical Characteristics and Outcomes of Patients Undergoing Surgeries During the Incubation Period of COVID-19 Infection | 10.1016/j.eclinm.2020.100331 | Yes | COPD: 1 (2.9%) | No | - | | 34 | |  |
| Lei Z, et al. | A Cross-Sectional Comparison of Epidemiological and Clinical Features of Patients With Coronavirus Disease (COVID-19) in Wuhan and Outside Wuhan, China | 10.1016/j.tmaid.2020.101664 | Yes | COPD: 2 (1.68%) | No | - | | 119 | |  |
| Li J, et al. | Clinical Characteristics and Outcomes of 74 Patients With Severe or Critical COVID-19 | 10.1016/j.amjms.2020.05.040 | Yes | Tuberculosis: 6 (8.1%) | No | - | | 74 | |  |
| Li J, et al. | Clinical features of familial clustering in patients infected with 2019 novel coronavirus in Wuhan, China | 10.1016/j.virusres.2020.198043 | No | - | No | - | | 94 | |  |
| Li K, et al. | The Clinical and Chest CT Features Associated With Severe and Critical COVID-19 Pneumonia | 10.1097/RLI.0000000000000672 | Yes | COPD: 5 (6%) | No | - | | 83 | |  |
| Li M, et al. | Cardiovascular disease potentially contributes to the progression and poor prognosis of COVID-19 | 10.1016/j.numecd.2020.04.013 | No | - | No | - | | 83 | |  |
| Li N, et al. | Maternal and Neonatal Outcomes of Pregnant Women With COVID-19 Pneumonia: A Case-Control Study | 10.1093/cid/ciaa352 | No | - | No | - | | 16 | |  |
| Li S, et al. | Clinical and pathological investigation of patients with severe COVID-19 | 10.1172/jci.insight.138070 | Yes | COPD: 7 (10.1%) | No | - | | 69 | |  |
| Li X, et al. | Risk factors for severity and mortality in adult COVID-19 inpatients in Wuhan | 10.1016/j.jaci.2020.04.006 | Yes | COPD: 17 (3.1%) | Yes | 5 (0.9%) | | 548 | |  |
| Li X, et al. | Clinical and CT characteristics which indicate timely radiological reexamination in patients with COVID-19: A retrospective study in Beijing, China | 10.1016/j.jrid.2020.05.003 | Yes | COPD: 2 (2.7%) | No | - | | 73 | |  |
| Li X, et al. | Effect of combination antiviral therapy on hematological profiles in 151 adults hospitalized with severe coronavirus disease 2019 | 10.1016/j.phrs.2020.105036 | Yes | Respiratory disease: 6 (3,97%) | No | - | | 151 | |  |
| Li X, et al. | Clinical characteristics of 25 death cases with COVID-19: a retrospective review of medical records in a single medical center, Wuhan, China | 10.1016/j.ijid.2020.03.053 | Yes | COPD: 2 (8%) | No | - | | 25 | |  |
| Li Y, et al. | Clinical and Transmission Characteristics of Covid-19 - A Retrospective Study of 25 Cases From a Single Thoracic Surgery Department | 10.1007/s11596-020-2176-2 | Yes | COPD: 5 (20%) | No | - | | 25 | |  |
| Li Y, et al. | Retrospective Analysis of Laboratory Testing in 54 Patients With Severe- Or Critical-Type 2019 Novel Coronavirus Pneumonia | 10.1038/s41374-020-0431-6 | Yes | Pulmonary disease: 4 (13,3%) | No | - | | 30 | |  |
| Liabeuf S, et al. | Association between renin-angiotensin system inhibitors and COVID-19 complications | 10.1093/ehjcvp/pvaa062 | Yes | COPD: 26 (10%) | Yes | 14 (5%) | | 268 | |  |
| Lian J, et al. | Epidemiological, clinical, and virological characteristics of 465 hospitalized cases of coronavirus disease 2019 (COVID-19) from Zhejiang province in China | 10.1111/irv.12758 | Yes | COPD: 0 (0%) | No | - | | 465 | |  |
| Lian J, et al. | Analysis of Epidemiological and Clinical Features in Older Patients With Corona Virus Disease 2019 (COVID-19) Out of Wuhan | 10.1093/cid/ciaa242 | Yes | COPD: 3 (0.38%) | No | - | | 788 | |  |
| Lian N, et al. | Umifenovir treatment is not associated with improved outcomes in patients with coronavirus disease 2019: a retrospective study | 10.1016/j.cmi.2020.04.026 | No | - | No | - | | 81 | |  |
| Liang W, et al. | Clinical Characteristics and Outcomes of Hospitalised Patients With COVID-19 Treated in Hubei (Epicenter) and Outside Hubei (Non-Epicenter): A Nationwide Analysis of China | 10.1183/13993003.00562-2020 | Yes | COPD: 24 (1.5%) | No | - | | 1590 | |  |
| Liang W, et al. | Development and Validation of a Clinical Risk Score to Predict the Occurrence of Critical Illness in Hospitalized Patients With COVID-19 | 10.1001/jamainternmed.2020.2033 | Yes | COPD: 24 (1.5%) | No | - | | 1590 | |  |
| Liang Y, et al. | Neurosensory dysfunction: A diagnostic marker of early COVID-19 | 10.1016/j.ijid.2020.06.086 | No | - | No | - | | 86 | |  |
| Liu BM, et al. | Epidemiological characteristics of COVID-19 patients in convalescence period | 10.1017/S0950268820001181 | Yes | Pulmonary tuberculosis: 4 (5.9%) | Yes | 1 (1.5%) | | 68 | |  |
| Liu D, et al. | The pulmonary sequalae in discharged patients with COVID-19: a short-term observational study | 10.1186/s12931-020-01385-1 | No | - | Yes | 4 (2.8%) | | 149 | |  |
| Liu F, et al. | Patients of COVID-19 may benefit from sustained Lopinavir-combined regimen and the increase of Eosinophil may predict the outcome of COVID-19 progression | 10.1016/j.ijid.2020.03.013 | No | - | No | - | | 10 | |  |
| Liu F, et al. | Prognostic value of interleukin-6, C-reactive protein, and procalcitonin in patients with COVID-19 | 10.1016/j.jcv.2020.104370 | Yes | Respiratory disease: 16 (11.4 %) | No | - | | 140 | |  |
| Liu J, et al. | Prevalence and predictive value of hypocalcemia in severe COVID-19 patients | 10.1016/j.jiph.2020.05.029 | Yes | Respiratory diseases: 11(10%) | No | - | | 107 | |  |
| Liu J, et al. | Neutrophil-to-lymphocyte ratio predicts critical illness patients with 2019 coronavirus disease in the early stage | 10.1186/s12967-020-02374-0 | Yes | COPD: 6 (5.2%) | No | - | | 115 | |  |
| Liu K, et al. | Clinical Characteristics of Novel Coronavirus Cases in Tertiary Hospitals in Hubei Province | 10.1097/CM9.0000000000000744 | Yes | COPD: 2 (1.5%) | No | - | | 137 | |  |
| Liu L, et al. | Epidemiological and Clinical Characteristics of Patients With Coronavirus Disease-2019 in Shiyan City, China | 10.3389/fcimb.2020.00284 | Yes | COPD: 8 (15.1%) | No | - | | 53 | |  |
| Liu M, et al. | COVID-19 Pneumonia: CT Findings of 122 Patients and Differentiation From Influenza Pneumonia | 10.1007/s00330-020-06928-0 | Yes | COPD: 4 (3%) | No | - | | 122 | |  |
| Liu SF, et al. | Comparison of the Characteristics and Outcomes of Coronavirus Disease 2019 in Different Types of Family Infections in Taiwan | 10.3390/jcm9051527 | No | - | No | - | | 32 | |  |
| Liu W, et al. | Analysis of factors associated with disease outcomes in hospitalized patients with 2019 novel coronavirus disease | 10.1097/CM9.0000000000000775 | Yes | COPD: 2 (2.6%) | No | - | | 78 | |  |
| Liu X, et al. | Analysis of clinical features and early warning signs in patients with severe COVID-19: A retrospective cohort study | 10.1371/journal.pone.0235459 | Yes | Respiratory disease 10 (9.6%) | No | - | | 104 | |  |
| Liu Y, et al. | Clinical and Biochemical Indexes From 2019-nCoV Infected Patients Linked to Viral Loads and Lung Injury | 10.1007/s11427-020-1643-8 | Yes | Chronic lung disease: 1 (8.3%) | No | - | | 12 | |  |
| Liu Y, et al. | Neutrophil-to-lymphocyte Ratio as an Independent Risk Factor for Mortality in Hospitalized Patients With COVID-19 | 10.1016/j.jinf.2020.04.002 | Yes | COPD: 8 (3.26%) | No | - | | 245 | |  |
| Liu Z, et al. | Association between Initial Chest CT or Clinical Features and Clinical Course in Patients with Coronavirus Disease 2019 Pneumonia | 10.3348/kjr.2020.0171 | No | - | No | - | | 72 | |  |
| Lo IL, et al. | Evaluation of SARS-CoV-2 RNA shedding in clinical specimens and clinical characteristics of 10 patients with COVID-19 in Macau | 10.7150/ijbs.45357 | No | - | No | - | | 7 | |  |
| Lodigiani C, et al. | Venous and Arterial Thromboembolic Complications in COVID-19 Patients Admitted to an Academic Hospital in Milan, Italy | 10.1016/j.thromres.2020.04.024 | Yes | COPD: 35 (9%) | No | - | | 388 | |  |
| Lokken EM, et al. | Clinical characteristics of 46 pregnant women with a severe acute respiratory syndrome coronavirus 2 infection in Washington State | 10.1016/j.ajog.2020.05.031 | No | - | Yes | 4 (8.7%) | | 46 | |  |
| Lorente-Ros A, et al. | Myocardial injury determination improves risk stratification and predicts mortality in COVID-19 patients | 10.5603/CJ.a2020.0089 | Yes | COPD: 100 (10,74%) | No | - | | 931 | |  |
| Louapre C, et al. | Clinical Characteristics and Outcomes in Patients With Coronavirus Disease 2019 and Multiple Sclerosis | 10.1001/jamaneurol.2020.2581 | Yes | Pulmonary comorbidity 17 (2.5%) | No | - | | 347 | |  |
| Lu R, et al. | Epidemiological and clinical characteristics of COVID-19 patients in Nantong, China | 10.3855/jidc.12678 | Yes | Respiratory system disease: 2 (7.1%) | No | - | | 28 | |  |
| Luo P, et al. | Metformin Treatment Was Associated with Decreased Mortality in COVID-19 Patients with Diabetes in a Retrospective Analysis | 10.4269/ajtmh.20-0375 | Yes | COPD: 6 (3.4%) | No | - | | 179 | |  |
| Luo X, et al. | Prognostic value of C-reactive protein in patients with COVID-19 | 10.1093/cid/ciaa641 | Yes | Chronic pulmonary disease: 23 (7.7%) | No | - | | 298 | |  |
| Ma S, et al. | Clinical characteristics of critically ill patients co-infected with SARS-CoV-2 and the influenza virus in Wuhan, China | 10.1016/j.ijid.2020.05.068 | Yes | Chronic pulmonary disease: 8 (8.6%) | No | - | | 93 | |  |
| Magagnoli et al. | Outcomes of Hydroxychloroquine Usage in United States Veterans Hospitalized with COVID-19 | 10.1016/j.medj.2020.06.001 | Yes | COPD: 175 (21.68%) | 40 | 40 (4.95%) | | 807 | |  |
| McCullough SA, et al. | Electrocardiographic Findings in Coronavirus Disease-19: Insights on Mortality and Underlying Myocardial Processes | 10.1016/j.cardfail.2020.06.005 | Yes | Respiratory disease: 142 (18.8%) | No | - | | 756 | |  |
| Melis D, et al. | Clinical efficacy, speed of improvement and safety of apremilast for the treatment of adult Psoriasis during COVID-19 pandemic | 10.1111/dth.13722 | No | - | No | - | | 48 | |  |
| Meng H, et al. | CT Imaging and Clinical Course of Asymptomatic Cases With COVID-19 Pneumonia at Admission in Wuhan, China | 10.1016/j.jinf.2020.04.004 | Yes | COPD: 1 (1.7%) | No | - | | 58 | |  |
| Meng Y, et al. | Sex-specific Clinical Characteristics and Prognosis of Coronavirus disease-19 Infection in Wuhan, China: A Retrospective Study of 168 Severe Patients | 10.1371/journal.ppat.1008520 | Yes | COPD: 1 (0.6%) | No | - | | 168 | |  |
| Merza MA, et al. | COVID-19 outbreak in Iraqi Kurdistan: The first report characterizing epidemiological, clinical, laboratory, and radiological findings of the disease. | 10.1016/j.dsx.2020.04.047 | Yes | Bronchitis: 2 (13.3%) | Yes | 2 (13.3%)* same patients with bronchitis | | 15 | |  |
| Mestre-Goméz B, et al. | Incidence of pulmonary embolism in non‑critically ill COVID‑19 patients. Predicting factors for a challenging diagnosis | 10.1007/s11239-020-02190-9 | Yes | Chronic obstrutive lung disease: 13 (14.28%) | Yes | 7 (7.69%) | | 91 | |  |
| Myers LC, et al. | Characteristics of Hospitalized Adults With COVID-19 in an Integrated Health Care System in California | 10.1001/jama.2020.7202 | Yes | COPD: 28 (7.4%) | Yes | 28 (7.4%)* same patients with COPD | | 377 | |  |
| Mikami T, et al. | Risk Factors for Mortality in Patients with COVID-19 in New York City. | 10.1007/s11606-020-05983-z | Yes | COPD: 176 (2.7%) | Yes | 271 (4.2%) | | 6493 | |  |
| Mitra AR, et al. | Baseline characteristics and outcomes of patients with COVID-19 admitted to intensive care units in Vancouver, Canada: a case series | 10.1503/cmaj.200794 | Yes | COPD: 8 (6.8%) | Yes | 14 (12%) | | 117 | |  |
| Mo P, et al. | Clinical characteristics of refractory COVID-19 pneumonia in Wuhan, China | 10.1093/cid/ciaa270 | Yes | COPD: 5 (3.2%) | No | - | | 155 | |  |
| Morena V, et al. | Off-label use of tocilizumab for the treatment of SARS-CoV-2 pneumonia in Milan, Italy | 10.1016/j.ejim.2020.05.011 | Yes | Chronic lung diseases: 5 (9.8%) | No | - | | 51 | |  |
| Myers CN, et al. | COVID-19 in lung transplant recipients | 10.1111/tid.13364 | Yes | COPD: 2 (25%) | No | - | | 8 | |  |
| Neveu S, et al. | Incidental diagnosis of Covid-19 pneumonia on chest computed tomography | 10.1016/j.diii.2020.05.011 | Yes | COPD: 1 (16,6%) | No | - | | 6 | |  |
| Nowak B, et al. | Clinical characteristics and short-term outcomes of patients with coronavirus disease 2019: a retrospective single-center experience of a designated hospital in Poland | 10.20452/pamw.15361 | Yes | COPD: 22 (13%) | No | - | | 169 | |  |
| Nie Y, et al. | Epidemiological and clinical characteristics of 671 COVID-19 patients in Henan Province, China | 10.1093/ije/dyaa081 | Yes | Respiratory disease: 15 (4.3%) | Yes | 15 (4,3%)* same patients with other respiratory diseases | | 671 | |  |
| Oualha M, et al. | Severe and fatal forms of COVID-19 in children | 10.1016/j.arcped.2020.05.010 | Yes | COPD: 1 (3.7%) e Chronic lung disease: 2 (7.4%) | Yes | 1 (3,7%) | | 27 | |  |
| Pan Lei, et al. | Clinical Characteristics of COVID-19 Patients With Digestive Symptoms in Hubei, China | 10.14309/ajg.0000000000000620 | Yes | Respiratory diseases: 9 (4.41%) | No | - | | 204 | |  |
| Pare JR, et al. | Point-of-care Lung Ultrasound Is More Sensitive than Chest Radiograph for Evaluation of COVID-19 | 10.5811/westjem.2020.5.47743 | Yes | COPD: 1 (3.7%) | Yes | 4 (14.8%) | | 27 | |  |
| Parri N, et al. | Characteristic of COVID-19 infection in pediatric patients: early findings from two Italian Pediatric Research Networks | 10.1007/s00431-020-03683-8 | Yes | Respiratory chronic diseases 16 (12%) | No | - | | 130 | |  |
| Pei G, et al. | Renal Involvement and Early Prognosis in Patients with COVID-19 Pneumonia | 10.1681/ASN.2020030276 | No | - | No | - | | 333 | |  |
| Pereira MR, et al. | COVID-19 in Solid Organ Transplant Recipients: Initial Report From the US Epicenter | 10.1111/ajt.15941 | Yes | Chronic lung disease: 17 (19%) | No | - | | 90 | |  |
| Petrilli CM, et al. | Factors associated with hospital admission and critical illness among 5279 people with coronavirus disease 2019 in New York City: prospective cohort study | 10.1136/bmj.m1966 | Yes | COPD: 906 (17.1%) | Yes | 906 (17.1%)* same patients with COPD | | 5279 | |  |
| Peyrony O, et al. | Accuracy of Emergency Department Clinical Findings for Diagnosis of Coronavirus Disease 2019 | 10.1016/j.annemergmed.2020.05.022 | Yes | COPD: 24 (6.2%) | Yes | 22 (5.7%) | | 391 | |  |
| Philips K, et al. | Rapid Implementation of an Adult COVID-19 Unit in a Children's Hospital | 10.1016/j.jpeds.2020.04.060 | Yes | History of lung disease: 19 (19) | No | - | | 100 | |  |
| Phipps MM, et al. | Acute Liver Injury in COVID-19: Prevalence and Association with Clinical Outcomes in a Large US Cohort | 10.1002/hep.31404 | Yes | COPD: 185 (8.1%) | Yes | 308 (14) | | 2273 | |  |
| Piano S, et al. | Abnormal liver function tests predict transfer to intensive care unit and death in COVID-19 | 10.1111/liv.14565 | Yes | COPD: 45 (8%) | No | - | | 565 | |  |
| Pisapia R, et al. | Differences among confirmed and not-confirmed COVID-19 patients at "D.Cotugno" hospital, Naples (Italy): what we learned from first suspected cases? | PMID: 32532943 | No | - | No | - | | 17 | |  |
| Pongpirul WA, et al. | Clinical Characteristics of Patients Hospitalized with Coronavirus Disease, Thailand | 10.3201/eid2607.200598 | Yes | COPD: 0 | 0 | 0 | | 11 | |  |
| Price-Haywood EG, et al. | Hospitalization and Mortality among Black Patients and White Patients with Covid-19 | 10.1056/NEJMsa2011686 | Yes | COPD: 79 (2.25%) | Yes | 147 (4%) | | 3481 | |  |
| Pung R, et al. | Investigation of three clusters of COVID-19 in Singapore: implications for surveillance and response measures | 10.1016/S0140-6736(20)30528-6 | No | - | No | - | | 17 | |  |
| Qi X, et al. | Clinical course of COVID-19 in patients with pre-existing decompensated cirrhosis: initial report from China | 10.1007/s12072-020-10051-z | No | - | No | - | | 3 | |  |
| Qin C, et al. | Clinical Characteristics and Outcomes of COVID-19 Patients With a History of Stroke in Wuhan, China | 10.1161/STROKEAHA.120.030365 | Yes | COPD: 28 (1.5) | No | - | | 1875 | |  |
| Qiu C, et al. | Transmission and Clinical Characteristics of Coronavirus Disease 2019 in 104 outside-Wuhan Patients, China | 10.1002/jmv.25975 | Yes | COPD: 1 (0.96%) | No | - | | 104 | |  |
| Qiu L, et al. | SARS-CoV-2 Is Not Detectable in the Vaginal Fluid of Women With Severe COVID-19 Infection | 10.1093/cid/ciaa375 | No | - | No | - | | 10 | |  |
| Ren ZL, et al. | Epidemiologic and Clinical Characteristics of Heart Transplant Recipients During the 2019 Coronavirus Outbreak in Wuhan, China: A Descriptive Survey Report | 10.1016/j.healun.2020.03.008 | Yes | COPD: 1 (1.1%) | No | - | | 87 | |  |
| Richardson S et al. | Presenting Characteristics, Comorbidities, and Outcomes Among 5700 Patients Hospitalized With COVID-19 in the New York City Area. | 10.1001/jama.2020.6775 | Yes | COPD: 287 (5.4%) | Yes | 479 (9%) | | 5700 | |  |
| Rivera-Izquierdo M, et al. | Sociodemographic, clinical and laboratory factors on admission associated with COVID-19 mortality in hospitalized patients: A retrospective observational study | 10.1371/journal.pone.0235107 | Yes | Prior lung disease 50 (21%) | No | - | | 238 | |  |
| Rogado J, et al. | Covid-19 and lung cancer: A greater fatality rate? | 10.1016/j.lungcan.2020.05.034 | Yes | COPD: 9 (52.9%) | No | - | | 17 | |  |
| Romero-Sánchez CM, et al. | Neurologic manifestations in hospitalized patients with COVID-19: The ALBACOVID registry | 10.1212/WNL.0000000000009937 | No | - | No | - | | 841 | |  |
| Rong Y, et al. | Clinical characteristics and risk factors of mild-to-moderate COVID-19 patients with false-negative SARS-CoV-2 nucleic acid | 10.1002/jmv.26242 | Yes | COPD: 4 (8%) | No | - | | 50 | |  |
| Sabatino J, et al. | COVID-19 and Congenital Heart Disease: Results from a Nationwide Survey | 10.3390/jcm9061774 | No | - | No | - | | 9 | |  |
| Sabri A, et al. | Novel coronavirus disease 2019: predicting prognosis with a computed tomography-based disease severity score and clinical laboratory data | 10.20452/pamw.15422 | No | - | No | - | | 63 | |  |
| Salva EP, et al. | Epidemiological and clinical characteristics of patients with suspected COVID-19 admitted in Metro Manila, Philippines | 10.1186/s41182-020-00241-8 | Yes | Respiratory illness: 7 (16,6%) | Yes | 7 (16,6%)* same patients with other respiratory illness | | 42 | |  |
| San-Juan R, et al. | Incidence and clinical profiles of COVID-19 pneumonia in pregnant women: A single-centre cohort study from Spain | 10.1016/j.eclinm.2020.100407 | No | - | Yes | 4 (12.5%) | | 32 | |  |
| Satici C, et al. | Performance of pneumonia severity index and CURB-65 in predicting 30-day mortality in patients with COVID-19 | 10.1016/j.ijid.2020.06.038 | Yes | COPD: 28 (4.1%) | Yes | 43 (6.3%) | | 681 | |  |
| Sentilhes L, et al. | Coronavirus disease 2019 in pregnancy was associated with maternal morbidity and preterm birth | 10.1016/j.ajog.2020.06.022 | No | - | Yes | 5 (9.3%) | | 54 | |  |
| Shahriarirad R, et al. | Epidemiological and clinical features of 2019 novel coronavirus diseases (COVID-19) in the South of Iran | 10.1186/s12879-020-05128-x | Yes | COPD: 9 (8%) | Yes | 7 (6.2%) | | 113 | |  |
| Shalev N, et al. | Clinical characteristics and outcomes in people living with HIV hospitalized for COVID-19 | 10.1093/cid/ciaa635 | Yes | COPD: 8 (25.8%) | Yes | 8 (25.8%)* same patients with COPD | | 31 | |  |
| Shang W, et al. | The value of clinical parameters in predicting the severity of COVID-19 | 10.1002/jmv.26031 | Yes | Pulmonary disease: 12 (2.7%) | No | - | | 443 | |  |
| Shekerdemian LS, et al. | Characteristics and Outcomes of Children With Coronavirus Disease 2019 (COVID-19) Infection Admitted to US and Canadian Pediatric Intensive Care Units | 10.1001/jamapediatrics.2020.1948 | Yes | Chronic lung disease: 2 (4%) | No | - | | 48 | |  |
| Shi J, et al. | Lactate dehydrogenase and susceptibility to deterioration of mild COVID-19 patients: a multicenter nested case-control study | 10.1186/s12916-020-01633-7 | No | - | No | - | | 85 | |  |
| Shi M, et al. | Analysis of clinical features and outcomes of 161 patients with severe and critical COVID-19: A multicenter descriptive study | 10.1002/jcla.23415 | Yes | COPD: 11 (6.83%) | No | - | | 161 | |  |
| Shi Q, et al. | Clinical Characteristics and Risk Factors for Mortality of COVID-19 Patients With Diabetes in Wuhan, China: A Two-Center, Retrospective Study | 10.2337/dc20-0598 | Yes | Chronic pulmonary disease: 21 (6.8%) | No | - | | 306 | |  |
| Shi S, et al. | Characteristics and clinical significance of myocardial injury in patients with severe coronavirus disease 2019 | 10.1093/eurheartj/ehaa408 | Yes | COPD: 23 (3.4%) | No | - | | 671 | |  |
| Si D, et al. | Death, discharge and arrhythmias among patients with COVID-19 and cardiac injury | 10.1503/cmaj.200879 | Yes | COPD: 11 (6,47%) | No | - | | 170 | |  |
| Sigel K, et al. | Covid-19 and People with HIV Infection: Outcomes for Hospitalized Patients in New York City | 10.1093/cid/ciaa880 | Yes | Chronic lung disease 15 (3.04%) | No | - | | 493 | |  |
| Silva F, et al. | SARS-CoV-2 infection in kidney transplant recipients: Early report of five cases | 10.1111/tid.13394 | No | - | No | - | | 5 | |  |
| Simonnet A, et al. | High Prevalence of Obesity in Severe Acute Respiratory Syndrome Coronavirus-2 (SARS-CoV-2) Requiring Invasive Mechanical Ventilation | 10.1002/oby.22831 | No | - | No | - | | 124 | |  |
| Singh S & Khan A | Clinical Characteristics and Outcomes of COVID-19 Among Patients With Pre-Existing Liver Disease in United States: A Multi-Center Research Network Study | 10.1053/j.gastro.2020.04.064 | Yes | Chronic lower respiratory diseases: 100 (40%) | No | - | | 250 | |  |
| Singhvi A, et al. | Challenges in heart transplantation during COVID-19: A single-center experience | 10.1016/j.healun.2020.06.015 | No | - | No | - | | 22 | |  |
| Smith SM, et al. | Impaired glucose metabolism in patients with diabetes, prediabetes and obesity is associated with severe Covid-19 | 10.1002/jmv.26227 | Yes | COPD: 12 (6.5%) | Yes | 18 (9.8%) | | 184 | |  |
| Solís & Carreňo | COVID-19 Fatality and Comorbidity Risk Factors among Diagnosed Patients in Mexico | 10.1101/2020.04.21.20074591 | Yes | COPD: 202 (2.7%) | Yes | 270 (3.6%) | | 7497 | |  |
| Steinberg E, et al. | In Young Adults with COVID-19, Obesity Is Associated with Adverse Outcomes | 10.5811/westjem.2020.5.47972 | No | - | No | - | | 210 | |  |
| Sultan I, et al. | The Role of Extracorporeal Life Support for Patients With COVID-19: Preliminary Results From a Statewide Experience | 10.1111/jocs.14583 | No | - | Yes | N/A | | 10 | |  |
| Sun D, et al. | SARS-CoV-2 infection in infants under 1 year of age in Wuhan City, China | 10.1007/s12519-020-00368-y | No | - | No | - | | 36 | |  |
| Sun H, et al. | Risk Factors for Mortality in 244 Older Adults With COVID-19 in Wuhan, China: A Retrospective Study | 10.1111/jgs.16533 | Yes | Previous respiratory diseases: 24 (9.8%) | No | - | | 244 | |  |
| Sun L, et al. | Clinical Features of Patients With Coronavirus Disease 2019 (COVID-19) From a Designated Hospital in Beijing, China | 10.1002/jmv.25966 | Yes | Respiratory diseases: 4 (7.3%) | No | - | | 55 | |  |
| Sun Y, et al. | Epidemiological and Clinical Predictors of COVID-19 | 10.1093/cid/ciaa322 | Yes | COPD: 10 (1.3%) | No | - | | 788 | |  |
| Szabados B, et al. | Clinical Characteristics and Outcome for Four SARS-CoV-2-infected Cancer Patients Treated with Immune Checkpoint Inhibitors | 10.1016/j.eururo.2020.05.024 | No | - | No | - | | 4 | |  |
| Tabata S, et al. | Clinical characteristics of COVID-19 in 104 people with SARS-CoV-2 infection on the Diamond Princess cruise ship: a retrospective analysis | 10.1016/S1473-3099(20)30482-5 | Yes | Respiratory disorder: 7 (7%) | No | - | | 104 | |  |
| Tan C, et al. | C-reactive Protein Correlates With CT Findings and Predicts Severe COVID-19 Early | 10.1002/jmv.25871 | No | - | No | - | | 27 | |  |
| Tan W, et al. | Single Center Trends in Acute Coronary Syndrome Volume and Outcomes During the COVID-19 Pandemic | 10.14740/cr1096 | No | - | No | - | | 204 | |  |
| Tang B, et al. | Coronavirus Disease 2019 (COVID-19) Pneumonia in a Hemodialysis Patient | 10.1016/j.xkme.2020.03.001 | No | - | No | - | | 1 | |  |
| Taxonera C, et al. | 2019 novel coronavirus disease (COVID-19) in patients with inflammatory bowel diseases | 10.1111/apt.15804 | No | - | No | - | | 12 | |  |
| Tian J, et al. | Clinical characteristics and risk factors associated with COVID-19 disease severity in patients with cancer in Wuhan, China: a multicentre, retrospective, cohort study | 10.1016/S1470-2045(20)30309-0 | Yes | COPD: 4 (1%) | No | - | | 751 | |  |
| Vizcarra P, et al. | Description of COVID-19 in HIV-infected individuals: a single-centre, prospective cohort | 10.1016/S2352-3018(20)30164-8 | Yes | Chronic respiratory disease: 8 (23%) | No | - | | 35 | |  |
| Vuagnat P, et al. | COVID-19 in breast cancer patients: a cohort at the Institut Curie hospitals in the Paris area | 10.1186/s13058-020-01293-8 | Yes | Chronic lung disease: 2 (3%) | No | - | | 59 | |  |
| Wan S, et al. | Clinical Features and Treatment of COVID-19 Patients in Northeast Chongqing | 10.1002/jmv.25783 | Yes | Pulmonary disease: 1 (0.7%) | No | - | | 135 | |  |
| Wan Y, et al. | Prognosis analysis of patients with mental disorders with COVID-19: a single-center retrospective study | 10.18632/aging.103371 | Yes | Chronic bronchitis: 1 (3,3%) | No | - | | 30 | |  |
| Wang A, et al. | Stroke and mechanical thrombectomy in patients with COVID-19: technical observations and patient characteristics | 10.1136/neurintsurg-2020-016220 | No | - | No | - | | 5 | |  |
| Wang C, et al. | Alveolar macrophage dysfunction and cytokine storm in the pathogenesis of two severe COVID-19 patients | 10.1016/j.ebiom.2020.102833 | No | - | No | - | | 2 | |  |
| Wang D, et al. | Clinical Characteristics of 138 Hospitalized Patients With 2019 Novel Coronavirus-Infected Pneumonia in Wuhan, China | 10.1001/jama.2020.1585 | Yes | COPD: 4 (2.9%) | No | - | | 138 | |  |
| Wang F, et al. | Characteristics of Peripheral Lymphocyte Subset Alteration in COVID-19 Pneumonia | 10.1093/infdis/jiaa150 | No | - | No | - | | 60 | |  |
| Wang F, et al. | Clinical characteristics of 28 patients with diabetes and COVID-19 in Wuhan, China | 10.4158/EP-2020-0108 | Yes | Chronic pulmonary disease: 2 (14.3%) | No | - | | 28 | |  |
| Wang H, et al. | Neutrophil to CD4+ lymphocyte ratio as a potential biomarker in predicting virus negative conversion time in COVID-19 | 10.1016/j.intimp.2020.106683 | Yes | Respiratory disease: 6 (6.3%) | No | - | | 95 | |  |
| Wang J, et al. | Clinical and CT findings of COVID-19: differences among three age groups | 10.1186/s12879-020-05154-9 | Yes | COPD: 3 (1%) | No | - | | 307 | |  |
| Wang J, et al. | Clinical characteristics of invasive pulmonary aspergillosis in patients with COVID-19 in Zhejiang, China: a retrospective case series | 10.1186/s13054-020-03046-7 | Yes | COPD: 4 (3.8%) | No | - | | 104 | |  |
| Wang K, et al. | Differences of Severe Acute Respiratory Syndrome Coronavirus 2 Shedding Duration in Sputum and Nasopharyngeal Swab Specimens Among Adult Inpatients With Coronavirus Disease 2019 | 10.1016/j.chest.2020.06.015 | Yes | Chronic lung disease: 12 (17.6%) | No | - | | 68 | |  |
| Wang K, et al. | Imaging manifestations and diagnostic value of chest CT of coronavirus disease 2019 (COVID-19) in the Xiaogan area | 10.1016/j.crad.2020.03.004 | Yes | Respiratory diseases: 5 (4.4%) | No | - | | 114 | |  |
| Wang L, et al. | Coronavirus disease 2019 in elderly patients: Characteristics and prognostic factors based on 4-week follow-up | 10.1016/j.jinf.2020.03.019 | Yes | COPD: 21 (6.2%) | No | - | | 339 | |  |
| Wang M, et al. | Typical Radiological Progression and Clinical Features of Patients With Coronavirus Disease 2019 | 10.18632/aging.103170 | Yes | COPD: 1 (2%) | No | - | | 66 | |  |
| Wang R, et al. | COVID-19 in Hemodialysis Patients: A Report of 5 Cases | 10.1053/j.ajkd.2020.03.009 | No | - | No | - | | 5 | |  |
| Wang X, et al. | Neutralizing Antibodies Responses to SARS-CoV-2 in COVID-19 Inpatients and Convalescent Patients | 10.1093/cid/ciaa721 | No | - | No | - | | 70 | |  |
| Wang X, et al. | Clinical characteristics of non-critically ill patients with novel coronavirus infection (COVID-19) in a Fangcang Hospital | 10.1016/j.cmi.2020.03.032 | Yes | Respiratory diseases: 20 (2.0%) | No | - | | 1012 | |  |
| Wang X, et al. | Clinical Characteristics of 80 Hospitalized Frontline Medical Workers Infected With COVID-19 in Wuhan, China | 10.1016/j.jhin.2020.04.019 | Yes | COPD: 1 (1.25%) | No | - | | 80 | |  |
| Wang X, et al. | Nosocomial outbreak of COVID-19 pneumonia in Wuhan, China. | 10.1183/13993003.00544-2020 | No | - | Yes | 2 (5.7%) | | 35 | |  |
| Wang Y, et al. | Characterization of an asymptomatic cohort of SARS-COV-2 infected individuals outside of Wuhan, China | 10.1093/cid/ciaa629 | Yes | COPD: 2 (1.58%) | No | - | | 126 | |  |
| Wang Y, et al. | Clinical Characteristics of Patients Infected With the Novel 2019 Coronavirus (SARS-Cov-2) in Guangzhou, China | 10.1093/ofid/ofaa187 | No | - | No | - | | 275 | |  |
| Wang Z, et al. | Clinical characteristics and laboratory results of pregnant women with COVID-19 in Wuhan, China | 10.1002/ijgo.13265 | No | - | No | - | | 72 | |  |
| Wang Z, et al. | Clinical Characteristics and Therapeutic Procedure for Four Cases With 2019 Novel Coronavirus Pneumonia Receiving Combined Chinese and Western Medicine Treatment | 10.5582/bst.2020.01030 | No | - | No | - | | 4 | |  |
| Wu C, et al. | Risk Factors Associated With Acute Respiratory Distress Syndrome and Death in Patients With Coronavirus Disease 2019 Pneumonia in Wuhan, China | 10.1001/jamainternmed.2020.0994 | Yes | Chronic lung diseases: 5 (2.5%) | No | - | | 201 | |  |
| Wu J, et al. | Clinical Features of Maintenance Hemodialysis Patients with 2019 Novel Coronavirus-Infected Pneumonia in Wuhan, China | 10.2215/CJN.04160320 | Yes | COPD: 1 (0.99%) | No | - | | 101 | |  |
| Wu J, et al. | Clinical Characteristics of Imported Cases of COVID-19 in Jiangsu Province: A Multicenter Descriptive Study | 10.1093/cid/ciaa199 | Yes | Respiratory system diseases: 1 (1.25%) | No | - | | 80 | |  |
| Wu J, et al. | Early Antiviral Treatment Contributes to Alleviate the Severity and Improve the Prognosis of Patients With Novel Coronavirus Disease (COVID-19) | 10.1111/joim.13063 | Yes | Respiratory system diseases: 6 (2.14%) and COPD: 1 (0.36%) | No | - | | 280 | |  |
| Wu X, et al. | Radiological findings and clinical characteristics of pregnant women with COVID-19 pneumonia | 10.1002/ijgo.13165 | No | - | No | - | | 23 | |  |
| Wu Y, et al. | Clinical outcomes of 402 patients with COVID-2019 from a single center in Wuhan, China | 10.1002/jmv.26168 | Yes | COPD: 7 (5%) | No | - | | 141 | |  |
| Xi A, et al. | Epidemiological and clinical characteristics of discharged patients infected with SARS-CoV-2 on the Qinghai Plateau | 10.1002/jmv.26032 | No | - | No | - | | 18 | |  |
| Xia XY, et al. | Epidemiological and initial clinical characteristics of patients with family aggregation of COVID-19 | 10.1016/j.jcv.2020.104360 | No | - | No | - | | 10 | |  |
| Xiang F, et al. | Antibody Detection and Dynamic Characteristics in Patients With COVID-19 | 10.1093/cid/ciaa461 | Yes | Chronic lung disease: N/A | No | - | | 85 | |  |
| Xie H, et al. | Clinical characteristics of non-ICU hospitalized patients with coronavirus disease 2019 and liver injury: A retrospective study | 10.1111/liv.14449 | No | - | No | - | | 79 | |  |
| Xie J, et al. | A Predictive Nomogram for Predicting Improved Clinical Outcome Probability in Patients with COVID-19 in Zhejiang Province, China | 10.1016/j.eng.2020.05.014 | No | - | No | - | | 104 | |  |
| Xie J, et al. | Characteristics of Patients With Coronavirus Disease (COVID-19) Confirmed Using an IgM-IgG Antibody Test | 10.1002/jmv.25930 | No | - | No | - | | 56 | |  |
| Xie Y, et al. | Impact of Cardiovascular Disease on Clinical Characteristics and Outcomes of Coronavirus Disease 2019 (COVID-19) | 10.1253/circj.CJ-20-0348 | No | - | No | - | | 62 | |  |
| Xiong F, et al. | Clinical Characteristics of and Medical Interventions for COVID-19 in Hemodialysis Patients in Wuhan, China | 10.1681/ASN.2020030354 | Yes | COPD: 3 (3.1%) | No | - | | 131 | |  |
| Xu C, et al. | Imaging and Clinical Features of Patients With 2019 Novel Coronavirus SARS-CoV-2 | 10.1007/s00259-020-04735-9 | Yes | COPD: 1 (0.9%) | No | - | | 90 | |  |
| Xu T, et al. | Clinical Features and Dynamics of Viral Load in Imported and Non-Imported Patients With COVID-19 | 10.1016/j.ijid.2020.03.022 | Yes | Pulmonary disease: 1 (1.96%) | No | - | | 51 | |  |
| Xu T, et al. | Epidemiological and Clinical Features of Asymptomatic Patients With SARS-CoV-2 Infection | 10.1002/jmv.25944 | No | - | No | - | | 15 | |  |
| Xu X, et al. | Effective Treatment of Severe COVID-19 Patients With Tocilizumab | 10.1073/pnas.2005615117 | Yes | COPD: 1 (4.8%) and Bronchiectasis 1 (4.8%) | No | - | | 21 | |  |
| Xu XW, et al. | Clinical findings in a group of patients infected with the 2019 novel coronavirus (SARS-Cov-2) outside of Wuhan, China: retrospective case series | 10.1136/bmj.m606 | Yes | COPD: 1 (1.61%) | No | - | | 62 | |  |
| Yaghi S, et al. | SARS-CoV-2 and Stroke in a New York Healthcare System | 10.1161/STROKEAHA.120.030335 | No | - | No | - | | 32 | |  |
| Yang A, et al. | Clinical and Epidemiological Characteristics of COVID-19 Patients in Chongqing China | 10.3389/fpubh.2020.00244 | Yes | Respiratory system disease: 1 (0.87%) | No | - | | 144 | |  |
| Yang AP, et al. | Infection with SARS-CoV-2 causes abnormal laboratory results of multiple organs in patients | 10.18632/aging.103255 | No | - | No | - | | 93 | |  |
| Yang B, et al. | Impact of the COVID-19 pandemic on the process and outcome of thrombectomy for acute ischemic stroke | 10.1136/neurintsurg-2020-016177 | Yes | Pulmonary inflammation on chest CT: 6 (28.6%) | No | - | | 21 | |  |
| Yang F, et al. | Analysis of 92 deceased patients with COVID-19 | 10.1002/jmv.25891 | Yes | COPD: 1 (1.08%) | No | - | | 92 | |  |
| Yang F, et al. | Clinical characteristics and outcomes of cancer patients with COVID-19 | 10.1002/jmv.25972 | Yes | COPD: 1 (9.1%) | No | - | | 11 | |  |
| Yang K, et al. | Clinical characteristics, outcomes, and risk factors for mortality in patients with cancer and COVID-19 in Hubei, China: a multicentre, retrospective, cohort study | 10.1016/S1470-2045(20)30310-7 | Yes | COPD: 5 (2%) | No | - | | 205 | |  |
| Yang L, et al. | Epidemiological and clinical features of 200 hospitalized patients with corona virus disease 2019 outside Wuhan, China: A descriptive study | 10.1016/j.jcv.2020.104475 | Yes | Chronic lung disease: 7 (3.5 %) | No | - | | 200 | |  |
| Yang P, et al. | A retrospective study on the epidemiological characteristics and establishment of early warning system of severe COVID-19 patients | 10.1002/jmv.26022 | Yes | COPD: 4 (3%) | No | - | | 133 | |  |
| Yang Q, et al. | Analysis of the clinical characteristics, drug treatments and prognoses of 136 patients with coronavirus disease 2019 | 10.1111/jcpt.13170 | No | - | No | - | | 136 | |  |
| Yang S, et al. | Clinical Characteristics of COVID-19 After Gynecologic Oncology Surgery in Three Women: A Retrospective Review of Medical Records | 10.1634/theoncologist.2020-0157 | No | - | No | - | | 3 | |  |
| Yang W, et al. | Clinical Characteristics and Imaging Manifestations of the 2019 Novel Coronavirus Disease (COVID-19):A Multi-Center Study in Wenzhou City, Zhejiang, China | 10.1016/j.jinf.2020.02.016 | Yes | Respiratory system disease: 1 (0.67%) | No | - | | 149 | |  |
| Yang Y, et al. | Plasma IP-10 and MCP-3 Levels Are Highly Associated With Disease Severity and Predict the Progression of COVID-19 | 10.1016/j.jaci.2020.04.027 | Yes | Chronic lung disease: 2 (6.7%) | No | - | | 30 | |  |
| Yao Q, et al. | Retrospective Study of Risk Factors for Severe SARS-Cov-2 Infections in Hospitalized Adult Patients | 10.20452/pamw.15312 | Yes | Pulmonary disease (bronchiectasis, COPD or asthma): 3 (2.8%) | Yes | 3 (2.8%)* same patients with other pulmonary diseases | | 108 | |  |
| Yao Y, et al. | Clinical characteristics of COVID-19 patients in three consecutive generations of spread in Zhejiang, China | 10.1016/j.cmi.2020.06.018 | Yes | COPD: 3 (1.8%) | No | - | | 171 | |  |
| Yarza R, et al. | SARS-CoV-2 infection in cancer patients undergoing active treatment: analysis of clinical features and predictive factors for severe respiratory failure and death | 10.1016/j.ejca.2020.06.001 | Yes | Chronic pulmonary disease 14 (22%) | No | - | | 63 | |  |
| Ye F, et al. | Delivery of Infection From Asymptomatic Carriers of COVID-19 in a Familial Cluster | 10.1016/j.ijid.2020.03.042 | No | - | No | - | | 5 | |  |
| Ye G, et al. | Clinical Characteristics of Severe Acute Respiratory Syndrome Coronavirus 2 Reactivation | 10.1016/j.jinf.2020.03.001 | No | - | No | - | | 55 | |  |
| Yin Z, et al. | A Comparison of Clinical and Chest CT Findings in Patients With Influenza A (H1N1) Virus Infection and Coronavirus Disease (COVID-19) | 10.2214/AJR.20.23214 | Yes | Chronic respiratory disease: 0 | No | - | | 30 | |  |
| Yoshimura Y, et al. | Clinical characteristics of the coronavirus disease 2019 (COVID-19) outbreak on a cruise ship | 10.1016/j.jiac.2020.06.010 | Yes | Chronic respiratory diseases: 6 (35,29%) ** | Yes | 6 (35,29%)** | | 17 | |  |
| Yu C, et al. | Clinical Characteristics, Associated Factors, and Predicting COVID-19 Mortality Risk: A Retrospective Study in Wuhan, China | 10.1016/j.amepre.2020.05.002 | Yes | COPD: 50 (3.4%) | No | - | | 1464 | |  |
| Yu M, et al. | Prediction of the Development of Pulmonary Fibrosis Using Serial Thin-Section CT and Clinical Features in Patients Discharged after Treatment for COVID-19 Pneumonia | 10.3348/kjr.2020.0215 | Yes | COPD: 1 (3.1%) | No | - | | 32 | |  |
| Yu N, et al. | Clinical Features and Obstetric and Neonatal Outcomes of Pregnant Patients With COVID-19 in Wuhan, China: A Retrospective, Single-Centre, Descriptive Study | 10.1016/S1473-3099(20)30176-6 | No | - | No | - | | 7 | |  |
| Yu Q, et al. | Multicenter Cohort Study Demonstrates More Consolidation in Upper Lungs on Initial CT Increases the Risk of Adverse Clinical Outcome in COVID-19 Patients | 10.7150/thno.46465 | No | - | No | - | | 625 | |  |
| Yu X, et al. | Epidemiological and Clinical Characteristics of 333 Confirmed Cases With Coronavirus Disease 2019 in Shanghai, China | 10.1111/tbed.13604 | Yes | Respiratory diseases: 5 (1.5%) | No | - | | 333 | |  |
| Yu Y, et al. | Patients with COVID-19 in 19 ICUs in Wuhan, China: a cross-sectional study. | 10.1186/s13054-020-02939-x | Yes | Chronic pulmonary disease: 15 (6.6%) | No | - | | 226 | |  |
| Yuan M, et al. | Association of Radiologic Findings With Mortality of Patients Infected With 2019 Novel Coronavirus in Wuhan, China | 10.1371/journal.pone.0230548 | No | - | No | - | | 27 | |  |
| Zhang C, et al. | Clinical and epidemiological characteristics of pediatric SARS-CoV-2 infections in China: A multicenter case series | 10.1371/journal.pmed.1003130 | No | - | Yes | 1 (3%) | | 34 | |  |
| Zhang H, et al. | Identification of Kidney Transplant Recipients With Coronavirus Disease 2019 | 10.1016/j.eururo.2020.03.030 | No | - | No | - | | 5 | |  |
| Zhang J, et al. | Poor-sleep is associated with slow recovery from lymphopenia and an increased need for ICU care in hospitalized patients with COVID-19: A retrospective cohort study | 10.1016/j.bbi.2020.05.075 | Yes | Chronic respiratory disease: 6 (4.4%) | No | - | | 135 | |  |
| Zhang J, et al. | Do underlying cardiovascular diseases have any impact on hospitalised patients with COVID-19? | 10.1136/heartjnl-2020-316909 | Yes | Respiratory system disease: 45 (8.3%) | No | - | | 541 | |  |
| Zhang J, et al. | Predictive Factors for Disease Progression in Hospitalized Patients With Coronavirus Disease 2019 in Wuhan, China | 10.1016/j.jcv.2020.104392 | Yes | COPD: 3 (2.7 %) | No | - | | 111 | |  |
| Zhang J, et al. | The Clinical Characteristics and Prognosis Factors of Mild-Moderate Patients With COVID-19 in a Mobile Cabin Hospital: A Retrospective, Single-Center Study | 10.3389/fpubh.2020.00264 | Yes | COPD: 11 (1.3%) | Yes | 11 (1.3%)* same patients with COPD | | 869 | |  |
| Zhang JJ, et al. | Clinical characteristics of 140 patients infected with SARS-CoV-2 in Wuhan, China | 10.1111/all.14238 | Yes | COPD: 2 (1.4%) | Yes | 0 | | 140 | |  |
| Zhang L, et al. | Clinical Characteristics of COVID-19-infected Cancer Patients: A Retrospective Case Study in Three Hospitals Within Wuhan, China | 10.1016/j.annonc.2020.03.296 | Yes | COPD: 1 (3.5%) | Yes | 1 (3.5%)* same patients with COPD | | 28 | |  |
| Zhang L, et al. | Diarrhea and altered inflammatory cytokine pattern in severe coronavirus disease 2019: Impact on disease course and in‐hospital mortality | 10.1111/jgh.15166 | Yes | COPD: 23 (5.6%) | No | - | | 409 | |  |
| Zhang L, et al. | D-dimer levels on admission to predict in-hospital mortality in patients with Covid-19 | 10.1111/jth.14859 | Yes | COPD: 8 (2.3%) | No | - | | 343 | |  |
| Zhang N, et al. | Clinical characteristics and chest CT imaging features of critically ill COVID-19 patients | 10.1007/s00330-020-06955-x | Yes | COPD: 6 (10%) | No | - | | 60 | |  |
| Zhang P, et al. | The modified NUTRIC score can be used for nutritional risk assessment as well as prognosis prediction in critically ill COVID-19 patients | 10.1016/j.clnu.2020.05.051 | Yes | COPD: 12 (9%) | No | - | | 136 | |  |
| Zhang Q, et al. | Clinical analysis of risk factors for severe COVID-19 patients with type 2 diabetes | 10.1016/j.jdiacomp.2020.107666 | Yes | Secondary pulmonary tuberculosis 12 (16.2%) | No | - | | 74 | |  |
| Zhang X, et al. | Epidemiological, Clinical Characteristics of Cases of SARS-CoV-2 Infection With Abnormal Imaging Findings | 10.1016/j.ijid.2020.03.040 | Yes | COPD: 1 (0.15%) | No | - | | 645 | |  |
| Zhang Y, et al. | Manifestations of blood coagulation and its relation to clinical outcomes in severe COVID-19 patients: Retrospective analysis | 10.1111/ijlh.13273 | Yes | Chronic respiratory disease: 4 (5.6%) | No | - | | 71 | |  |
| Zhang Y, et al. | The clinical characteristics and outcomes of patients with diabetes and secondary hyperglycaemia with coronavirus disease 2019: A single-centre, retrospective, observational study in Wuhan | 10.1111/dom.14086 | Yes | COPD: 19 (11.4%) | No | - | | 116 | |  |
| Zhao J, et al. | Retrospective Analysis of the Clinical and Epidemiological Characteristics of COVID-19 Patients in Henan Provincial People's Hospital, Zhengzhou, China | 10.3389/fmed.2020.00286 | Yes | Respiratory system disease 4 (13.8%) | No | - | | 29 | |  |
| Zhao M, et al. | Comparison of clinical characteristics and outcomes of patients with coronavirus disease 2019 at different ages | 10.18632/aging.103298 | Yes | COPD: 23 (2.3%) | Yes | 12 (1.2%) | | 1000 | |  |
| Zhao S, et al. | Anesthetic Management of Patients with COVID 19 Infections during Emergency Procedures | 10.1053/j.jvca.2020.02.039 | Yes | Respiratory system diseases: 8 (22%) | No | - | | 37 | |  |
| Zhao W, et al. | Relation Between Chest CT Findings and Clinical Conditions of Coronavirus Disease (COVID-19) Pneumonia: A Multicenter Study | 10.2214/AJR.20.22976 | Yes | Respiratory system diseases: 5 (4.9%) | No | - | | 101 | |  |
| Zhao W, et al. | CT Scans of Patients With 2019 Novel Coronavirus (COVID-19) Pneumonia | 10.7150/thno.45016 | Yes | Respiratory system diseases: 4 (3.4%) | No | - | | 118 | |  |
| Zhao X, et al. | Clinical Characteristics of Patients With 2019 Coronavirus Disease in a non-Wuhan Area of Hubei Province, China: A Retrospective Study | 10.1186/s12879-020-05010-w | Yes | COPD: 1 (1,09%) | No | - | | 91 | |  |
| Zheng F, et al. | Clinical Characteristics of 161 Cases of Corona Virus Disease 2019 (COVID-19) in Changsha | 10.26355/eurrev_202003_20711 | Yes | COPD: 6 (3.7%) | No | - | | 161 | |  |
| Zheng F, et al. | Clinical Characteristics of Children With Coronavirus Disease 2019 in Hubei, China | 10.1007/s11596-020-2172-6 | No | - | No | - | | 25 | |  |
| Zheng S, et al. | Viral Load Dynamics and Disease Severity in Patients Infected With SARS-CoV-2 in Zhejiang Province, China, January-March 2020: Retrospective Cohort Study | 10.1136/bmj.m1443 | Yes | Lung disease: 4 (4%) | No | - | | 96 | |  |
| Zheng Y, et al. | Epidemiological and Clinical Characteristics Analysis of COVID-19 in the Surrounding Areas of Wuhan, Hubei Province in 2020 | 10.1016/j.phrs.2020.104821 | Yes | Sinusitis and pleurisy: 4 (5.5%) | No | - | | 73 | |  |
| Zheng Y, et al. | The Hemocyte Counts as a Potential Biomarker for Predicting Disease Progression in COVID-19: A Retrospective Study | 10.1515/cclm-2020-0377 | No | - | No | - | | 141 | |  |
| Zhong Q, et al. | Spinal anaesthesia for patients with coronavirus disease 2019 and possible transmission rates in anaesthetists: retrospective, single-centre, observational cohort study | 10.1016/j.bja.2020.03.007 | No | - | No | - | | 49 | |  |
| Zhong Z, et al. | Clinical Characteristics and Immunosuppressants Management of Coronavirus Disease 2019 in Solid Organ Transplant Recipients | 10.1111/ajt.15928 | No | - | No | - | | 2 | |  |
| Zhou F, et al. | Clinical Course and Risk Factors for Mortality of Adult Inpatients With COVID-19 in Wuhan, China: A Retrospective Cohort Study | 10.1016/S0140-6736(20)30566-3 | Yes | Chronic obstructive lung disease: 6 (3%) | No | - | | 191 | |  |
| Zhou H, et al. | COVID-19 Is Distinct From SARS-CoV-2-Negative Community-Acquired Pneumonia | 10.3389/fcimb.2020.00322 | Yes | COPD: 7 (2.3%) | No | - | | 304 | |  |
| Zhou S, et al. | CT Features of Coronavirus Disease 2019 (COVID-19) Pneumonia in 62 Patients in Wuhan, China | 10.2214/AJR.20.22975 | No | - | No | - | | 62 | |  |
| Zhou X, et al. | Clinical Characteristics of Coronavirus Disease 2019 (COVID-19) Patients With Hypertension on Renin-Angiotensin System Inhibitors | 10.1080/10641963.2020.1764018 | Yes | COPD:3 (2.7%) | Yes | 1 (0.9%) | | 110 | |  |
| Zhou Y, et al. | Clinical and Autoimmune Characteristics of Severe and Critical Cases of COVID-19 | 10.1111/cts.12805 | Yes | COPD: 2 (9.5%) | No | - | | 21 | |  |
| Zhou Z, et al. | Clinical characteristics of older and younger patients infected with SARS-CoV-2 | 10.18632/aging.103535 | Yes | COPD: 5 (2.1%) | No | - | | 239 | |  |
| Zhu L, et al. | Coronavirus Disease 2019 Pneumonia in Immunosuppressed Renal Transplant Recipients: A Summary of 10 Confirmed Cases in Wuhan, China | 10.1016/j.eururo.2020.03.039 | Yes | COPD: 1 (10%) | No | - | | 10 | |  |
| Zhu Z, et al. | Clinical value of immune-inflammatory parameters to assess the severity of coronavirus disease 2019 | 10.1016/j.ijid.2020.04.041 | Yes | Chronic lung disease: 6 (4.72%) | No | - | | 127 | |  |
| Zou X, et al. | Characteristics of Liver Function in Patients With SARS-CoV-2 and Chronic HBV Coinfection | 10.1016/j.cgh.2020.06.017 | Yes | COPD: 3 (2.86%) | No | - | | 105 | |  |

** Chronic obstructive pulmonary disease, bronchial asthma, or interstitial pneumonia

*** Chronic obstructive pulmonary disease, asthma, interstitial lung disease or other not specified

**** Bronchiectasis, chronic obstructive pulmonary disease, or asthma
